# Microbiota-Dependent Fiber Responses: A Proof-of-Concept Study on Short-Chain Fatty Acid Production in *Prevotella*- and *Bacteroides*-Dominated Healthy Individuals

**DOI:** 10.1016/j.tjnut.2025.08.034

**Published:** 2025-09-02

**Authors:** Madeline Bartsch, Marius Vital, Sabrina Woltemate, Freek G Bouwman, Shoma B Berkemeyer, Andreas Hahn, Mattea Müller

**Affiliations:** 1 Institute of Food and One Health, Leibniz University Hannover, Hannover, Germany; 2 NutritionLab, Faculty of Agricultural Sciences and Landscape Architecture, Osnabrueck University of Applied Sciences, Osnabrueck, Germany; 3 Institute for Medical Microbiology and Hospital Epidemiology, Hannover Medical School, Hannover, Germany; 4 Department of Human Biology, NUTRIM School of Nutrition and Translational Research in Metabolism, Maastricht University Medical Center, Maastricht, The Netherlands

**Keywords:** personalized nutrition, enterotypes, fiber responsiveness, branched-chain fatty acids, microbiota–diet interactions

## Abstract

**Background:**

Dietary fiber supports metabolic health via microbial fermentation, producing short-chain fatty acids (SCFAs). However, metabolic responses to fiber vary between individuals, potentially due to differences in gut microbiota composition. The *Prevotella*-to-*Bacteroides* ratio has emerged as a potential biomarker for fiber responsiveness.

**Objectives:**

This study examined how stratified fiber supplementation affects microbial and metabolic outcomes in individuals with *Prevotella* (P-type)- or *Bacteroides*-dominated (B-type) microbiota.

**Methods:**

In this single-blinded, randomized crossover study, 23 healthy adults were classified as P-type (≥10% *Prevotella*) or B-type (≥10% *Bacteroides*) via 16S rRNA sequencing. Participants consumed 15 g/d of arabinoxylan (AX), inulin (INU), or placebo (PLA) for 1 wk each, with 2-wk washouts. After each phase, fasting and postprandial plasma SCFAs, branched-chain fatty acids (BCFAs), breath hydrogen, glucose, insulin, peptide YY, cholesterol, appetite ratings, and fecal microbiota were assessed. Data were analyzed using repeated-measures analysis of variance, the Friedman test, and multivariate microbiome analysis.

**Results:**

In P-types, AX increased fasting propionate compared with PLA (*P* = 0.04). In B-types, AX increased fasting propionate compared with INU (*P* = 0.02) and tended to elevate postprandial propionate compared with PLA in the first 60 min after breakfast (*P* = 0.05). AX also increased postprandial acetate compared with PLA in B-types (*P* = 0.04). INU reduced fasting BCFAs in B-types (*P* < 0.05) but did not increase SCFAs. Breath hydrogen varied widely in B-types after INU but not in P-types. Neither fiber affected glucose, insulin, or PYY. AX reduced appetite ratings in P-types (*P* < 0.05). INU increased *Anaerostipes* and *Bifidobacterium* and reduced *Phocaeicola* in both groups (*q* < 0.25). AX increased *Fusicatenibacter* in B-types (*q* = 0.18) and *Paraprevotella* in P-types (*q* = 0.17).

**Conclusions:**

B-types exhibited fiber-specific shifts in SCFA and BCFA metabolism and breath hydrogen, whereas P-types displayed a more limited overall response, with fewer metabolic and microbial parameters affected. These findings highlight the complexity of diet–microbiota interactions and support the potential relevance for microbiota-based nutrition strategies.

This trial (PERIFIB) was registered at the German Clinical Trials register (DRKS) as DRKS00028898.

## Graphical abstract

**Figure:**
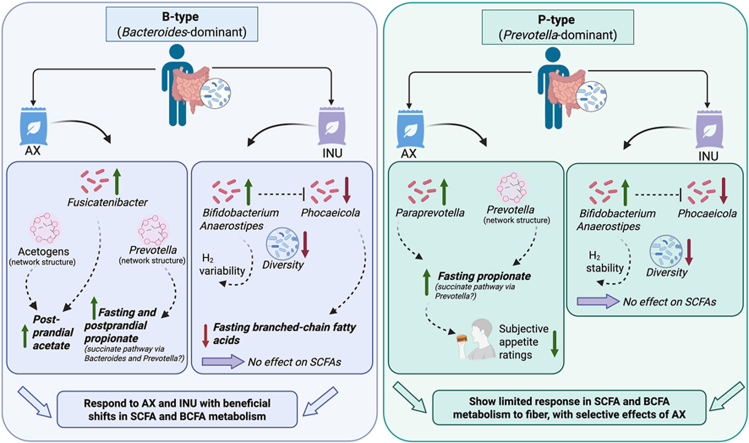

## Introduction

The human gut microbiome plays a pivotal role in metabolic health by influencing energy metabolism, immune regulation, and inflammatory processes [1]. Diet, especially dietary fibers, is a major modulator of gut microbiota composition and function [2]. Fiber intake has been associated with improved glycemic control, increased insulin sensitivity, reduced cholesterol concentrations, and increased satiety [3,4]. Despite these well-established metabolic benefits of dietary fiber from epidemiologic and mechanistic studies, human intervention studies have reported inconsistent effects on gut microbiota and metabolic outcomes [[5], [6], [7], [8]]. For example, trans-galactooligosaccharides reduced plasma insulin, cholesterol, and triglyceride concentrations, and increased *Bifidobacterium* abundance [5]. In contrast, inulin (INU)-type fructans and galactooligosaccharides altered microbiota composition without significantly improving insulin sensitivity or glycemic control [[6], [7], [8]]. These discrepancies may arise from structural differences in fiber, including chain length, glycosidic linkages, and branching patterns, which determine their accessibility to specific microbial enzymes, influence fermentation kinetics, and modulate cross-feeding interactions within the gut microbiota [9]. Increasing evidence also suggests that an individual’s gut microbiota composition may be crucial for metabolic response to specific dietary fibers [10,11]. One framework for understanding interindividual differences in microbiota composition is the enterotype concept. Although the concept has been subject to criticism due to methodological variability [12], limited clinical utility [13], and the observation that microbial compositions may exist on a continuum rather than in discrete groups [13,14], it still provides a useful framework for stratifying individuals based on their dominant taxa [15]. *Prevotella* and *Bacteroides* are often inversely correlated among the most studied enterotype-associated genera, with 1 genus typically dominating over the other in an individual [9]. *Prevotella* and *Bacteroides* are 2 predominant members of the commensal gut microbiota, known for their distinct roles in the fermentation of dietary fiber and metabolic regulation [16]. Christensen et al. [17] proposed that the *Prevotella*-to-*Bacteroides* (P/B)-ratio may predict metabolic responses to fiber interventions. Post hoc analyses of randomized controlled trials suggest that individuals with a high P/B ratio lose more weight on high-fiber diets than individuals with a low P/B ratio [[18], [19], [20]], likely due to functional differences between the respective microbial communities classified as *Prevotella*-dominant (P-type) or *Bacteroides*-dominant (B-type). P-types have an enhanced ability to degrade plant fibers due to a high abundance of hydrolases specialized in polysaccharide degradation, while exhibiting a lower proteolytic and lipolytic fermentation capacity. In contrast, B-types exhibit greater proteolytic activity and have been associated with lower microbial diversity and reduced functional redundancy, which may make them more vulnerable to dietary disturbances [13,21,22]. These functional differences may contribute to distinct metabolic responses to dietary fiber supplementation, primarily through variations in microbial fermentation capacity. The fermentation of dietary fibers by gut microbes results in the production of short-chain fatty acids (SCFAs), primarily acetate, butyrate, and propionate [23]. These microbial metabolites function as key signaling molecules, influencing energy homeostasis, glucose metabolism, and inflammation. SCFAs interact with G protein-coupled receptors on enteroendocrine cells, promoting the release of glucagon-like peptide 1 (GLP-1) and peptide YY (PYY), which regulate insulin secretion, glucose metabolism, and satiety [24]. Additionally, SCFAs contribute to body weight regulation by modulating energy intake, expenditure, and insulin sensitivity [25,26]. Altered SCFA production has been linked to metabolic disorders such as obesity and type 2 diabetes mellitus [27]. Importantly, plasma SCFA concentrations have been associated with metabolic outcomes, whereas fecal SCFA concentrations primarily reflect microbial fermentation activity in the gut rather than systemic effects [28]. Evidence from in vitro fermentation models supports mechanistic differences between P-types and B-types. Fermentation of arabinoxylans (AXs) from grain bran with P-type fecal samples resulted in significantly higher propionate production than with B-type samples [29]. Although both *Bacteroides* and *Prevotella* can ferment AXs, the high propionate production in *Prevotella*-dominated individuals suggests that *Prevotella* plays the predominant role in their fermentation. Due to its specialized enzymatic capacity [17], *Prevotella* may ferment AXs more efficiently, resulting in increased propionate production and potential metabolic benefits such as glucose homeostasis and satiety signaling. Conversely, findings by Gu et al. [30] indicate that individuals with a high abundance of *Bacteroides* may benefit more from bifidogenic prebiotic supplementation, such as INU, as *Bifidobacteria* proliferation has been linked to improved metabolic parameters in B-types.

Building on this evidence, we investigated how stratified dietary fiber interventions influence metabolic and microbial outcomes in individuals with different dominant gut microbiota profiles. We conducted a randomized, placebo-controlled crossover trial to assess the effects of a 7-d supplementation with AX and INU on SCFA production in individuals stratified into P-types or B-types before the intervention. We hypothesized that P-types would exhibit enhanced SCFA production in response to AX supplementation, whereas B-types would benefit more from bifidogenic prebiotic INU. Secondary outcomes included glycemic and insulinemic responses, breath hydrogen, and appetite regulation.

## Methods

### Study participants

Healthy adults aged 30–65 y were recruited in Hanover, Germany, and surrounding areas between May 2022 and October 2023 via local media, social platforms, pharmacies, and medical practices. Study procedures were conducted at the Institute of Food and One Health, Leibniz University Hannover. Eligibility was assessed during a screening visit, which included anthropometrics, blood sampling (details in **Supplemental Methods**), stool sampling, questionnaires on general health and medical history, and a food frequency questionnaire (FFQ). FFQ data were used to calculate Plant-based Diet Index, along with its associated healthy and unhealthy subscores according to Satija et al. [31]. Participants were eligible if they had a BMI between 20 and 28 kg/m^2^, fasting glucose ≤6.1 mmol/L, HOMA-IR ≤2, normal blood pressure (100–140/60–90 mmHg), followed an omnivorous or vegetarian diet, and showed a relative abundance ≥10% of either *Prevotella* or *Bacteroides* (based on fecal 16S rRNA gene sequencing), a threshold chosen to reflect clear microbial dominance [32]. The criteria for BMI, fasting blood glucose, HOMA-IR, and blood pressure were selected to ensure the inclusion of a general metabolically healthy population and minimize the potential confounding effects of metabolic disorders on gut microbiota composition and fermentation outcomes. Individuals with chronic diseases, prior abdominal surgery, medication use (e.g., anticoagulants), pregnancy, lactation, or recent use of antibiotics, probiotics, or prebiotics were excluded.

### Study design

This single-blinded, placebo-controlled crossover study included 3 intervention phases of 7-d each, separated by washout periods of ≥14 d to reduce potential carryover effects. An independent researcher randomized the sequence of interventions. Study products were identically packaged and sealed to ensure investigator blinding. Complete participant blinding was not feasible due to the distinct appearance of the AX supplement. Additionally, the maltodextrin placebo (PLA) dose was lower in weight than the fiber supplements to match their caloric content, which might have allowed some participants to notice a difference in portion size, although the identity of the intervention was not disclosed. Participants were instructed to maintain their habitual diet and physical activity and to report any changes in medication throughout the study. Compliance was evaluated by counting returned, unused product sachets at the end of each phase. The study was approved by the Ethics Committee of the Medical Association of Lower Saxony and registered in the German Clinical Trials Register (DRKS00028898). All procedures adhered to the Declaration of Helsinki (revised 2008), and written informed consent was obtained from all participants before enrollment.

### Study intervention

Each intervention phase consisted of a 7-d intake of 1 of 3 isocaloric study products (∼30 kcal/d) administered 3-time daily. Participants received either 15 g/d of long-chain INU, 15 g/d of alkali-extracted soluble AX derived from corn bran, or 7.95 g/d of PLA. AX and INU were selected based on their distinct fermentation profiles and hypothesized enterotype-specific effects. The final dose of each phase was administered ∼12 h before the clinical investigation day (CID). We chose the 7-d intervention period to capture early microbial fermentation and community changes, which can occur within days of introducing fermentable fibers [33]. The daily dose was divided into 3 administrations to provide a steadier substrate supply to the colon during the day and to improve gastrointestinal tolerability compared with a single bolus. Detailed product specifications and preparation instructions are provided in the Supplemental Methods.

### Clinical Investigation Day

On the morning after each 7-d intervention period, participants arrived at the study center after an overnight fast of ≥12 h. Participants provided a stool sample during each intervention period, either the day before the CID or on the morning of the CID. Participants also recorded stool consistency using the Bristol Stool Chart [34]. Stool samples were later used for microbiota profiling via 16S rRNA gene sequencing (Supplemental Methods). A visual overview of the CID procedures is provided in Supplemental Figure 1. Fasting and postprandial blood samples were collected at 30, 60, 120, and 180 min after the consumption of a standardized low-fiber breakfast (570 kcal; 64.4 g carbohydrates, 25.1 g fat, 20.0 g protein, and 3.42 g fiber) (Supplemental Table 1). Blood was processed for serum and plasma and stored at –80°C for subsequent analysis of glucose, insulin, PYY, cholesterol, and SCFA/branched-chain fatty acid (BCFA) concentrations (details in Supplemental Methods). Appetite sensations were assessed at the same time points using VAS (0–100 mm) for hunger, fullness, satisfaction, prospective food intake, and desire to eat. Breath hydrogen was measured every 15 min using a handheld analyzer (Bedfont EC60 Gastrolyzer) to evaluate fermentation-derived gas production. Gastrointestinal symptoms during the preceding 7 d were recorded using the Gastrointestinal Symptoms Rating Scale (GSRS), which includes 15 items across 5 symptom clusters, rated on a 7-point Likert scale [35]. Dietary intake before CID was analyzed via 24-h dietary recall using the MyFood24 nutritional assessment tool (https://myfood24.org), based on the German food database *Bundeslebensmittelschlüssel* (v3.02).

### Sample size calculation

The sample size was based on previous randomized crossover studies [36], assuming a 30% increase in circulating SCFA concentrations (SD = 5) as metabolically relevant. Power analysis (G∗Power v3.1) indicated that 9 participants per group would be required to achieve 80% power at α = 0.05. To account for an anticipated 20% dropout, 12 participants per group (*Prevotella*- and *Bacteroides*-dominant) were recruited.

### Statistical analysis of clinical parameters

Differences in baseline characteristics between B-type and P-type participants were analyzed using the Wilcoxon rank sum test (continuous variables) and Fisher's exact test (categorical variables). Postprandial responses were analyzed as total AUC (AUC_0-180_), as well as early (AUC_0-60_) and late (AUC_60-180_) phases. Fasting values and AUCs across interventions were compared using repeated-measures analysis of variance (ANOVA), with supplementation as a within-subject factor, participant ID as a random effect, and microbial group (B-type, P-type) as a between-subject factor. The order of interventions (sequence) was included as an additional independent variable to assess potential carryover effects; no significant sequence effects were observed. Model assumptions were checked and, if necessary, log-transformed (Supplemental Methods). Post hoc comparisons were performed when *P* < 0.1 using pairwise *t*-tests with Cohen’s d. Given the exploratory nature, least significant difference testing was applied. Stool consistency, stool frequency, and gastrointestinal symptoms (GSRS scores) were analyzed using Friedman tests and Eisinger post hoc comparisons. Dietary intake was analyzed by repeated-measures ANOVA, with macronutrient intake normalized per 1000 kcal. All analyses were performed in R (v4.4.1), with *P* < 0.05 considered statistically significant.

### Statistical analysis of microbiome

Microbiota analyses were based on the absolute abundance of amplicon sequencing variants. Alpha diversity (Shannon index, Chao1 richness) was assessed using rarefied data (5000 reads/sample) and compared across interventions using repeated-measures ANOVA. Beta diversity was evaluated using Bray–Curtis dissimilarity and visualized via principal coordinate analysis. Baseline differences between microbial groups were assessed using permutational multivariate ANOVA. To assess compositional changes across interventions, the longitudinal beta diversity test from the MicrobiomeStat package was applied. Differentially abundant genera were identified using MaAsLin2, which applied multivariable linear models to centered log-ratio transformed data. Genera with ≥10% prevalence and ≥1% relative abundance were included. Results were adjusted for multiple testing using the Benjamini–Hochberg method, with *P* < 0.25 considered significant as recommended [37]. Log_2_ fold changes were visualized as boxplots. Associations between genera and SCFA concentrations were examined using linear mixed-effects models with interaction terms for supplementation (Supplemental Methods for full model specifications and software versions).

### Microbial co-occurrence network inference

Co-occurrence networks were inferred using the LIMON pipeline, which accounts for repeated measures in longitudinal microbiome data [38] (Supplemental Methods). Treatment-specific networks (PLA, AX, and INU) were constructed based on partial correlations of genus-level abundances. Differential networks (AX–PLA, INU–PLA) were computed by subtracting adjacency matrices after confirming lambda equivalence. Subject-specific networks were also generated to assess centrality metrics (degree, closeness, betweenness, and eigenvector), which were compared across interventions using repeated-measures ANOVA with post hoc testing. Only edges with absolute partial correlation ≥0.02 were retained for visualization.

## Results

### Participant characteristics

**FIGURE 1:**
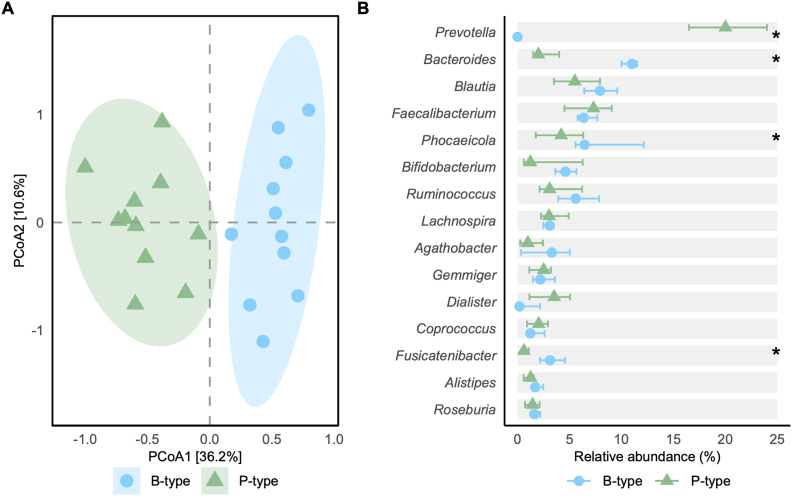
Baseline microbiota composition in B-type and P-type individuals (*n* = 11 per group). (A) Principal coordinate analysis (PCoA) based on Bray–Curtis dissimilarity at the genus level. (B) Relative abundances of the 15 most abundant genera. Statistical differences between enterotypes were assessed using linear mixed models in MaAsLin2. Genera with significant differences are indicated with ∗(*P* < 0.25, Benjamini–Hochberg adjusted). Bars represent the median and IQR. B-type, *Bacteroides*-dominant individuals; P-type, *Prevotella*-dominant individuals.

Of the 64 screened individuals, 31 were eligible (14 P-types and 17 B-types). Seven withdrew preintervention, and 1 B-type discontinued during INU due to gastrointestinal symptoms, resulting in 22 participants (11 per group; Supplemental Figure 2). Compliance was high in both groups (P-types: 99.4%; B-types: 98.1%). At baseline, P-types and B-types were comparable in demographic, clinical, dietary, and stool parameters (Table 1). As expected from the stratification, the groups differed in overall microbiota composition (*R*^2^ = 0.09, *P* < 0.01; Figure 1A) and the relative abundances of *Prevotella* (20% in P-types, absent in B-types) and *Bacteroides* (11% in B-types compared with 2% in P-types; both *q* < 0.01). Beyond these stratification-defining genera, additional baseline differences were observed, including higher relative abundances of *Fusicatenibacter* (2.9% compared with 0.5%, *q* < 0.01) and *Phocaeicola* (6.0% compared with 3.9%, *q* = 0.14) in B-types (Figure 1B, Supplemental Table 2).

**TABLE 1:** Baseline characteristics of study participants.

	Overall^1^ (*n* = 22)	B-types^1^ (*n* = 11)	P-types^1^ (*n* = 11)	*P* value^2^
Age (y)	47(12)	47 (12)	46 (12)	0.70
Sex				>0.90
Male	5 (23%)	3 (27%)	2 (18%)	
Female	17 (77%)	8 (73%)	9 (82%)	
BMI, kg/m^2^	23.5 (2.75)	23.4 (3.20)	23.5 (2.38)	0.60
Waist–hip ratio	0.81 (0.06)	0.81 (0.08)	0.81 (0.05)	0.90
Systolic blood pressure, mmHg	119 (15)	122 (17)	115 (14)	0.20
Diastolic blood pressure, mmHg	74 (11)	76 (11)	73 (10)	0.40
HOMA-IR	1.35 (0.60)	1.41 (0.67)	1.28 (0.55)	0.80
PDI	59.5 (5.00)	61.4 (4.88)	57.7 (4.67)	0.15
hPDI	46.5 (5.17)	45.7 (5.00)	47.2 (5.47)	0.59
uPDI	36.3 (5.74)	38.8 (5.76)	33.7 (4.67)	0.06
Bristol stool chart				0.40
1	2 (9.1%)	1 (9.1%)	1 (9.1%)	
2	2 (9.1%)	1 (9.1%)	1 (9.1%)	
3	6 (27%)	5 (45%)	1 (9.1%)	
4	6 (27%)	3 (27%)	3 (27%)	
5	5 (23%)	1 (9.1%)	4 (36%)	
6	1 (4.5%)	0 (0%)	1 (9.1%)	
7	0 (0%)	0 (0%)	0 (0%)	

Abbreviations: B-type, *Bacteroides*-dominant individuals; hPDI, healthy PDI; PDI, Plant-based Diet Index; P-type, *Prevotella*-dominant individuals; uPDI, unhealthy PDI.

1 Values are represented as *n* (%) or mean (± SD).

2 Differences between baseline characteristics between B-types and P-types were assessed using Fisher’s exact test for categorical variables and the Wilcoxon rank sum test for continuous variables.

### Gastrointestinal symptoms and dietary intake across interventions

In B-types, 6 of 11 participants reported abdominal pain scores ≥3 (at least mild discomfort) after INU, whereas all 11 reported ≤2 (no or minor discomfort) after AX and PLA. In P-types, the occurrence of hard stools was significantly lower after AX and INU compared with PLA (both *P* = 0.04, Supplemental Table 3). Energy and nutrient intake before CIDs did not differ across interventions (Supplemental Table 4).

### Fiber-distinct plasma SCFA/BCFA concentrations and breath H_2_

**FIGURE 2:**
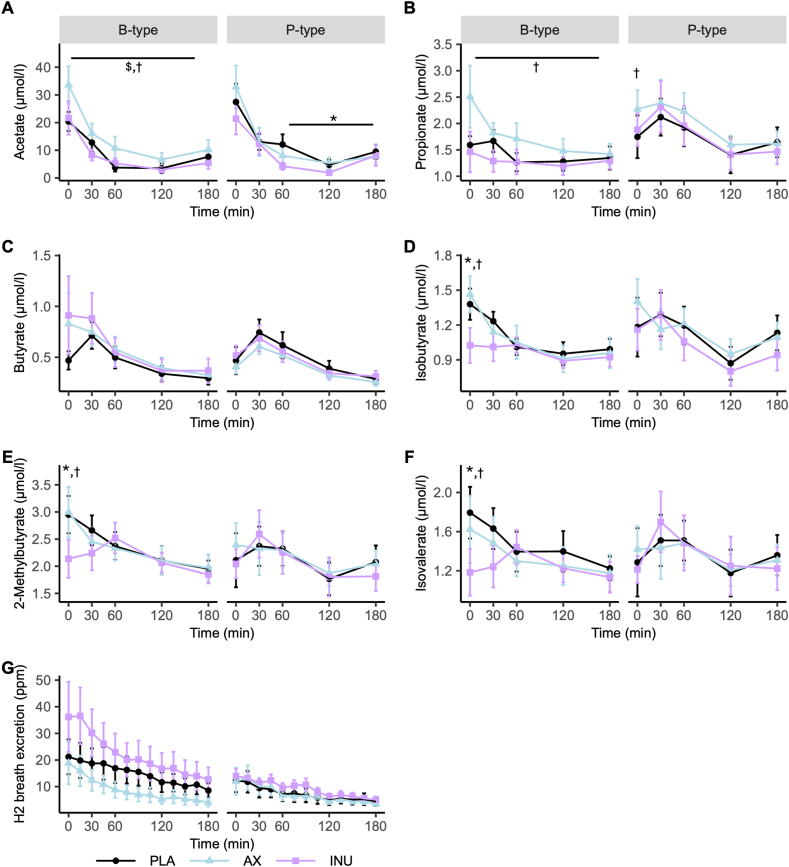
Plasma short-chain and branched-chain fatty acids and breath hydrogen excretion before and after standardized breakfast following PLA, AX, and INU intervention in B-type and P-type individuals (*n* = 11 per group). (A) Plasma acetate, (B) plasma propionate, (C) plasma butyrate, (D) plasma isobutyrate, (E) plasma 2-methylbutyrate, (F) plasma isovalerate, and (G) postprandial breath hydrogen excretion. Data are presented as means ± SEM. Fasting and postprandial differences, based on the AUC, were analyzed using repeated-measures ANOVA. Post hoc pairwise *t* tests were performed with LSD adjustment for *P* values. ∗*P* < 0.05 INU compared with PLA, ^$^*P* < 0.05 AX compared with PLA, ^†^*P* < 0.05 AX compared with INU. ANOVA, analysis of variance; AX, arabinoxylan; B-type, *Bacteroides*-dominant individuals; INU, inulin; LSD, least significant difference; PLA, placebo; P-type, *Prevotella*-dominant individuals.

In B-types, AX significantly increased fasting acetate compared with INU (*P* = 0.01) and PLA (*P* = 0.03). Postprandially, AX raised acetate compared with INU and PLA, particularly during the early phase (AUC_0-60_: AX compared with INU *P* = 0.02; AX compared with PLA *P* = 0.04, Figure 2A). AX increased postprandial propionate compared with INU (*P* = 0.02), with a trend compared with PLA (*P*= 0.07), most pronounced in the first 60 min (AUC_0-60_: AX compared with INU *P* = 0.02; AX compared with PLA *P* = 0.05) (Figure 2B). In both B- and P-types, fasting and postprandial butyrate concetration were not significantly affected by AX, INU or PLA (*P* > 0.05) (Figure 2C). INU significantly reduced fasting concentrations of isobutyrate, 2-methylbutyrate, and isovalerate compared with both AX (all *P* < 0.01) and PLA (all *P* ≤ 0.04) (Figure 2D–F, Supplemental Table 5).

In P-types, fasting propionate concentrations were higher after AX compared with INU (*P* = 0.04) and showed a trend compared with PLA (*P* = 0.05, Figure 2B). Late postprandial acetate was lower after INU compared with PLA (AUC_60-180_ *P* = 0.02) (Figure 2A, Supplemental Table 5).

Breath hydrogen excretion showed no mean differences (ANOVA *P* > 0.10), but variance was higher after INU in B-types (Levene’s test *P* = 0.03). No such effects were observed after AX (*P* = 0.63) or PLA (*P* = 0.15) (Figure 2G, Supplemental Table 5).

### Fiber-specific effects on systemic metabolic markers

In B-types, fasting glucose was higher after INU compared with PLA (*P* = 0.04). Fasting cholesterol was higher after INU than AX (*P* = 0.04), and postprandial cholesterol was higher after INU than PLA (*P* = 0.04). In P-types, no fiber-specific effects were observed for glucose, insulin, cholesterol, or PYY (all *P* > 0.05) (Supplemental Figure 4, Supplemental Table 5).

### Hunger and satiety perceptions altered in P-types after AX supplementation

Appetite ratings varied in P-types. AX reduced postprandial hunger compared with PLA (*P* = 0.01) and INU (*P* = 0.03). AX also lowered late-phase prospective food consumption compared with INU (*P* = 0.04), whereas no significant difference was observed between AX and PLA (*P* = 0.33). Conversely, INU increased prospective food consumption scores compared with PLA (*P* = 0.01). AX also reduced the desire to eat compared with INU (*P* = 0.01), whereas INU increased it compared with PLA (*P* = 0.01). No significant difference was observed between AX and PLA (*P* = 0.18). In B-types, no effects on appetite or satiety were observed (Supplemental Figure 5, Supplemental Table 5).

### Stool frequency and consistency did not differ between fiber intervention

No significant differences in stool consistency or frequency were observed across interventions (all *P* > 0.05, Supplemental Table 6).

### Microbial diversity and composition in response to fiber interventions

**FIGURE 3:**
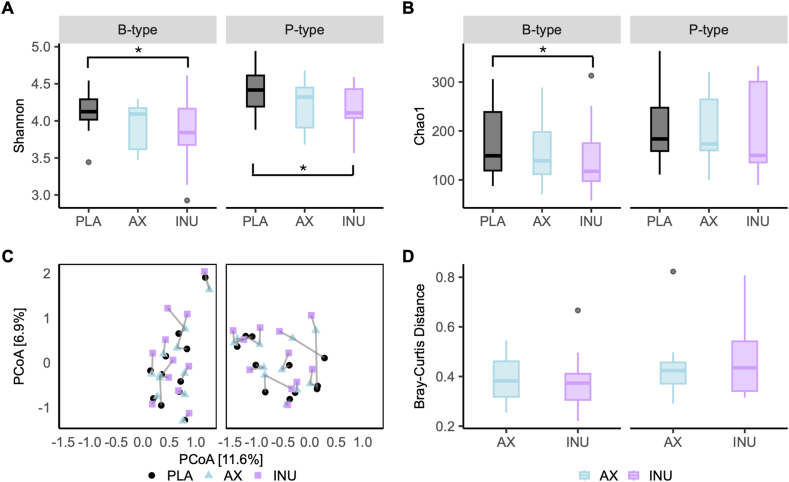
Changes in microbiota diversity and composition after AX, INU, and PLA intervention in B-type and P-type individuals (*n* = 11 per group). (A) Shannon diversity index and (B) Chao1 richness. Differences in alpha diversity between supplementations were analyzed using repeated-measures ANOVA. If *P* < 0.1, post hoc pairwise *t* tests with LSD adjustment were performed. *P* < 0.05 indicates significant differences. (C) Principal coordinate analysis (PCoA) plot based on Bray*–*Curtis distances, with individuals connected by lines. Statistical differences in beta diversity were assessed using linear mixed models (LMMs) to account for the longitudinal study design. (D) Intraindividual microbiota changes compared with PLA. ANOVA, analysis of variance; AX, arabinoxylan; B-type, *Bacteroides*-dominant individuals; INU, inulin; LSD, least significant difference; PLA, placebo; P-type, *Prevotella*-dominant individuals.

INU reduced Shannon diversity compared with PLA in B-types (*P* = 0.02) and P-types (*P* = 0.03). Chao1 richness was also lower after INU than PLA in B-types (*P* = 0.04) but not in P-types. Beta diversity (Bray–Curtis) showed no fiber-induced shifts in overall microbial composition (B-types *P* = 0.78; P-types *P* = 0.45, Figure 3, Supplemental Tables 7 and 8).

### Stability and shifts in group classification after intervention

**FIGURE 4:**
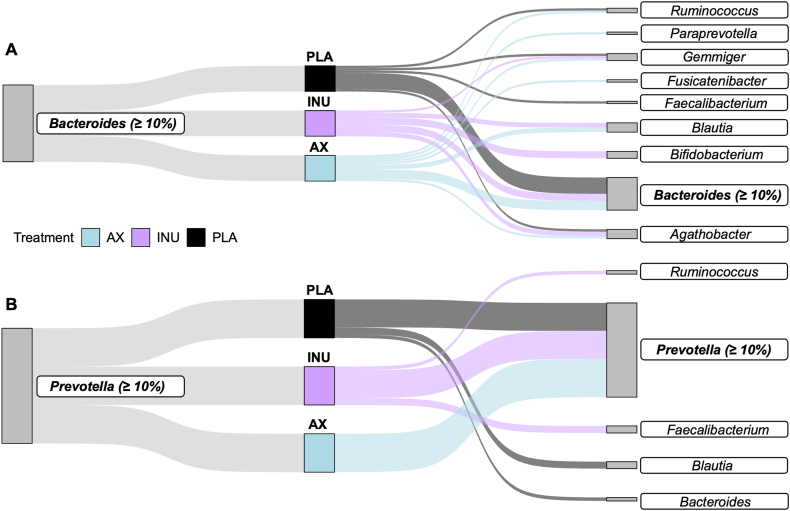
Stability and shifts in microbial group classification after AX, INU, and PLA in B-type (A) and P-type individuals (B) (*n* = 11 per group). Participants were classified as B- or P-types based on a relative abundance of ≥10% *Bacteroides* or *Prevotella* at baseline. The figure illustrates how this classification changed after each intervention. If a participant's relative abundance of *Bacteroides* or *Prevotella* fell <10% postintervention, the most dominant genus at that time point was identified. AX, arabinoxylan; INU, inulin; PLA, placebo.

We evaluated whether participants maintained their baseline classification (≥10% fecal abundance of *Prevotella* or *Bacteroides*) after the intervention and identified the most dominant genera when the thresholds were not met. In P-types, a *Prevotella* ≥10% was maintained in all 11 participants after AX and in 8 participants after both INU and PLA. After INU, 1 participant showed a shift to *Ruminococcus* as the dominant genus and 2 to *Faecalibacterium*. Following the PLA, Bacteroides became dominant in 1 participant and *Blautia* in 2. In B-types, ≥10% *Bacteroides* was observed in 7 participants after PLA, 4 after AX, and 3 after INU. Dominance shifts included *Paraprevotella* and *Fusicatenibacter* (AX), and *Bifidobacterium* (INU). The remaining individuals displayed heterogeneous shifts with no consistent patterns (Figure 4).

### Differentially abundant genera following fiber interventions

**FIGURE 5:**
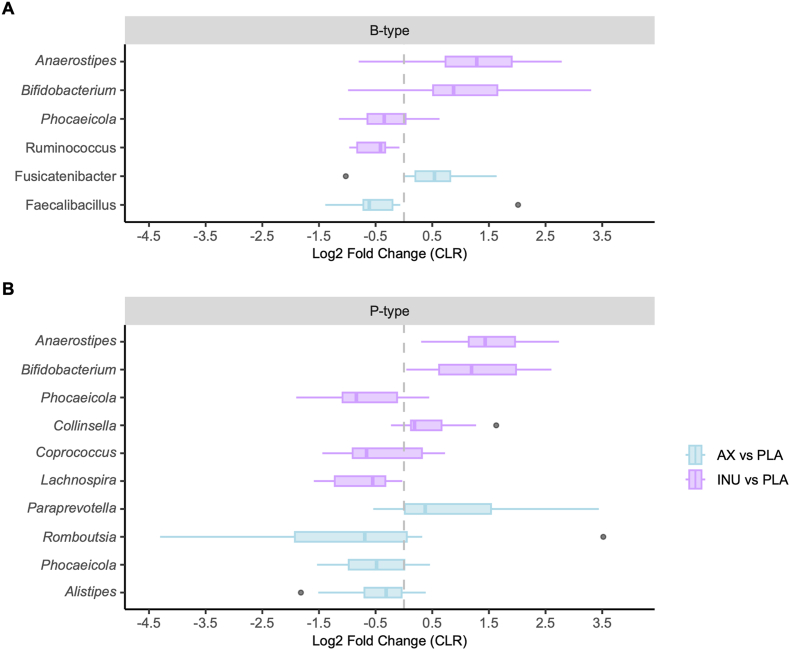
Significantly differential abundant genera across AX and INU compared with PLA in B-type (A) and P-type individuals (B) (*n* = 11 per group). Boxplots depict the log_2_ fold change (after centered log-ratio (CLR) transformation with pseudocount = 1) for taxa that showed significant intervention-dependent differences (*P* < 0.25, Benjamini*–*Hochberg adjusted) in either P-types or B-types, based on MaAsLin2 analysis. AX, arabinoxylan; B-type, *Bacteroides*-dominant individuals; INU, inulin; PLA, placebo; P-type, *Prevotella*-dominant individuals.

Some individual taxa responded to fiber interventions across both groups (Figure 5, Supplemental Table 9). *Anaerostipes* increased after INU compared with PLA in both groups (B-types: *q* = 0.01; P-types: *q* < 0.01), with median abundances rising from 1.61% to 4.92% and 1.47% to 5.41%, respectively. *Bifidobacterium* also increased (B-types: *q* = 0.17; P-types: *q* = 0.03) from 3.03% to 3.77% and 0.83% to 2.57%. *Phocaeicola* decreased after INU (B-types: *q* = 0.18; P-types: *q* = 0.03), from 9.65% to 6.70% and 3.92% to 1.89%. In B-types, *Ruminococcus* decreased after INU (*q* = 0.01, 6.41%–3.65%). AX increased *Fusicatenibacter* (*q* = 0.18; 3.10%–5.20%) and decreased *Faecalibacillus* (*q* = 0.18; 1.15% to 0.51%). In P-types, INU increased *Collinsella* (*q* = 0.07; 1.64%–2.45%) and decreased *Coprococcus* (*q* = 0.17; 2.29%–0.94%) and *Lachnospira* (*q* = 0.17; 1.35%–0.65%). AX supplementation increased *Paraprevotella* (*q* = 0.17; 0.07%–0.22%) and decreased *Alistipes* (*q* = 0.17; 1.49%–0.49%).

### Distinct co-occurrence network structures and centrality patterns across interventions

**FIGURE 6:**
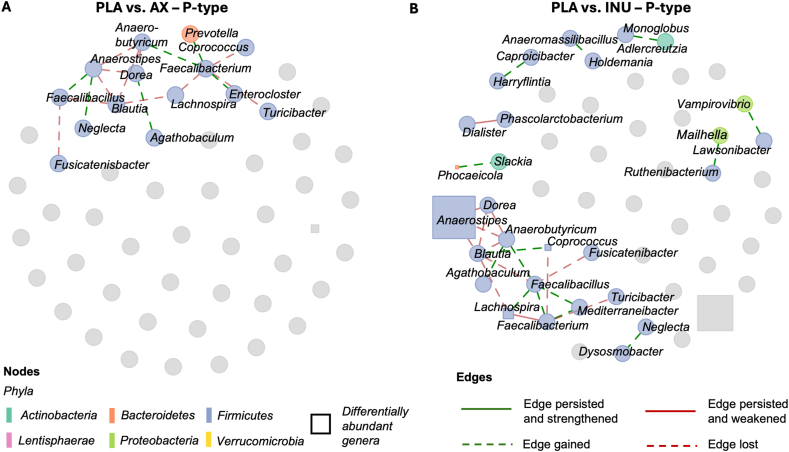
Differential genus-level co-occurrence networks comparing AX (A) and INU (B) to PLA in P-types (*n* = 11). Green dashed edges represent newly emerged correlations; red dashed edges indicate lost correlations relative to PLA. Solid edges represent correlations present in both conditions: green if the correlation was strengthened, red if it was weakened after intervention. Significant differentially abundant genera (identified via MaAsLin2) are shown as squares, sized by median log_2_ fold change in realtive abundances (after centered log-ratio transformation with pseudocount = 1; AX compared with PLA or INU compared with PLA). Only edges with |partial correlation| ≥ 0.02 are shown. AX, arabinoxylan; INU, inulin; PLA, placebo.

In P-types, the PLA network was compact, with a central cluster around *Blautia*, positively linked to *Faecalibacillus*, *Anaerostipes*, *Dorea*, *Lachnospira*, and *Anaerobutyricum* (Supplemental Figure 6A). The strongest correlation was observed between *Lachnospira* and *Faecalibacterium* (edge weight: 0.11). Negative associations included Dialister–*Phascolarctobacterium* and *Faecalibacterium*–*Turicibacter*. After AX, the network became less cohesive, resulting in significantly reduced closeness centrality after AX compared with PLA (*P* = 0.01) and INU (*P* < 0.01), indicating lower connectivity (Supplemental Table 10). *Blautia* retained only its link to *Dorea* (edge weight: 0.06), and a new positive association emerged between *Faecalibacterium* and *Prevotella*. *Anaerostipes*, *Faecalibacillus*, and *Neglecta* formed a new triad (Supplemental Figure 6B, see also Figure 6A for differential edges). INU induced a different structure with a new central hub around *Faecalibacterium*, positively associated with *Mediterraneibacter*, *Lachnospira*, *Anaerobutyricum*, *Faecalibacillus*, and *Agathobaculum*. The strongest link was *Faecalibacillus*–*Faecalibacterium* (edge weight: 0.09), whereas the *Dialister*–*Phascolarctobacterium* edge weakened (edge weight: –0.04) **(**Supplemental Figure 6C and B).

**FIGURE 7:**
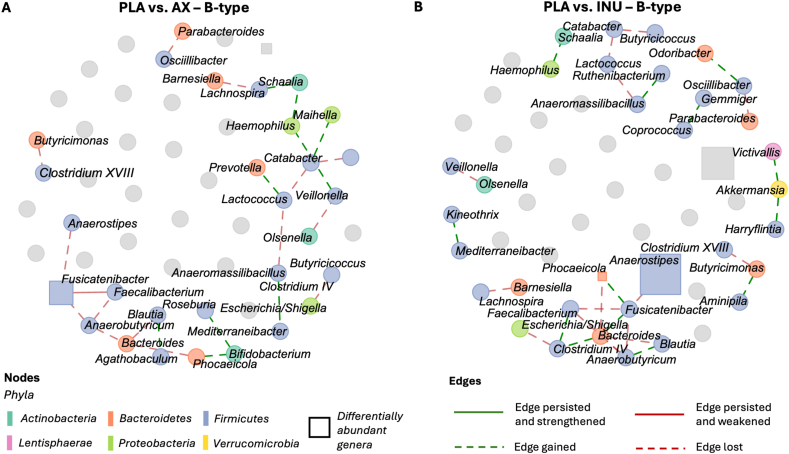
Differential genus-level co-occurrence networks comparing AX (A) and INU (B) to PLA in B-types (*n* = 11). Green dashed edges represent newly emerged correlations; red dashed edges indicate lost correlations relative to PLA. Solid edges represent correlations present in both conditions: green if the correlation was strengthened, red if it was weakened after intervention. Significant differentially abundant genera (identified via MaAsLin2) are shown as squares, sized by median log_2_ fold change in relative abundances (after centered log-ratio (CLR) transformation with pseudocount = 1; AX compared with PLA or INU compared with PLA). Only edges with |partial correlation| ≥ 0.02 are shown. AX, arabinoxylan; INU, inulin; PLA, placebo.

In B-types, the PLA network appeared more fragmented, with several small subnetworks (Supplemental Figure 7A). One cluster included *Fusicatenibacter*, *Anaerostipes*, *Anaerobutyricum*, and *Faecalibacterium*, which connected to a second group comprising *Bacteroides*, *Blautia*, and *Phocaeicola*. The strongest edge linked *Butyricimonas* and *Clostridium_XVIII* (edge weight: 0.10), whereas a separate strong correlation was observed between *Parabacteroides* and *Oscillibacter* (edge weight: 0.09). After AX, new connections emerged, such as *Lactococcus–Prevotella*, *Agathobaculum–Blautia*, and *Bifidobacterium–Phocaeicola*. The *Fusicatenibacter–Faecalibacterium* connection persisted with reduced strength (edge weight: 0.02) (Supplemental Figure 7B; Figure 7A). Betweenness centrality was higher after AX than PLA (*P* = 0.02), suggesting that nodes served more often as intermediaries within the network (Supplemental Table 11). INU supplementation resulted in a markedly different network in B-types (Supplemental Figure 7C; Figure 7B), with no connections retained from PLA. *Bacteroides* regained connectivity through a new link with *Fusicatenibacter*, which also connected to *Phocaeicola*. The strongest edge was between *Hemophilus* and *Schaalia* (edge weight: 0.11). Full adjacency matrices are available in Supplemental Tables 11 and 12.

### No fiber-specific interactions in SCFA/BCFA–microbiota associations

**FIGURE 8:**
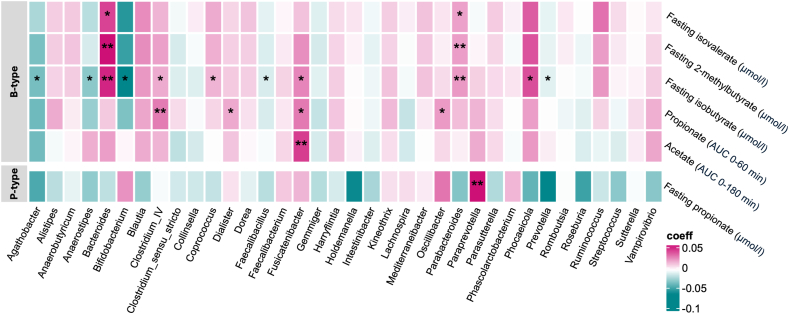
Associations between microbial genera and short-chain and branched-chain fatty acid concentrations within B- and P-types, pooled across all 3 treatment arms (AX, INU, and PLA) (*n* = 11 per group). Heatmap colors represent coefficients from linear mixed models computed in MaAsLin2. Genera were filtered for ≥ 10% prevalence and ≥ 1% relative abundance. Statistical significance was determined using the Benjamini–Hochberg method with a target false discovery rate of *q* < 0.25. ∗*q* < 0.25, ∗∗*q* < 0.1. AX, arabinoxylan; B-type, *Bacteroides*-dominant individuals; INU, inulin; PLA, placebo; P-type, *Prevotella*-dominant individuals.

To investigate links between microbial taxa and SCFA/BCFA concentrations, we first pooled data across all interventions and accounted for repeated measures. In B-types, postprandial acetate (AUC_0_–_180_) correlated positively with *Fusicatenibacter* (*q* < 0.01). Early postprandial propionate (AUC_0_–_60_) was associated with *Clostridium_IV* (*q* = 0.02), *Oscillibacter* (*q* = 0.13), *Dialister* (*q* = 0.16), and *Fusicatenibacter* (*q* = 0.16). Fasting isobutyrate was positively linked to *Parabacteroides*, *Bacteroides*, *Phocaeicola*, *Fusicatenibacter*, *Clostridium_IV*, and *Coprococcus* (all *q* < 0.01, Supplemental Table 13) and negatively to *Agathobacter*, *Anaerostipes*, *Bifidobacterium*, *Faecalibacillus*, and *Prevotella* (all *q* = 0.19). Fasting 2-methylbutyrate and isovalerate were positively associated with *Bacteroides* (*q* = 0.02 and *q* = 0.23) and *Parabacteroides* (*q* = 0.08 and *q* = 0.23). In P-types, fasting propionate correlated with *Paraprevotella* (*q* = 0.02) (Figure 8). In a subsequent step, we tested whether these associations were fiber-specific by adding fiber × genus interaction terms to linear mixed models. None of the interaction terms reached statistical significance (Supplemental Figures 8–14).

## Discussion

In this single-blinded, placebo-controlled, randomized crossover study, we investigated whether healthy individuals with either a *Prevotella*- or *Bacteroides*-dominated microbiota exhibit distinct SCFA responses after 1 wk of AX or INU intake compared with a placebo. In B-types, AX increased fasting plasma concentrations of propionate and acetate. In contrast, INU did not affect plasma SCFA concentrations in either group but consistently decreased fasting BCFAs, including isobutyrate, 2-methylbutyrate, and isovalerate, in B-types. In P-types, AX also increased fasting plasma propionate. Microbiota composition shifted in response to both fibers. INU induced similar taxonomic changes in both groups, characterized by increased *Anaerostipes* and *Bifidobacterium*, decreased *Phocaeicola*, along with reduced microbial diversity. AX effects were more group specific, increasing *Fusicatenibacter* in B-types and *Paraprevotella* in P-types. Distinct changes in microbial co-occurrence networks accompanied these compositional shifts. AX reduced closeness centrality in P-types and increased betweenness centrality in B-types, indicating reduced network connectivity in P-types and greater node influence in B-types. Postprandial H_2_ excretion varied widely after INU in B-types, a pattern not observed in P-types. Postprandial glucose, insulin, and PYY concentrations remained unchanged. However, INU led to higher fasting glucose and total cholesterol than placebo. AX reduced subjective appetite ratings in P-types.

Several post hoc analyses suggested that individuals with a high *P-/B* ratio may benefit more from fiber-rich diets, particularly for weight management [18,19,39,40]. In vitro fermentation models further demonstrated that AX is more efficiently fermented in *Prevotella*-rich microbiota, resulting in higher propionate production compared with *Bacteroides*-dominated microbiota [29]. Although both our study and Chen et al. [29] used corn bran-derived AX, their material was extracted using alkaline hydrogen peroxide, potentially altering microbial accessibility. In contrast, we used a minimally processed, high-molecular-weight long-chain AX. In our study, 7 d of AX intake increased plasma propionate in both groups. Consistent with these findings, Nguyen et al. [42] proposed that *Bacteroides* species act as primary degraders of long-chain AX due to their xylanolytic CAZyme repertoire. Although *Prevotella copri* may also initiate degradation, other *Prevotella* species have been described as secondary metabolite utilizers, converting intermediates such as succinate to propionate via methylmalonyl-CoA [41]. *Paraprevotella* increased significantly after AX in P-types and correlated positively with fasting plasma propionate. As a known succinate converter [43,44], this suggests a potential role in propionate biosynthesis [23]. However, its relative abundance <1% raises questions about its contribution to systemic propionate. To explore broader mechanisms, we examined microbial co-occurrence networks. *Prevotella* formed new positive edges with other taxa in both groups after AX intervention. Although dominant in P-types and low-abundant in B-types, *Prevotella* appeared as a connected node only after AX supplementation. Such positive connections are often interpreted as indicators of metabolic cooperation or substrate-driven cross-feeding [45]. Notably, *Prevotella* was linked to *Faecalibacterium* in P-types and *Lactococcus* in B-types, both genera involved in the production of SCFAs [46,47]. These links may indicate lactate or succinate exchange, supporting *Prevotella’s role* in AX-induced propionate formation even at low abundance.

Despite increased plasma propionate following AX, postprandial glucose, insulin, and PYY remained unchanged. Propionate has been shown to contribute to glucose metabolism and appetite regulation, primarily through hepatic gluconeogenesis [24], and stimulation of gut hormone release of GLP-1 and PYY [48]. However, such effects may require prolonged exposure or are more evident in metabolically impaired individuals, where homeostatic mechanisms are less robust [49]. Interestingly, although PYY was unchanged, AX reduced subjective appetite ratings in P-types, possibly via alternative pathways like GLP-1, delayed gastric emptying, or SCFA-responsive neural signaling [23].

AX also increased plasma acetate concentrations in B-types. *Fusicatenibacter*, more abundant in B-types at baseline, increased further after AX. Given its positive correlation with postprandial acetate, *Fusicatenibacter* may contribute to acetate production, although its precise functional role remains incompletely defined. *F. saccharivorans* is known to produce acetate from saccharolytic fermentation [50] and lacks pathways for both butyrate and propionate formation [51], suggesting a mechanistic link between its enrichment and the systemic rise in acetate. Network analysis revealed additional taxa potentially involved in acetate production, such as *Bifidobacterium* and *Phocaeicola*, which are both known to produce acetate via pyruvate decarboxylation to acetyl-CoA [52]. In addition, AX induced a positive connection between *Blautia* and *Agathobaculum*. *Blautia* possesses the Wood–Ljungdahl pathway for acetogenesis [53], whereas *Agathobaculum* produces acetate during anaerobic fermentation [54]. Their co-occurrence may indicate ecologic overlap or parallel roles in acetate production. These findings suggest that acetate increases in B-types may result from both direct acetogenesis and cross-feeding. Given the link between increased acetate and insulin sensitivity, lipid oxidation, and gut hormone secretion [25], the increase in B-type acetate may represent a favorable metabolic adaptation, although further investigation is needed.

Contrary to our hypothesis, INU did not yield distinct metabolic benefits in B-types despite anticipated competition between *Bacteroides* and *Bifidobacterium* [30]. Although we expected *Bacteroides* suppression via bifidogenic fermentation, *Bacteroides* remained stable, whereas *Phocaeicola* decreased in both groups. This suggests that INU-adapted taxa, such as *Bifidobacterium* and *Anaerostipes*, may have competitively suppressed *Phocaeicola* instead. INU selectively promotes these taxa [11,55], and their enrichment coincided with reduced alpha diversity, consistent with previous short-term INU interventions [56]. Rather than indicating dysbiosis [57], this likely reflects transient overgrowth during niche restructuring. The selective decline in *Phocaeicola* aligns with recent suggestions to treat *Bacteroides* and *Phocaeicola* as distinct but co-occurring genera [58]. Our network analysis supports this interpretation; a positive co-occurrence between *Phocaeicola* and *Bacteroides* was present after PLA but was lost after INU intervention, suggesting disruption of their ecologic linkage. Notably, *Bifidobacterium* and *Anaerostipes* did not form network edges post-INU, indicating expansion independent of network integration.

Although INU supplementation increased *Bifidobacterium* and *Anaerostipes* in both groups, plasma SCFA concentrations remained unchanged. One explanation is microbial cross-feeding. *Bifidobacterium* primarily produces lactate and acetate, which *Anaerostipes* can convert into butyrate [59,60]. However, butyrate is rapidly utilized by colonocytes and rarely appears in systemic circulation [24]. In addition, long-chain INU is fermented more slowly in distal colon regions [61], where SCFA uptake may prevent systemic increases. Still, INU reduced plasma BCFAs in B-types. Although previous studies have reported reductions in fecal BCFA, to our knowledge, this is the first study demonstrating a decrease in plasma BCFA after INU supplementation, specifically in B-type individuals. BCFAs (isobutyrate, isovalerate, and 2-methylbutyrate) arise from branched-chain amino acid fermentation and are linked to adverse colonic and metabolic effects [62]. *Bacteroides*-dominant microbiomes have been shown to possess a high capacity for protein fermentation but may shift toward carbohydrate metabolism when fermentable fiber is available [21]. In our study, this shift coincided with the reduction of *Phocaeicola*. This genus includes species such as *P. vulgatus* (formerly *Bacteroides vulgatus*), which is also associated with proteolytic activity [63]. Thus, INU may reduce proteolytic byproducts in B-types via metabolic adaptation and compositional changes. Although reduced BCFAs are generally considered beneficial, their clinical relevance in healthy individuals remains to be determined.

Microbial hydrogen metabolism may also shape individual fiber responses. H_2_ is a key fermentation byproduct reflecting gut fermentation activity [64]. In our study, P-types showed stable H_2_ excretion across interventions, possibly due to efficient H_2_ incorporation via *Prevotella*-driven fumarate reduction to succinate [65,66]. In contrast, B-types exhibited high interindividual variability in H_2_ excretion after INU. One potential contributor could be the expansion of *Anaerostipes*, a butyrate-producing genus that releases H_2_ as a byproduct of fermentation via the acetyl-CoA pathway [64]. This increase in H_2_ production may have led to greater luminal H_2_ in some individuals, especially if hydrogenotrophic activity varied. However, no consistent shifts in methanogens, sulfate reducers, or acetogens were observed, leaving the drivers of this variability unresolved. Increased H_2_ may explain bloating and discomfort in some B-types, consistent with luminal H_2_ effects [67].

Despite fiber’s known benefits for cholesterol and glucose homeostasis [4], we observed increased fasting glucose and cholesterol concentrations in B-types following INU. Because concentrations of glucose and cholesterol were within the healthy physiological range, the clinical relevance of this observation remains uncertain.

Taken together, our findings suggest that P-types maintain a more stable microbial and metabolic profile, whereas B-types exhibit greater plasticity in response to fiber intervention. Participants were classified based on a relative abundance of ≥10% *Prevotella* or *Bacteroides* at baseline. P-types largely retained their classification throughout the study, indicating a robust microbial signature, whereas B-types showed greater shifts, particularly after INU. These differences were also reflected in microbial network structures. Co-occurrence networks in P-types formed more cohesive clusters, whereas B-type networks, with loosely connected subnetworks, appeared more fragmented. This supports the interpretation that P-types represent more resilient ecosystems, whereas B-types may undergo broader functional reorganization in response to dietary change. This pattern aligns with previous research suggesting that *Prevotella*-dominated microbiomes are less affected by dietary changes, whereas *Bacteroides*-dominated communities are more responsive to dietary shifts [21]. Future studies should evaluate whether more flexible classification models improve the predictive power of microbiome-based dietary interventions.

This study has several limitations. First, the single-blinded design made it not entirely feasible to ensure complete participant blinding due to the distinct appearance of the AX supplement and the lower weight of the PLA dose. Although intervention identities were not disclosed, we cannot exclude the possibility that some participants may have inferred their allocation, which could have influenced their subjective outcomes. Furthermore, the modest sample size may have limited statistical power to detect subtle microbiota–diet interactions, especially for SCFAs and taxonomic shifts. In addition, this was a proof-of-concept study, and the findings should therefore be interpreted as preliminary and hypothesis generating rather than confirmatory. Additionally, although we categorized participants based on their *Bacteroides* or *Prevotella* abundance, not all individuals exhibited a strong dominance of either group. Future studies could consider alternative typing approaches such as functional profiling or metabolic phenotypes. Another limitation is the timing of metabolic assessments. SCFA and H_2_ production in P-types may have been underestimated, as measurements were conducted ∼12 h after the last fiber intake. Most observed effects on plasma markers occurred in the fasting or early postprandial state, highlighting the importance of sampling time. Future research should consider shorter sampling intervals or real-time tracking tools (e.g., breath hydrogen or continuous metabolomics) to better capture dynamic responses.

Another potential limitation is the presence of residual confounding factors that may have influenced microbiota composition before and after the intervention. Although 24-h dietary recalls were collected to account for habitual dietary intake and fiber consumption, participants were instructed to maintain their usual physical activity patterns and report any medication use, unmeasured lifestyle or dietary variations cannot be entirely excluded. Moreover, diurnal fluctuations, seasonal influences, and minor deviations from the instructed regimen could still have contributed to variability. However, the crossover design of the study inherently minimized interindividual variability, thereby reducing the potential impact of such factors.

Moreover, despite the enrichment of known butyrate-producing taxa such as *Anaerostipes*, neither intervention led to a measurable increase in plasma butyrate in either microbiota type. As discussed above, this likely reflects rapid colonic utilization and the limited systemic availability of butyrate, which is rarely detected in peripheral circulation despite active microbial production [24].

In conclusion, although AX and INU may serve as complementary strategies for enhancing SCFA production and suppressing proteolytic fermentation in B-types, the metabolic benefits for P-types remain less evident. This highlights the need for more refined microbiome-targeted dietary strategies, incorporating functional microbiome profiling to improve precision in dietary recommendations.

## Author contributions

The authors’ responsibilities were as follows – MM, AH: designed research; MB, SW, FGB, MV, MM: conducted research; MB, MM: analyzed data and wrote the paper; SBB, AH, MM: critically reviewed the paper; MM, AH: primary responsibility for final content; SBB: funded research; and all authors: read and approved the final manuscript.

## Data availability

Data described in the manuscript, code book, and analytic code will be made available on request. The raw 16s rRNA gene sequencing data are publicly available in the European Nucleotide Archive under project ID PRJEB92105.

## Funding

MB was supported by the CARLA Talent Academy – Health and Living of Osnabrück University of Applied Sciences. MM was supported by the Joachim Herz Foundation (Add-on Fellowship for interdisciplinary Life Sciences).

## Conflict of interest

MM reports financial support was provided by Joachim Herz Foundation. All other authors report no conflicts of interest.
