# Supplementary material for: Microbiota-Dependent Fiber Responses: A Proof-of-Concept Study on Short-Chain Fatty Acid Production in Prevotella- and Bacteroides-Dominated Healthy Individuals
Source: J Nutr. 2025 Sep 2;155(11):3809–22. doi: 10.1016/j.tjnut.2025.08.034 (PMC12799433; doi:10.1016/j.tjnut.2025.08.034)
Supplement: Multimedia component 1 [file mmc1.docx]

# **Title**

Microbiota-Dependent Fiber Responses: A Proof-of-Concept Study on Short-chain Fatty Acid Production in *Prevotella*- and *Bacteroides*-Dominated Healthy Individuals

**First author**

Madeline Bartsch, [bartsch@foh.uni-hannover.de](mailto:bartsch@foh.uni-hannover.de)

**Supplementary Methods – Study Participants**

Blood samples were collected via venipuncture using Safety-Multifly® needles (Sarstedt, Nümbrecht, Germany), stored at 5°C, and transferred to an external laboratory (LADR GmbH, Hannover, Germany) on the same day for fasting glucose and insulin analysis.

Supplementary Methods – Study Intervention, Study Products and Specifications

- Inulin (INU): Frutafit TEX!, Sensus B.V., Roosendaal, The Netherlands
- Arabinoxylan (AX): AgriFiber BFG, AgriFiber Solutions LLC, Illinois, USA
- Placebo (PLA): Glucidex IT 12, Roquette Frères, Lestrem, France Preparation

*Instructions*

AX was dissolved in 200 mL of cold water using a shaker. INU and PLA were stirred into 200 mL of hot water.

**Supplementary Methods – Plasma Biomarker Analysis**

Blood samples were drawn into pre-chilled tubes and centrifuged at 2000 g for 15 minutes at 4°C, and aliquoted serum and plasma samples were immediately stored at -80°C for later analysis. Glucose, insulin, and cholesterol were analyzed in a single batch at an external laboratory (LADR GmbH, Hannover, Germany). SCFA and BCFA were quantified at Maastricht University (Department of Human Biology). Insulin and cholesterol were measured using 7.5 ml serum gel tubes (Sarstedt, Nümbrecht, Germany) with an electrochemiluminescence immunoassay on a Cobas 801e analyzer (Roche Diagnostics GmbH, Mannheim, Germany). Glucose was measured in 3.1 ml GlucoEXACT tubes (Sarstedt, Nümbrecht, Germany) using a photometric method (Beckman Coulter GmbH, Krefeld, Germany). PYY was measured in fasting samples and at 120 minutes postprandially using 2 ml aprotinin K3EDTA tubes (Becton Dickinson, New Jersey, USA) with 20 μl dipeptidyl peptidase-IV inhibitor (Millipore, Darmstadt, Germany) and analyzed using an ELISA kit (antibodies-online GmbH, Aachen, Germany). Blood samples for SCFA and BCFA analysis were collected in lithium heparin tubes (Sarstedt, Nümbrecht, Germany). SCFA and BCFA quantification was performed via liquid chromatography-mass spectrometry (LC-MS) following derivatization with 3-nitrophenylhydrazine (3NPH) as previously described (1). Short, plasma samples (150 µl) were mixed with an internal standard (2-methyl butyric acid, 20 µM), derivatized with 3NPH (160 mM) and 1-ethyl-3-(3-dimethylaminopropyl) carbodiimide hydrochloride (EDC, 200 mM), incubated at 50°C for 30 minutes, extracted with diethyl ether, dried under nitrogen, reconstituted in 60 µl buffer. Analyzed on a LC-MS with a C18 Hypersil Gold column (Thermo Scientific) using a methanol-water-mobile phase coupled on-line to a Q Exactive (Thermo Scientific). Quantification was performed in negative electrospray ionization mode (ESI-) using targeted Selected Ion Monitoring (tSIM). Peak areas were integrated and concentrations calculated by calibration curves using the Thermo Scientific Xcalibur 4.4 software (Thermo Scientific).

**Supplementary Methods – Fecal sampling and microbiota composition**

Participants self-collected stool samples at home using fecal collection kits provided by Süsse Labortechnik (Gudensberg, Germany). Collection tubes were pre-filled with 3.5 ml of RNASepar stabilizer (Biosepar GmbH, Simbach, Germany). Upon delivery to the university, samples were immediately stored at -80°C. DNA was extracted using the ZymoBIOMICS Miniprep Kit (ZYMO, USA), followed by amplification of the V3/V4 region of the 16S rRNA gene. Sequencing was performed on an Illumina MiSeq platform (2 × 300 bp) following previously established protocols (2). Sequence data were processed using the DADA2 pipeline (v1.20), and taxonomy was assigned based on the RDP database (v18). Chimera sequences were removed, and only sequences with at least five counts and phylum-level annotations were included in downstream analyses.

**Supplementary Methods – Statistical Analysis of Clinical Parameters**

Model assumptions for repeated-measures ANOVA were assessed using residual plots, ShapiroWilk tests for normality (shapiro_test()), Levene’s test for homogeneity of variance (levene_test()), and quantile–quantile plots (ggqqplot()). Outliers were identified with identify_outliers() (all functions from rstatix, v0.7.2). Variables not meeting normality assumptions were log-transformed.

**Supplementary Methods – Statistical Analysis of Microbiome Data**

Microbiota data were processed and analyzed in R (version 4.4.1) using the mia package (v1.13.34) (3). Alpha diversity (Shannon index, Chao1 richness) was calculated on rarefied count data (5000 reads/sample), generated using rarefyAssay(), and analyzed with repeated-measures ANOVA. Pairwise t-tests were conducted for post-hoc comparisons under the same conditions as clinical parameters. Beta diversity was assessed using Bray–Curtis dissimilarities and visualized via PCoA using the vegdist() and ordinate() functions in phyloseq (v1.46.0). Baseline beta diversity differences between microbial groups were tested with PERMANOVA using adonis2() from the vegan package (v2.6-4). For longitudinal changes across interventions, the generate_beta_trend_test_long() function from the MicrobiomeStat package (v1.2.1) was applied, fitting linear mixed-effects models with participant ID as random effect and supplementation as time-dependent fixed factor (4). Differentially abundant genera were identified using MaAsLin2, applying multivariable linear models to examine associations between microbial features and clinical metadata (v1.19.0). Input data were filtered to include genera with ≥10% prevalence and ≥1% relative abundance. The models used centered log-ratio (CLR) transformed ASV abundances, with participant ID as a random effect and intervention as fixed effect (reference = PLA). Multiple testing correction was performed using the Benjamini–Hochberg method; results with q < 0.25 were considered significant, as recommended by the developers (5). Log₂ fold changes were calculated and visualized using ggplot2 (v3.5.1). Associations between genera and plasma SCFA concentrations were analyzed using MaAsLin2, specifying SCFA concentrations as fixed effect and participant ID as random effect. To explore intervention-specific associations, an interaction term between SCFA and supplementation was introduced in a linear mixed-effects model (LMM). All PCoA visualizations were created with the microViz package (v0.12.3).

**Supplementary Methods – Microbial co-occurrence network inference**

Microbial co-occurrence networks were inferred using the LIMON package (version 0.1.0), specifically designed for longitudinal microbiome studies. Genus-level count data were first filtered for taxa with ≥20% prevalence across all samples and CSS-normalized. Treatment-specific networks (PLA, AX, INU) were computed using graphical LASSO applied to the residuals of a linear mixed-effects model, including subject ID as a random effect and compliance as a covariate. Model selection was based on the bootstrap stability criterion (bstars). Sparsity parameters (lambda) were optimized separately for each condition but required to be lambda-equivalent for valid subtraction in differential network construction. Differential networks were generated by matrix subtraction (AX–PLA, INU–PLA) after confirming comparable sparsity structures. Only edges with absolute partial correlation ≥ 0.02 were included. Network layouts were visualized using the Fruchterman–Reingold algorithm. In addition to group-level networks, subject-specific networks were inferred to assess topological changes in microbial interactions. Centrality metrics (degree, closeness, betweenness, eigenvector centrality) were calculated for each node and compared across interventions using repeated-measures ANOVA, followed by post-hoc comparisons with effect sizes. All visualizations were generated using ggraph (v2.1.0) and igraph (v1.6.0).

**References**

1. van Deuren T, Smolders L, Hartog A, Bouwman FG, Holst JJ, Venema K, et al. Butyrate and hexanoate-enriched triglycerides increase postprandrial systemic butyrate and hexanoate in men with overweight/obesity: A double-blind placebo-controlled randomized crossover trial. Front Nutr. 2022;9:1066950.

2. Kircher B, Woltemate S, Gutzki F, Schlüter D, Geffers R, Bähre H, et al. Predicting butyrate- and propionate-forming bacteria of gut microbiota from sequencing data. Gut Microbes. 2022;14(1):2149019.

3. Borman T, Ernst FGM, Shetty SA, Lahti L. mia: Microbiome analysis. 2025.

4. Zhou H, He K, Chen J, Zhang X. LinDA: linear models for differential abundance analysis of microbiome compositional data. Genome Biology. 2022;23(1):1-23.

5. Mallick H, Rahnavard A, McIver LJ. MaAsLin 2: Multivariable Association in Populationscale Meta-omics Studies. 2020.

**Supplementary Table 1A. Composition of the standardized low fiber breakfast.**

| **Food** | **Amount** |
| --- | --- |
| Orange juice | 200 g |
| Toast | 100 g |
| Cream cheese | 33.4 g |
| Strawberry jam | 15.0 g |
| Gouda cheese | 37.0 g |

Abbreviations: g, grams.

**Supplementary Table 1B. Nutritional breakdown of the standardized low fiber breakfast.**

| **Nutrient** | **Amount** |
| --- | --- |
| Energy (kcal) | 570 |
| Carbohydrates (g) | 64.4 |
| Total fat (g) | 25.1 |
| Protein (g) | 19.9 |
| Fiber (g) | 3.42 |

The macronutrient composition of the standardized breakfast was calculated using the myfood24 nutrition software. Abbreviations: Kcal, kilocalories; g, grams.

**Supplementary Table 2. Differential abundant genera at baseline between B- and P-types.**

| **Feature** | **Metadata** | **Value** | **Coef** | **StdErr** | **N** | **N.not.0** | **p-value** | **q-value** |
| --- | --- | --- | --- | --- | --- | --- | --- | --- |
| *Prevotella* | Group | Prevotella | 0.48 | 0.04 | 22.0 | 15.0 | <0.001 | <0.001 |
| *Bacteroides* | Group | Prevotella | -0.20 | 0.02 | 22.0 | 22.0 | <0.001 | <0.001 |
| *Fusicatenibacter* | Group | Prevotella | -0.10 | 0.02 | 22.0 | 22.0 | <0.001 | <0.001 |
| *Phocaeicola* | Group | Prevotella | -0.10 | 0.04 | 22.0 | 22.0 | 0.01 | 0.14 |

Genera exhibiting differential abundance between enterotypes at baseline were identified using linear mixed models in MaAsLin2. Only genera with a prevalence of ≥10% and a relative abundance of ≥1% were included in the analysis. Statistical significance was determined using the Benjamini–Hochberg method for multiple comparison adjustment, with a significance threshold set at q < 0.25. Abbreviations: Coef, coefficient; StdErr, standard error; N, number of samples; N.not.0, number of non-zero observations.

**Supplementary Table 3. Summary of adverse effects by treatment group.**

| **B-types** | | | | | | | | | | | | | | |
| --- | --- | --- | --- | --- | --- | --- | --- | --- | --- | --- | --- | --- | --- | --- |
|  |  |  |  | |  | |  |  |  | *Pairwise Eisinger post hoc test* | | | |  |
|  | ****AX**  N = 11** | ****INU**  N = 11** | ****PLA**  N = 11** | | Statistic | | df | p_value | EffectSize | Comparison | Posthoc_pval | Z_value | effsize_r |  |
| **Abdominal pain** |  |  |  | | 8.60 | | 2.00 | 0.01 | 0.39 |  |  |  |  |  |
| 1 | 10 (91%) | 3 (27%) | 9 (82%) | |  | |  |  | 0.69 | INU vs AX | 0.02 | 2.27 | 0.69 |  |
| 2 | 1 (9.1%) | 2 (18%) | 2 (18%) | |  | |  |  | 0.09 | PLA vs AX | 0.75 | 0.32 | 0.09 |  |
| 3 | 0 (0%) | 1 (9.1%) | 0 (0%) | |  | |  |  | 0.55 | PLA vs INU | 0.07 | 1.82 | 0.55 |  |
| 4 | 0 (0%) | 1 (9.1%) | 0 (0%) | |  | |  |  |  |  |  |  |  |  |
| 5 | 0 (0%) | 2 (18%) | 0 (0%) | |  | |  |  |  |  |  |  |  |  |
| 6 | 0 (0%) | 2 (18%) | 0 (0%) | |  | |  |  |  |  |  |  |  |  |
| 7 | 0 (0%) | 0 (0%) | 0 (0%) | |  | |  |  |  |  |  |  |  |  |
| **Chest pain** |  |  |  | | 2.92 | | 2.00 | 0.23 | 0.13 |  |  |  |  |  |
| 1 | 8 (73%) | 7 (64%) | 10 (91%) | |  | |  |  |  |  |  |  |  |  |
| 2 | 2 (18%) | 3 (27%) | 1 (9.1%) | |  | |  |  |  |  |  |  |  |  |
| 3 | 0 (0%) | 1 (9.1%) | 0 (0%) | |  | |  |  |  |  |  |  |  |  |
| 4 | 1 (9.1%) | 0 (0%) | 0 (0%) | |  | |  |  |  |  |  |  |  |  |
| 5 | 0 (0%) | 0 (0%) | 0 (0%) | |  | |  |  |  |  |  |  |  |  |
| 6 | 0 (0%) | 0 (0%) | 0 (0%) | |  | |  |  |  |  |  |  |  |  |
| 7 | 0 (0%) | 0 (0%) | 0 (0%) | |  | |  |  |  |  |  |  |  |  |
| **Heartburn** |  |  |  | | 4.00 | | 2.00 | 0.14 | 0.18 |  |  |  |  |  |
| 1 | 9 (82%) | 11 (100%) | 11 (100%) | |  | |  |  |  |  |  |  |  |  |
| 2 | 2 (18%) | 0 (0%) | 0 (0%) | |  | |  |  |  |  |  |  |  |  |
| 3 | 0 (0%) | 0 (0%) | 0 (0%) | |  | |  |  |  |  |  |  |  |  |
| 4 | 0 (0%) | 0 (0%) | 0 (0%) | |  | |  |  |  |  |  |  |  |  |
| 5 | 0 (0%) | 0 (0%) | 0 (0%) | |  | |  |  |  |  |  |  |  |  |
| 6 | 0 (0%) | 0 (0%) | 0 (0%) | |  | |  |  |  |  |  |  |  |  |
| 7 | 0 (0%) | 0 (0%) | 0 (0%) | |  | |  |  |  |  |  |  |  |  |
| **Empty stomach pain** |  |  |  | | 2.00 | | 2.00 | 0.37 | 0.09 |  |  |  |  |  |
| 1 | 10 (91%) | 10 (91%) | 10 (91%) | |  | |  |  |  |  |  |  |  |  |
| 2 | 0 (0%) | 1 (9.1%) | 0 (0%) | |  | |  |  |  |  |  |  |  |  |
| 3 | 1 (9.1%) | 0 (0%) | 1 (9.1%) | |  | |  |  |  |  |  |  |  |  |
| 4 | 0 (0%) | 0 (0%) | 0 (0%) | |  | |  |  |  |  |  |  |  |  |
| 5 | 0 (0%) | 0 (0%) | 0 (0%) | |  | |  |  |  |  |  |  |  |  |
| 6 | 0 (0%) | 0 (0%) | 0 (0%) | |  | |  |  |  |  |  |  |  |  |
| 7 | 0 (0%) | 0 (0%) | 0 (0%) | |  | |  |  |  |  |  |  |  |  |
| **Nausea** |  |  |  | | 2.00 | | 2.00 | 0.37 | 0.09 |  |  |  |  |  |
| 1 | 10 (91%) | 8 (73%) | 9 (82%) | |  | |  |  |  |  |  |  |  |  |
| 2 | 1 (9.1%) | 2 (18%) | 1 (9.1%) | |  | |  |  |  |  |  |  |  |  |
| 3 | 0 (0%) | 1 (9.1%) | 0 (0%) | |  | |  |  |  |  |  |  |  |  |
| 4 | 0 (0%) | 0 (0%) | 1 (9.1%) | |  | |  |  |  |  |  |  |  |  |
| 5 | 0 (0%) | 0 (0%) | 0 (0%) | |  | |  |  |  |  |  |  |  |  |
| 6 | 0 (0%) | 0 (0%) | 0 (0%) | |  | |  |  |  |  |  |  |  |  |
| 7 | 0 (0%) | 0 (0%) | 0 (0%) | |  | |  |  |  |  |  |  |  |  |
| **Stomach noise** |  |  |  | | 1.07 | | 2.00 | 0.59 | 0.05 |  |  |  |  |  |
| 1 | 2 (18%) | 5 (45%) | 5 (45%) | |  | |  |  |  |  |  |  |  |  |
| 2 | 6 (55%) | 3 (27%) | 4 (36%) | |  | |  |  |  |  |  |  |  |  |
| 3 | 3 (27%) | 1 (9.1%) | 2 (18%) | |  | |  |  |  |  |  |  |  |  |
| 4 | 0 (0%) | 2 (18%) | 0 (0%) | |  | |  |  |  |  |  |  |  |  |
| 5 | 0 (0%) | 0 (0%) | 0 (0%) | |  | |  |  |  |  |  |  |  |  |
| 6 | 0 (0%) | 0 (0%) | 0 (0%) | |  | |  |  |  |  |  |  |  |  |
| 7 | 0 (0%) | 0 (0%) | 0 (0%) | |  | |  |  |  |  |  |  |  |  |
| **Bloated stomach** |  |  |  | | 0.81 | | 2.00 | 0.67 | 0.04 |  |  |  |  |  |
| 1 | 5 (45%) | 5 (45%) | 5 (45%) | |  | |  |  |  |  |  |  |  |  |
| 2 | 2 (18%) | 2 (18%) | 4 (36%) | |  | |  |  |  |  |  |  |  |  |
| 3 | 1 (9.1%) | 1 (9.1%) | 1 (9.1%) | |  | |  |  |  |  |  |  |  |  |
| 4 | 2 (18%) | 1 (9.1%) | 1 (9.1%) | |  | |  |  |  |  |  |  |  |  |
| 5 | 1 (9.1%) | 1 (9.1%) | 0 (0%) | |  | |  |  |  |  |  |  |  |  |
| 6 | 0 (0%) | 1 (9.1%) | 0 (0%) | |  | |  |  |  |  |  |  |  |  |
| 7 | 0 (0%) | 0 (0%) | 0 (0%) | |  | |  |  |  |  |  |  |  |  |
| **Burping** |  |  |  | | 5.14 | | 2.00 | 0.08 | 0.23 |  |  |  |  |  |
| 1 | 6 (55%) | 8 (73%) | 10 (91%) | |  | |  |  | 0.16 | INU vs AX | 0.60 | 0.53 | 0.16 |  |
| 2 | 4 (36%) | 2 (18%) | 1 (9.1%) | |  | |  |  | 0.35 | PLA vs AX | 0.24 | 1.16 | 0.35 |  |
| 3 | 1 (9.1%) | 0 (0%) | 0 (0%) | |  | |  |  | 0.16 | PLA vs INU | 0.60 | 0.53 | 0.16 |  |
| 4 | 0 (0%) | 1 (9.1%) | 0 (0%) | |  | |  |  |  |  |  |  |  |  |
| 5 | 0 (0%) | 0 (0%) | 0 (0%) | |  | |  |  |  |  |  |  |  |  |
| 6 | 0 (0%) | 0 (0%) | 0 (0%) | |  | |  |  |  |  |  |  |  |  |
| 7 | 0 (0%) | 0 (0%) | 0 (0%) | |  | |  |  |  |  |  |  |  |  |
| **Flatuence** |  |  |  | | 5.84 | | 2.00 | 0.05 | 0.27 |  |  |  |  |  |
| 1 | 5 (45%) | 3 (27%) | 4 (36%) | |  | |  |  | 0.55 | INU vs AX | 0.07 | 1.82 | 0.55 |  |
| 2 | 2 (18%) | 1 (9.1%) | 4 (36%) | |  | |  |  | 0.00 | PLA vs AX | 1.00 | 0.00 | 0.00 |  |
| 3 | 2 (18%) | 0 (0%) | 1 (9.1%) | |  | |  |  | 0.55 | PLA vs INU | 0.07 | 1.82 | 0.55 |  |
| 4 | 1 (9.1%) | 2 (18%) | 0 (0%) | |  | |  |  |  |  |  |  |  |  |
| 5 | 1 (9.1%) | 0 (0%) | 2 (18%) | |  | |  |  |  |  |  |  |  |  |
| 6 | 0 (0%) | 4 (36%) | 0 (0%) | |  | |  |  |  |  |  |  |  |  |
| 7 | 0 (0%) | 1 (9.1%) | 0 (0%) | |  | |  |  |  |  |  |  |  |  |
| **Constipation** |  |  |  | | 1.41 | | 2.00 | 0.49 | 0.06 |  |  |  |  |  |
| 1 | 8 (73%) | 8 (73%) | 10 (91%) | |  | |  |  |  |  |  |  |  |  |
| 2 | 1 (9.1%) | 2 (18%) | 0 (0%) | |  | |  |  |  |  |  |  |  |  |
| 3 | 1 (9.1%) | 1 (9.1%) | 1 (9.1%) | |  | |  |  |  |  |  |  |  |  |
| 4 | 0 (0%) | 0 (0%) | 0 (0%) | |  | |  |  |  |  |  |  |  |  |
| 5 | 0 (0%) | 0 (0%) | 0 (0%) | |  | |  |  |  |  |  |  |  |  |
| 6 | 1 (9.1%) | 0 (0%) | 0 (0%) | |  | |  |  |  |  |  |  |  |  |
| 7 | 0 (0%) | 0 (0%) | 0 (0%) | |  | |  |  |  |  |  |  |  |  |
| **Diarrhea** |  |  |  | | 2.00 | | 2.00 | 0.37 | 0.09 |  |  |  |  |  |
| 1 | 10 (91%) | 9 (82%) | 10 (91%) | |  | |  |  |  |  |  |  |  |  |
| 2 | 1 (9.1%) | 1 (9.1%) | 1 (9.1%) | |  | |  |  |  |  |  |  |  |  |
| 3 | 0 (0%) | 0 (0%) | 0 (0%) | |  | |  |  |  |  |  |  |  |  |
| 4 | 0 (0%) | 0 (0%) | 0 (0%) | |  | |  |  |  |  |  |  |  |  |
| 5 | 0 (0%) | 1 (9.1%) | 0 (0%) | |  | |  |  |  |  |  |  |  |  |
| 6 | 0 (0%) | 0 (0%) | 0 (0%) | |  | |  |  |  |  |  |  |  |  |
| 7 | 0 (0%) | 0 (0%) | 0 (0%) | |  | |  |  |  |  |  |  |  |  |
| **Liquid stool** |  |  |  | |  | |  |  | 0.03 |  |  |  |  |  |
| 1 | 10 (91%) | 10 (91%) | 9 (82%) | |  | |  |  |  |  |  |  |  |  |
| 2 | 1 (9.1%) | 0 (0%) | 1 (9.1%) | |  | |  |  |  |  |  |  |  |  |
| 3 | 0 (0%) | 0 (0%) | 0 (0%) | |  | |  |  |  |  |  |  |  |  |
| 4 | 0 (0%) | 1 (9.1%) | 1 (9.1%) | |  | |  |  |  |  |  |  |  |  |
| 5 | 0 (0%) | 0 (0%) | 0 (0%) | |  | |  |  |  |  |  |  |  |  |
| 6 | 0 (0%) | 0 (0%) | 0 (0%) | |  | |  |  |  |  |  |  |  |  |
| 7 | 0 (0%) | 0 (0%) | 0 (0%) | |  | |  |  |  |  |  |  |  |  |
| **Hard stool** |  |  |  | | 0.11 | | 2.00 | 0.95 | 0.00 |  |  |  |  |  |
| 1 | 8 (73%) | 8 (73%) | 8 (73%) | |  | |  |  |  |  |  |  |  |  |
| 2 | 0 (0%) | 2 (18%) | 1 (9.1%) | |  | |  |  |  |  |  |  |  |  |
| 3 | 2 (18%) | 0 (0%) | 2 (18%) | |  | |  |  |  |  |  |  |  |  |
| 4 | 0 (0%) | 0 (0%) | 0 (0%) | |  | |  |  |  |  |  |  |  |  |
| 5 | 0 (0%) | 0 (0%) | 0 (0%) | |  | |  |  |  |  |  |  |  |  |
| 6 | 1 (9.1%) | 1 (9.1%) | 0 (0%) | |  | |  |  |  |  |  |  |  |  |
| 7 | 0 (0%) | 0 (0%) | 0 (0%) | |  | |  |  |  |  |  |  |  |  |
| **Toilet urge** |  |  |  | | 0.52 | | 2.00 | 0.77 | 0.02 |  |  |  |  |  |
| 1 | 5 (45%) | 7 (64%) | 6 (55%) | |  | |  |  |  |  |  |  |  |  |
| 2 | 4 (36%) | 2 (18%) | 4 (36%) | |  | |  |  |  |  |  |  |  |  |
| 3 | 1 (9.1%) | 1 (9.1%) | 1 (9.1%) | |  | |  |  |  |  |  |  |  |  |
| 4 | 1 (9.1%) | 1 (9.1%) | 0 (0%) | |  | |  |  |  |  |  |  |  |  |
| 5 | 0 (0%) | 0 (0%) | 0 (0%) | |  | |  |  |  |  |  |  |  |  |
| 6 | 0 (0%) | 0 (0%) | 0 (0%) | |  | |  |  |  |  |  |  |  |  |
| 7 | 0 (0%) | 0 (0%) | 0 (0%) | |  | |  |  |  |  |  |  |  |  |
| **Incomplete bowel emptying** | |  | |  | | 4.80 | 2.00 | 0.09 | 0.22 |  |  |  |  |  |
| 1 | 11 (100%) | 7 (64%) | 9 (82%) | |  | |  |  | 0.35 | INU vs AX | 0.24 | 1.16 | 0.35 |  |
| 2 | 0 (0%) | 3 (27%) | 2 (18%) | |  | |  |  | 0.16 | PLA vs AX | 0.60 | 0.53 | 0.16 |  |
| 3 | 0 (0%) | 0 (0%) | 0 (0%) | |  | |  |  | 0.16 | PLA vs INU | 0.60 | 0.53 | 0.16 |  |
| 4 | 0 (0%) | 0 (0%) | 0 (0%) | |  | |  |  |  |  |  |  |  |  |
| 5 | 0 (0%) | 1 (9.1%) | 0 (0%) | |  | |  |  |  |  |  |  |  |  |
| 6 | 0 (0%) | 0 (0%) | 0 (0%) | |  | |  |  |  |  |  |  |  |  |
| 7 | 0 (0%) | 0 (0%) | 0 (0%) | |  | |  |  |  |  |  |  |  |  |

| **P-types** | | | | | | | | | | | | | | | | | | | | |  |
| --- | --- | --- | --- | --- | --- | --- | --- | --- | --- | --- | --- | --- | --- | --- | --- | --- | --- | --- | --- | --- | --- |
|  |  |  |  | |  |  |  | | |  | | *Pairwise Eisinger post hoc test* | | | | | | | | | |
|  | ****AX**  N = 11** | ****INU**  N = 11** | ****PLA**  N = 11** | | **Statistic** | **df** | **p_value** | | | **EffectSize** | | **Comparison** | | | **Posthoc_pval** | | | **Z_value** | | **effsize_r** | |
| **Abdominal pain** | |  |  | | 1.39 | 2.00 | | 0.50 | 0.06 | |  | |  | | | |  | |  | |  |
| 1 | 7 (64%) | 5 (45%) | 5 (45%) | |  |  |  | | |  | |  | |  | | |  | |  | |  |
| 2 | 2 (18%) | 1 (9.1%) | 3 (27%) | |  |  |  | | |  | |  | |  | | |  | |  | |  |
| 3 | 2 (18%) | 2 (18%) | 2 (18%) | |  |  |  | | |  | |  | |  | | |  | |  | |  |
| 4 | 0 (0%) | 1 (9.1%) | 1 (9.1%) | |  |  |  | | |  | |  | |  | | |  | |  | |  |
| 5 | 0 (0%) | 0 (0%) | 0 (0%) | |  |  |  | | |  | |  | |  | | |  | |  | |  |
| 6 | 0 (0%) | 1 (9.1%) | 0 (0%) | |  |  |  | | |  | |  | |  | | |  | |  | |  |
| 7 | 0 (0%) | 1 (9.1%) | 0 (0%) | |  |  |  | | |  | |  | |  | | |  | |  | |  |
| **Chest pain** |  |  |  | | 2.00 | 2.00 | 0.37 | | | 0.09 | |  | |  | | |  | |  | |  |
| 1 | 11 (100%) | 9 (82%) | 10 (91%) | |  |  |  | | |  | |  | |  | | |  | |  | |  |
| 2 | 0 (0%) | 1 (9.1%) | 0 (0%) | |  |  |  | | |  | |  | |  | | |  | |  | |  |
| 3 | 0 (0%) | 1 (9.1%) | 1 (9.1%) | |  |  |  | | |  | |  | |  | | |  | |  | |  |
| 4 | 0 (0%) | 0 (0%) | 0 (0%) | |  |  |  | | |  | |  | |  | | |  | |  | |  |
| 5 | 0 (0%) | 0 (0%) | 0 (0%) | |  |  |  | | |  | |  | |  | | |  | |  | |  |
| 6 | 0 (0%) | 0 (0%) | 0 (0%) | |  |  |  | | |  | |  | |  | | |  | |  | |  |
| 7 | 0 (0%) | 0 (0%) | 0 (0%) | |  |  |  | | |  | |  | |  | | |  | |  | |  |
| **Heartburn** |  |  |  | | 2.60 | 2.00 | 0.27 | | | 0.12 | |  | |  | | |  | |  | |  |
| 1 | 9 (82%) | 11 (100%) | 9 (82%) | |  |  |  | | |  | |  | |  | | |  | |  | |  |
| 2 | 1 (9.1%) | 0 (0%) | 1 (9.1%) | |  |  |  | | |  | |  | |  | | |  | |  | |  |
| 3 | 0 (0%) | 0 (0%) | 1 (9.1%) | |  |  |  | | |  | |  | |  | | |  | |  | |  |
| 4 | 0 (0%) | 0 (0%) | 0 (0%) | |  |  |  | | |  | |  | |  | | |  | |  | |  |
| 5 | 0 (0%) | 0 (0%) | 0 (0%) | |  |  |  | | |  | |  | |  | | |  | |  | |  |
| 6 | 1 (9.1%) | 0 (0%) | 0 (0%) | |  |  |  | | |  | |  | |  | | |  | |  | |  |
| 7 | 0 (0%) | 0 (0%) | 0 (0%) | |  |  |  | | |  | |  | |  | | |  | |  | |  |
| **Empty stomach pain** | |  |  | | 2.00 | 2.00 | | 0.37 | 0.09 | |  | | | | |  |  | |  | |  |
| 1 | 10 (91%) | 11 (100%) | 11 (100%) | |  |  |  | | |  | |  | |  | | |  | |  | |  |
| 2 | 1 (9.1%) | 0 (0%) | 0 (0%) | |  |  |  | | |  | |  | |  | | |  | |  | |  |
| 3 | 0 (0%) | 0 (0%) | 0 (0%) | |  |  |  | | |  | |  | |  | | |  | |  | |  |
| 4 | 0 (0%) | 0 (0%) | 0 (0%) | |  |  |  | | |  | |  | |  | | |  | |  | |  |
| 5 | 0 (0%) | 0 (0%) | 0 (0%) | |  |  |  | | |  | |  | |  | | |  | |  | |  |
| 6 | 0 (0%) | 0 (0%) | 0 (0%) | |  |  |  | | |  | |  | |  | | |  | |  | |  |
| 7 | 0 (0%) | 0 (0%) | 0 (0%) | |  |  |  | | |  | |  | |  | | |  | |  | |  |
| **Nausea** |  |  |  | | 0.00 | 2.00 | 1.00 | | | 0.00 | |  | |  | | |  | |  | |  |
| 1 | 10 (91%) | 9 (82%) | 10 (91%) | |  |  |  | | |  | |  | |  | | |  | |  | |  |
| 2 | 0 (0%) | 2 (18%) | 0 (0%) | |  |  |  | | |  | |  | |  | | |  | |  | |  |
| 3 | 1 (9.1%) | 0 (0%) | 1 (9.1%) | |  |  |  | | |  | |  | |  | | |  | |  | |  |
| 4 | 0 (0%) | 0 (0%) | 0 (0%) | |  |  |  | | |  | |  | |  | | |  | |  | |  |
| 5 | 0 (0%) | 0 (0%) | 0 (0%) | |  |  |  | | |  | |  | |  | | |  | |  | |  |
| 6 | 0 (0%) | 0 (0%) | 0 (0%) | |  |  |  | | |  | |  | |  | | |  | |  | |  |
| 7 | 0 (0%) | 0 (0%) | 0 (0%) | |  |  |  | | |  | |  | |  | | |  | |  | |  |
| **Stomach noise** | |  |  | | 2.77 | 2.00 | | 0.25 | 0.13 | |  | | | | |  |  | |  | |  |
| 1 | 5 (45%) | 3 (27%) | 5 (45%) | |  |  |  | | |  | |  | |  | | |  | |  | |  |
| 2 | 5 (45%) | 4 (36%) | 2 (18%) | |  |  |  | | |  | |  | |  | | |  | |  | |  |
| 3 | 1 (9.1%) | 0 (0%) | 2 (18%) | |  |  |  | | |  | |  | |  | | |  | |  | |  |
| 4 | 0 (0%) | 2 (18%) | 1 (9.1%) | |  |  |  | | |  | |  | |  | | |  | |  | |  |
| 5 | 0 (0%) | 1 (9.1%) | 1 (9.1%) | |  |  |  | | |  | |  | |  | | |  | |  | |  |
| 6 | 0 (0%) | 0 (0%) | 0 (0%) | |  |  |  | | |  | |  | |  | | |  | |  | |  |
| 7 | 0 (0%) | 1 (9.1%) | 0 (0%) | |  |  |  | | |  | |  | |  | | |  | |  | |  |
| **Bloated stomach** | |  |  | | 0.96 | 2.00 | | 0.62 | 0.04 | |  | | | | |  |  | |  | |  |
| 1 | 3 (30%) | 5 (45%) | 6 (55%) | |  |  |  | | |  | |  | |  | | |  | |  | |  |
| 2 | 4 (40%) | 1 (9.1%) | 1 (9.1%) | |  |  |  | | |  | |  | |  | | |  | |  | |  |
| 3 | 2 (20%) | 3 (27%) | 3 (27%) | |  |  |  | | |  | |  | |  | | |  | |  | |  |
| 4 | 0 (0%) | 0 (0%) | 0 (0%) | |  |  |  | | |  | |  | |  | | |  | |  | |  |
| 5 | 0 (0%) | 1 (9.1%) | 1 (9.1%) | |  |  |  | | |  | |  | |  | | |  | |  | |  |
| 6 | 1 (10%) | 0 (0%) | 0 (0%) | |  |  |  | | |  | |  | |  | | |  | |  | |  |
| 7 | 0 (0%) | 1 (9.1%) | 0 (0%) | |  |  |  | | |  | |  | |  | | |  | |  | |  |
| **Burping** |  |  |  | | 1.40 | 2.00 | 0.50 | | | 0.06 | |  | |  | | |  | |  | |  |
| 1 | 9 (82%) | 9 (82%) | 8 (73%) | |  |  |  | | |  | |  | |  | | |  | |  | |  |
| 2 | 2 (18%) | 1 (9.1%) | 1 (9.1%) | |  |  |  | | |  | |  | |  | | |  | |  | |  |
| 3 | 0 (0%) | 0 (0%) | 2 (18%) | |  |  |  | | |  | |  | |  | | |  | |  | |  |
| 4 | 0 (0%) | 0 (0%) | 0 (0%) | |  |  |  | | |  | |  | |  | | |  | |  | |  |
| 5 | 0 (0%) | 0 (0%) | 0 (0%) | |  |  |  | | |  | |  | |  | | |  | |  | |  |
| 6 | 0 (0%) | 0 (0%) | 0 (0%) | |  |  |  | | |  | |  | |  | | |  | |  | |  |
| 7 | 0 (0%) | 1 (9.1%) | 0 (0%) | |  |  |  | | |  | |  | |  | | |  | |  | |  |
| **Flatuence** |  |  |  | | 7.80 | 2.00 | 0.02 | | | 0.35 | |  | |  | | |  | |  | |  |
| 1 | 3 (27%) | 2 (18%) | 3 (27%) | |  |  |  | | | 0.69 | | INU vs AX | | 0.02 | | | 2.27 | | 0.69 | |  |
| 2 | 5 (45%) | 1 (9.1%) | 2 (18%) | |  |  |  | | | 0.16 | | PLA vs AX | | 0.60 | | | 0.53 | | 0.16 | |  |
| 3 | 2 (18%) | 3 (27%) | 2 (18%) | |  |  |  | | | 0.48 | | PLA vs INU | | 0.11 | | | 1.60 | | 0.48 | |  |
| 4 | 1 (9.1%) | 1 (9.1%) | 2 (18%) | |  |  |  | | |  | |  | |  | | |  | |  | |  |
| 5 | 0 (0%) | 0 (0%) | 2 (18%) | |  |  |  | | |  | |  | |  | | |  | |  | |  |
| 6 | 0 (0%) | 3 (27%) | 0 (0%) | |  |  |  | | |  | |  | |  | | |  | |  | |  |
| 7 | 0 (0%) | 1 (9.1%) | 0 (0%) | |  |  |  | | |  | |  | |  | | |  | |  | |  |
| **Constipation** | |  |  | | 9.00 | 2.00 | | 0.01 | 0.41 | |  | | | | |  |  | |  | |  |
| 1 | 8 (73%) | 10 (91%) | 4 (36%) | |  |  |  | | | 0.00 | | INU vs AX | | 1.00 | | | 0.00 | | 0.00 | |  |
| 2 | 2 (18%) | 0 (0%) | 2 (18%) | |  |  |  | | | 0.55 | | PLA vs AX | | 0.07 | | | 1.82 | | 0.55 | |  |
| 3 | 0 (0%) | 0 (0%) | 2 (18%) | |  |  |  | | | 0.55 | | PLA vs INU | | 0.07 | | | 1.82 | | 0.55 | |  |
| 4 | 1 (9.1%) | 0 (0%) | 1 (9.1%) | |  |  |  | | |  | |  | |  | | |  | |  | |  |
| 5 | 0 (0%) | 1 (9.1%) | 0 (0%) | |  |  |  | | |  | |  | |  | | |  | |  | |  |
| 6 | 0 (0%) | 0 (0%) | 1 (9.1%) | |  |  |  | | |  | |  | |  | | |  | |  | |  |
| 7 | 0 (0%) | 0 (0%) | 1 (9.1%) | |  |  |  | | |  | |  | |  | | |  | |  | |  |
| **Diarrhea** |  |  |  | | 6.00 | 2.00 | 0.05 | | | 0.27 | |  | |  | | |  | |  | |  |
| 1 | 9 (82%) | 7 (64%) | 9 (82%) | |  |  |  | | | 0.29 | | INU vs AX | | 0.34 | | | 0.95 | | 0.29 | |  |
| 2 | 2 (18%) | 2 (18%) | 2 (18%) | |  |  |  | | | 0.00 | | PLA vs AX | | 1.00 | | | 0.00 | | 0.00 | |  |
| 3 | 0 (0%) | 0 (0%) | 0 (0%) | |  |  |  | | | 0.29 | | PLA vs INU | | 0.34 | | | 0.95 | | 0.29 | |  |
| 4 | 0 (0%) | 0 (0%) | 0 (0%) | |  |  |  | | |  | |  | |  | | |  | |  | |  |
| 5 | 0 (0%) | 1 (9.1%) | 0 (0%) | |  |  |  | | |  | |  | |  | | |  | |  | |  |
| 6 | 0 (0%) | 1 (9.1%) | 0 (0%) | |  |  |  | | |  | |  | |  | | |  | |  | |  |
| 7 | 0 (0%) | 0 (0%) | 0 (0%) | |  |  |  | | |  | |  | |  | | |  | |  | |  |
| **Liquid stool** | |  |  | | 0.50 | 2.00 | | 0.78 | 0.02 | |  | | | | |  |  | |  | |  |
| 1 | 7 (64%) | 7 (64%) | 7 (64%) | |  |  |  | | |  | |  | |  | | |  | |  | |  |
| 2 | 2 (18%) | 2 (18%) | 3 (27%) | |  |  |  | | |  | |  | |  | | |  | |  | |  |
| 3 | 1 (9.1%) | 0 (0%) | 1 (9.1%) | |  |  |  | | |  | |  | |  | | |  | |  | |  |
| 4 | 0 (0%) | 2 (18%) | 0 (0%) | |  |  |  | | |  | |  | |  | | |  | |  | |  |
| 5 | 1 (9.1%) | 0 (0%) | 0 (0%) | |  |  |  | | |  | |  | |  | | |  | |  | |  |
| 6 | 0 (0%) | 0 (0%) | 0 (0%) | |  |  |  | | |  | |  | |  | | |  | |  | |  |
| 7 | 0 (0%) | 0 (0%) | 0 (0%) | |  |  |  | | |  | |  | |  | | |  | |  | |  |
| **Hard stool** |  |  |  | | 9.77 | 2.00 | 0.01 | | | 0.44 | |  | |  | | |  | |  | |  |
| 1 | 8 (73%) | 10 (91%) | 3 (27%) | |  |  |  | | | 0.03 | | INU vs AX | | 0.92 | | | 0.10 | | 0.03 | |  |
| 2 | 2 (18%) | 0 (0%) | 3 (27%) | |  |  |  | | | 0.62 | | PLA vs AX | | 0.04 | | | 2.04 | | 0.62 | |  |
| 3 | 0 (0%) | 0 (0%) | 3 (27%) | |  |  |  | | | 0.62 | | PLA vs INU | | 0.04 | | | 2.04 | | 0.62 | |  |
| 4 | 1 (9.1%) | 0 (0%) | 0 (0%) | |  |  |  | | |  | |  | |  | | |  | |  | |  |
| 5 | 0 (0%) | 0 (0%) | 0 (0%) | |  |  |  | | |  | |  | |  | | |  | |  | |  |
| 6 | 0 (0%) | 1 (9.1%) | 2 (18%) | |  |  |  | | |  | |  | |  | | |  | |  | |  |
| 7 | 0 (0%) | 0 (0%) | 0 (0%) | |  |  |  | | |  | |  | |  | | |  | |  | |  |
| **Toilet urge** |  |  |  | | 0.29 | 2.00 | 0.87 | | | 0.01 | |  | |  | | |  | |  | |  |
| 1 | 6 (55%) | 4 (36%) | 7 (64%) | |  |  |  | | |  | |  | |  | | |  | |  | |  |
| 2 | 1 (9.1%) | 4 (36%) | 1 (9.1%) | |  |  |  | | |  | |  | |  | | |  | |  | |  |
| 3 | 2 (18%) | 0 (0%) | 1 (9.1%) | |  |  |  | | |  | |  | |  | | |  | |  | |  |
| 4 | 1 (9.1%) | 1 (9.1%) | 1 (9.1%) | |  |  |  | | |  | |  | |  | | |  | |  | |  |
| 5 | 1 (9.1%) | 1 (9.1%) | 0 (0%) | |  |  |  | | |  | |  | |  | | |  | |  | |  |
| 6 | 0 (0%) | 1 (9.1%) | 1 (9.1%) | |  |  |  | | |  | |  | |  | | |  | |  | |  |
| 7 | 0 (0%) | 0 (0%) | 0 (0%) | |  |  |  | | |  | |  | |  | | |  | |  | |  |
| **Incomplete bowel emptying** | | | |  | 2.70 | 2.00 | | 0.26 | 0.12 | |  | | | | |  |  | |  | |  |
| 1 | 7 (64%) | 8 (73%) | 6 (55%) | |  |  |  | | |  | |  | |  | | |  | |  | |  |
| 2 | 2 (18%) | 2 (18%) | 1 (9.1%) | |  |  |  | | |  | |  | |  | | |  | |  | |  |
| 3 | 1 (9.1%) | 0 (0%) | 1 (9.1%) | |  |  |  | | |  | |  | |  | | |  | |  | |  |
| 4 | 1 (9.1%) | 0 (0%) | 1 (9.1%) | |  |  |  | | |  | |  | |  | | |  | |  | |  |
| 5 | 0 (0%) | 0 (0%) | 2 (18%) | |  |  |  | | |  | |  | |  | | |  | |  | |  |
| 6 | 0 (0%) | 1 (9.1%) | 0 (0%) | |  |  |  | | |  | |  | |  | | |  | |  | |  |
| 7 | 0 (0%) | 0 (0%) | 0 (0%) | |  |  |  | | |  | |  | |  | | |  | |  | |  |

Data are presented as n (%). Differences between treatments were analyzed using the Friedman test, with Kendall’s W reported as the effect size. For p < 0.1, post hoc pairwise comparisons were performed using the Eisinger method with least significant difference (LSD) adjustment. A p < 0.05 was considered statistically significant. Effect sizes for post hoc tests are reported as r. The GSRS is a 7-point scale ranging from 1 (no discomfort at all) to 7 (very severe discomfort). Abbreviations: Abbreviations: AX, arabinoxylan; B-type, *Bacteroides*-type; df, degrees of freedom; EffectSize, effect size; effsize_r, effect size r; INU, inulin; N, number of participants; PLA, placebo; Posthoc_pval, adjusted post hoc p-value; P-type, *Prevotella*-type; Z_value, standardized test statistic.

**Supplementary Table 4: Energy intake and dietary composition 24 hours prior to clinical investigation day (CID).**

| **B-types** | | | | | | | | | | | |
| --- | --- | --- | --- | --- | --- | --- | --- | --- | --- | --- | --- |
|  |  |  |  |  |  |  |  |  | *Pairwise post hoc test* | | |
|  | ****AX**  N = 11** | ****INU**  N = 11** | ****PLA**  N = 11** | Statistic | df1 | df2 | p_value | EffectSize_ANOVA | Comparison | Posthoc_pval | Cohens_d |
| **Energy (kcal)** | 1860 (550) | 2094 (795) | 2384 (792) | 1.79 | 2 | 20 | 0.19 | 0.09 |  |  |  |
| **Protein (g) / 1000 kcal** | 35 (9) | 36 (10) | 35 (9) | 0.04 | 2 | 20 | 0.96 | 0.00 |  |  |  |
|  |  |  |  |  |  |  |  |  |  |  |  |
|  |  |  |  |  |  |  |  |  |  |  |  |
|  |  |  |  |  |  |  |  |  |  |  |  |
| **Total fat (g) / 1000 kcal** | 45 (9) | 48 (9) | 45 (7) | 0.59 | 2 | 20 | 0.56 | 0.04 |  |  |  |
| **Carbohydrates (g) / 1000 kcal** | 106 (16) | 98 (16) | 103 (15) | 0.74 | 2 | 20 | 0.49 | 0.05 |  |  |  |
| **Fiber (g) / 1000 kcal** | 13.7 (4.6) | 12.4 (5.4) | 13.2 (4.4) | 0.37 | 2 | 20 | 0.69 | 0.01 |  |  |  |
|  |  |  |  |  |  |  |  |  | AX vs INU | 0.54 | 0.19 |
|  |  |  |  |  |  |  |  |  | AX vs PLA | 0.06 | 0.64 |
|  |  |  |  |  |  |  |  |  | INU vs PLA | 0.09 | 0.57 |
| **P-types** | | | | | | | | | | | |
|  |  |  |  |  |  |  |  |  |  |  |  |
|  | ****AX**  N = 11** | ****INU**  N = 11** | ****PLA**  N = 11** | Statistic | df1 | df2 | p_value | EffectSize_ANOVA | Comparison | Posthoc_pval | Cohens_d |
| **Energy (kcal)** | 1979 (468) | 1706 (585) | 1916 (558) | 1.21 | 2 | 20 | 0.32 | 0.05 |  |  |  |
| **Protein (g) / 1000 kcal** | 41 (7) | 35 (12) | 45 (14) | 2.71 | 2 | 20 | 0.09 | 0.12 |  |  |  |
|  |  |  |  |  |  |  |  |  | AX vs INU | 0.16 | 0.46 |
|  |  |  |  |  |  |  |  |  | AX vs PLA | 0.22 | 0.40 |
|  |  |  |  |  |  |  |  |  | INU vs PLA | 0.10 | 0.54 |
| **Total fat (g) / 1000 kcal** | 46 (9) | 42 (7) | 41 (12) | 1.34 | 2 | 20 | 0.28 | 0.05 |  |  |  |
| **Carbohydrates (g) / 1000 kcal** | 101 (24) | 108 (21) | 102 (25) | 0.59 | 2 | 20 | 0.56 | 0.02 |  |  |  |
| **Fiber (g) / 1000 kcal** | 14.4 (5.0) | 14.6 (6.5) | 15.9 (6.1) | 0.45 | 2 | 20 | 0.65 | 0.01 |  |  |  |

Data are presented as mean (SD). Differences between treatments were assessed using repeated-measures ANOVA. If p < 0.1, post hoc pairwise comparisons were conducted using paired t-tests with least significant difference (LSD) adjustment. Effect sizes for post hoc comparisons are reported as Cohen’s d. A p-value < 0.05 was considered statistically significant. Abbreviations: ANOVA, analysis of variance; AX, arabinoxylan; B-type, *Bacteroides*-type; CID, clinical investigation day; df1/df2, numerator and denominator degrees of freedom; INU, inulin; N, number of participants; PLA, placebo; Posthoc_pval, adjusted post hoc p-value; P-type, *Prevotella*-type.

**Supplementary Table 5. Intervention effects assessed using repeated-measures ANOVA.**

| **B-types** | | | | | | | | | | | | |
| --- | --- | --- | --- | --- | --- | --- | --- | --- | --- | --- | --- | --- |
|  |  |  |  |  |  |  |  |  | *Pairwise post hoc test* | | |  |
|  | ****AX**  N = 11** | ****INU**  N = 11** | ****PLA**  N = 11** | **Statistic** | **df1** | **df** | **p_value** | **EffectSize_ANOVA** | **Comparison** | **Posthoc_pval** | **Cohens_d** |  |
|  |  |  |  |  |  |  |  |  |  |  |  |  |
| **SCFAs and H2** |  |  |  |  |  |  |  |  |  |  |  |  |
|  |  |  |  |  |  |  |  |  |  |  |  |  |
| **Acetate** |  |  |  |  |  |  |  |  |  |  |  |  |
| *Fasting* | 35 (13, 51) | 16 (7, 32) | 20 (13, 26) | 2.86 | 2 | 20 | 0.08 | 0.10 |  |  |  |  |
|  |  |  |  |  |  |  |  |  | **AX vs INU** | **0.01** | **0.96** |  |
|  |  |  |  |  |  |  |  |  | AX vs PLA | 0.10 | 0.54 |  |
|  |  |  |  |  |  |  |  |  | INU vs PLA | 0.85 | 0.06 |  |
|  |  |  |  |  |  |  |  |  |  |  |  |  |
| *AUC 0-180* | 2,104 (691, 2,997) | 974 (340, 1,952) | 947 (713, 1,248) | 4.82 | 2 | 20 | 0.02 | 0.12 |  |  |  |  |
|  |  |  |  |  |  |  |  |  | **AX vs INU** | **0.02** | **0.82** |  |
|  |  |  |  |  |  |  |  |  | **AX vs PLA** | **0.05** | **0.68** |  |
|  |  |  |  |  |  |  |  |  | INU vs PLA | 0.64 | 0.14 |  |
|  |  |  |  |  |  |  |  |  |  |  |  |  |
| *AUC 0-60* | 2,070 (515, 2,347) | 586 (340, 1,307) | 681 (548, 1,020) | 5.13 | 2 | 20 | 0.02 | 0.14 |  |  |  |  |
|  |  |  |  |  |  |  |  |  | **AX vs INU** | **0.02** | **0.81** |  |
|  |  |  |  |  |  |  |  |  | **AX vs PLA** | **0.04** | **0.69** |  |
|  |  |  |  |  |  |  |  |  | INU vs PLA | 0.77 | 0.09 |  |
|  |  |  |  |  |  |  |  |  |  |  |  |  |
| *AUC 60-180* | 462 (55, 1,692) | 521 (10, 845) | 233 (217, 751) | 3.38 | 2 | 20 | 0.05 | 0.09 |  |  |  |  |
|  |  |  |  |  |  |  |  |  | AX vs INU | 0.07 | 0.60 |  |
|  |  |  |  |  |  |  |  |  | AX vs PLA | 0.07 | 0.61 |  |
|  |  |  |  |  |  |  |  |  | INU vs PLA | 0.77 | 0.09 |  |
|  |  |  |  |  |  |  |  |  |  |  |  |  |
|  |  |  |  |  |  |  |  |  |  |  |  |  |
| **Propionate** |  |  |  |  |  |  |  |  |  |  |  |  |
| *Fasting^1^* | 1.43 (1.25, 4.07) | 1.00 (0.70, 1.82) | 1.57 (1.21, 1.82) | 5.08 | 2 | 20 | 0.02 | 0.15 |  |  |  |  |
|  |  |  |  |  |  |  |  |  | **AX vs INU** | **0.02** | **0.85** |  |
|  |  |  |  |  |  |  |  |  | AX vs PLA | 0.11 | 0.53 |  |
|  |  |  |  |  |  |  |  |  | INU vs PLA | 0.12 | 0.51 |  |
|  |  |  |  |  |  |  |  |  |  |  |  |  |
| *AUC 0-180* | 284 (193, 389) | 224 (150, 302) | 238 (184, 289) | 4.65 | 2 | 20 | 0.02 | 0.08 |  |  |  |  |
|  |  |  |  |  |  |  |  |  | **AX vs INU** | **0.02** | **0.84** |  |
|  |  |  |  |  |  |  |  |  | AX vs PLA | 0.07 | 0.62 |  |
|  |  |  |  |  |  |  |  |  | INU vs PLA | 0.38 | 0.28 |  |
|  |  |  |  |  |  |  |  |  |  |  |  |  |
| *AUC 0-60* | 190 (140, 266) | 148 (100, 220) | 161 (125, 181) | 5.34 | 2 | 20 | 0.01 | 0.10 |  |  |  |  |
|  |  |  |  |  |  |  |  |  | **AX vs INU** | **0.02** | **0.85** |  |
|  |  |  |  |  |  |  |  |  | AX vs PLA | 0.05 | 0.66 |  |
|  |  |  |  |  |  |  |  |  | INU vs PLA | 0.31 | 0.32 |  |
|  |  |  |  |  |  |  |  |  |  |  |  |  |
| *AUC 60-180* | 195 (123, 252) | 155 (100, 188) | 155 (106, 199) | 1.93 | 2 | 20 | 0.17 | 0.04 |  |  |  |  |
|  |  |  |  |  |  |  |  |  |  |  |  |  |
| **Butyrate** |  |  |  |  |  |  |  |  |  |  |  |  |
| *Fasting* | 0.44 (0.25, 0.62) | 0.41 (0.23, 0.73) | 0.40 (0.22, 0.70) | 1.76 | 2 | 20 | 0.20 | 0.04 |  |  |  |  |
| *AUC 0-180^1^* | 69 (57, 113) | 70 (34, 162) | 62 (37, 126) | 1.06 | 2 | 20 | 0.37 | 0.01 |  |  |  |  |
| *AUC 0-60^1^* | 54 (41, 86) | 54 (24, 127) | 50 (29, 93) | 0.91 | 2 | 20 | 0.42 | 0.01 |  |  |  |  |
| *AUC 60-180^1^* | 38 (33, 63) | 38 (19, 54) | 30 (17, 76) | 1.03 | 2 | 20 | 0.38 | 0.01 |  |  |  |  |
|  |  |  |  |  |  |  |  |  |  |  |  |  |
| **Isobutyrate** |  |  |  |  |  |  |  |  |  |  |  |  |
| *Fasting* | 1.29 (1.11, 1.81) | 1.07 (0.44, 1.53) | 1.43 (1.15, 1.72) | 7.76 | 2 | 20 | <0.01 | 0.14 |  |  |  |  |
|  |  |  |  |  |  |  |  |  | **AX vs INU** | **<0.01** | **1.37** |  |
|  |  |  |  |  |  |  |  |  | AX vs PLA | 0.44 | 0.24 |  |
|  |  |  |  |  |  |  |  |  | **INU vs PLA** | **0.04** | **0.73** |  |
|  |  |  |  |  |  |  |  |  |  |  |  |  |
| *AUC 0-180* | 193 (168, 208) | 166 (144, 211) | 189 (178, 203) | 1.06 | 2 | 20 | 0.36 | 0.04 |  |  |  |  |
| *AUC 0-60* | 129 (111, 151) | 116 (99, 143) | 123 (119, 144) | 1.18 | 2 | 20 | 0.33 | 0.06 |  |  |  |  |
| *AUC 60-180* | 106 (100, 134) | 109 (94, 135) | 114 (106, 136) | 0.15 | 2 | 20 | 0.86 | 0.01 |  |  |  |  |
|  |  |  |  |  |  |  |  |  |  |  |  |  |
| **2-Methyl-butyrate** |  |  |  |  |  |  |  |  |  |  |  |  |
| *Fasting^1^* | 2.81 (1.84, 3.12) | 1.80 (1.04, 2.77) | 2.85 (2.25, 3.28) | 6.44 | 2 | 20 | 0.01 | 0.14 |  |  |  |  |
|  |  |  |  |  |  |  |  |  | **AX vs INU** | **<0.01** | **1.21** |  |
|  |  |  |  |  |  |  |  |  | AX vs PLA | 0.94 | 0.03 |  |
|  |  |  |  |  |  |  |  |  | **INU vs PLA** | **0.04** | **0.72** |  |
|  |  |  |  |  |  |  |  |  |  |  |  |  |
| *AUC 0-180* | 449 (318, 467) | 426 (244, 465) | 393 (334, 501) | 0.33 | 2 | 20 | 0.72 | 0.01 |  |  |  |  |
| *AUC 0-60* | 294 (269, 323) | 289 (164, 336) | 267 (224, 351) | 0.34 | 2 | 20 | 0.71 | 0.01 |  |  |  |  |
| *AUC 60-180* | 265 (193, 297) | 277 (164, 299) | 269 (208, 297) | 0 | 2 | 20 | 1.00 | 0.00 |  |  |  |  |
|  |  |  |  |  |  |  |  |  |  |  |  |  |
| **Isovalerate** |  |  |  |  |  |  |  |  |  |  |  |  |
| *Fasting^1^* | 1.26 (0.94, 1.77) | 1.06 (0.48, 1.77) | 1.74 (1.14, 2.35) | 5.19 | 2 | 20 | 0.02 | 0.11 |  |  |  |  |
|  |  |  |  |  |  |  |  |  | **AX vs INU** | **0.01** | **0.92** |  |
|  |  |  |  |  |  |  |  |  | AX vs PLA | 0.34 | 0.30 |  |
|  |  |  |  |  |  |  |  |  | **INU vs PLA** | **0.03** | **0.76** |  |
|  |  |  |  |  |  |  |  |  |  |  |  |  |
| *AUC 0-180^1^* | 256 (144, 301) | 206 (158, 321) | 224 (184, 339) | 1.23 | 2 | 20 | 0.31 | 0.03 |  |  |  |  |
| *AUC 0-60^1^* | 169 (111, 206) | 143 (100, 226) | 165 (124, 216) | 1.27 | 2 | 20 | 0.30 | 0.03 |  |  |  |  |
| *AUC 60-180^1^* | 163 (84, 188) | 135 (104, 195) | 151 (117, 201) | 1.25 | 2 | 20 | 0.31 | 0.03 |  |  |  |  |
|  |  |  |  |  |  |  |  |  |  |  |  |  |
| **Valerate** |  |  |  |  |  |  |  |  |  |  |  |  |
| *Fasting^1^* | 0.16 (0.10, 0.21) | 0.08 (0.04, 0.20) | 0.13 (0.10, 0.26) | 1.84 | 2 | 20 | 0.19 | 0.05 |  |  |  |  |
| *AUC 0-180* | 24 (9, 36) | 23 (9, 35) | 21 (13, 41) | 0.05 | 2 | 20 | 0.95 | 0.00 |  |  |  |  |
| *AUC 0-60* | 14 (5, 25) | 15 (6, 23) | 14 (9, 27) | 0.43 | 2 | 20 | 0.66 | 0.00 |  |  |  |  |
| *AUC 60-180* | 16 (6, 23) | 15 (7, 24) | 14 (8, 26) | 0.24 | 2 | 20 | 0.79 | 0.00 |  |  |  |  |
|  |  |  |  |  |  |  |  |  |  |  |  |  |
| **Caproic acid** |  |  |  |  |  |  |  |  |  |  |  |  |
| *Fasting^1^* | 0.63 (0.42, 1.64) | 0.75 (0.43, 1.14) | 0.74 (0.46, 1.30) | 1.28 | 2 | 20 | 0.30 | 0.02 |  |  |  |  |
| *AUC 0-180^1^* | 127 (100, 192) | 133 (85, 223) | 138 (99, 166) | 0.66 | 2 | 20 | 0.53 | 0.01 |  |  |  |  |
| *AUC 0-60^1^* | 91 (63, 125) | 95 (56, 122) | 94 (56, 120) | 0.87 | 2 | 20 | 0.43 | 0.01 |  |  |  |  |
| *AUC 60-180^1^* | 81 (65, 121) | 81 (65, 165) | 89 (71, 121) | 1.03 | 2 | 20 | 0.37 | 0.01 |  |  |  |  |
|  |  |  |  |  |  |  |  |  |  |  |  |  |
| **H2** |  |  |  |  |  |  |  |  |  |  |  |  |
| *Fasting^1^* | 9 (3, 29) | 13 (9, 51) | 13 (6, 37) | 1.49 | 2 | 20 | 0.25 | 0.06 |  |  |  |  |
| *AUC 0-180^1^* | 623 (540, 2,040) | 1,793 (795, 8,528) | 2,340 (623, 4,380) | 2.02 | 2 | 20 | 0.16 | 0.08 |  |  |  |  |
| *AUC 0-60^1^* | 435 (345, 1,335) | 1,185 (570, 4,133) | 1,005 (390, 2,355) | 1.77 | 2 | 20 | 0.20 | 0.07 |  |  |  |  |
| *AUC 60-180^1^* | 330 (203, 878) | 765 (330, 5,235) | 1,140 (308, 2,265) | 1.67 | 2 | 20 | 0.21 | 0.07 |  |  |  |  |
|  |  |  |  |  |  |  |  |  |  |  |  |  |
| **Metabolites** |  |  |  |  |  |  |  |  |  |  |  |  |
| **Glucose** |  |  |  |  |  |  |  |  |  |  |  |  |
| *Fasting* | 4.16 (3.89, 4.55) | 4.33 (4.05, 4.55) | 4.22 (3.83, 4.38) | 2.73 | 2 | 20 | 0.09 | 0.07 |  |  |  |  |
|  |  |  |  |  |  |  |  |  | AX vs INU | 0.12 | 0.52 |  |
|  |  |  |  |  |  |  |  |  | AX vs PLA | 0.83 | 0.07 |  |
|  |  |  |  |  |  |  |  |  | **INU vs PLA** | **0.04** | **0.70** |  |
| *AUC 0-180^1^* | 634 (583, 720) | 617 (589, 753) | 653 (552, 738) | 0.19 | 2 | 20 | 0.83 | 0.00 |  |  |  |  |
| *AUC 0-60^1^* | 433 (373, 492) | 413 (384, 512) | 428 (364, 524) | 0.16 | 2 | 20 | 0.85 | 0.00 |  |  |  |  |
| *AUC 60-180^1^* | 411 (331, 441) | 396 (370, 446) | 411 (350, 436) | 0.05 | 2 | 20 | 0.95 | 0.00 |  |  |  |  |
|  |  |  |  |  |  |  |  |  |  |  |  |  |
| **Insulin** |  |  |  |  |  |  |  |  |  |  |  |  |
| *Fasting^1^* | 5.6 (4.3, 12.2) | 6.1 (4.4, 13.0) | 5.5 (3.7, 10.0) | 0.28 | 2 | 20 | 0.76 | 0.01 |  |  |  |  |
| *AUC 0-180^1^* | 4,496 (3,372, 10,559) | 3,747 (3,243, 6,359) | 5,400 (3,311, 9,698) | 2.4 | 2 | 20 | 0.12 | 0.02 |  |  |  |  |
| *AUC 0-60^1^* | 3,854 (2,858, 8,891) | 3,069 (2,709, 4,922) | 3,858 (2,816, 8,084) | 2.21 | 2 | 20 | 0.14 | 0.01 |  |  |  |  |
| *AUC 60-180^1^* | 2,181 (1,596, 5,280) | 2,082 (1,437, 3,612) | 2,718 (1,710, 5,592) | 2.13 | 2 | 20 | 0.15 | 0.02 |  |  |  |  |
|  |  |  |  |  |  |  |  |  |  |  |  |  |
| **Cholesterol** |  |  |  |  |  |  |  |  |  |  |  |  |
| *Fasting^1^* | 4.19 (3.61, 4.81) | 4.45 (3.77, 5.56) | 4.24 (3.82, 4.89) | 3.74 | 2 | 20 | 0.04 | 0.07 |  |  |  |  |
|  |  |  |  |  |  |  |  |  | **AX vs INU** | **0.04** | **0.71** |  |
|  |  |  |  |  |  |  |  |  | AX vs PLA | 0.74 | 0.10 |  |
|  |  |  |  |  |  |  |  |  | INU vs PLA | 0.06 | 0.64 |  |
| *AUC 0-180^1^* | 782 (712, 849) | 814 (695, 938) | 781 (661, 828) | 3.01 | 2 | 20 | 0.07 | 0.06 |  |  |  |  |
|  |  |  |  |  |  |  |  |  | AX vs INU | 0.32 | 0.32 |  |
|  |  |  |  |  |  |  |  |  | AX vs PLA | 0.19 | 0.42 |  |
|  |  |  |  |  |  |  |  |  | **INU vs PLA** | **0.04** | **0.71** |  |
| *AUC 0-60^1^* | 526 (474, 565) | 537 (457, 622) | 514 (444, 548) | 3.64 | 2 | 20 | 0.05 | 0.08 |  |  |  |  |
|  |  |  |  |  |  |  |  |  | AX vs INU | 0.28 | 0.34 |  |
|  |  |  |  |  |  |  |  |  | AX vs PLA | 0.16 | 0.45 |  |
|  |  |  |  |  |  |  |  |  | **INU vs PLA** | **0.02** | **0.82** |  |
| *AUC 60-180^1^* | 525 (486, 565) | 551 (466, 622) | 531 (437, 562) | 2.26 | 2 | 20 | 0.13 | 0.05 |  |  |  |  |
|  |  |  |  |  |  |  |  |  |  |  |  |  |
| **PYY** |  |  |  |  |  |  |  |  |  |  |  |  |
| *Fasting* | 145 (134, 183) | 152 (132, 162) | 147 (132, 160) | 0.78 | 2 | 20 | 0.47 | 0.02 |  |  |  |  |
| *After 120 minutes* | 179 (144, 225) | 185 (162, 211) | 187 (144, 201) | 0.5 | 2 | 20 | 0.61 | 0.01 |  |  |  |  |
| *Change* | 12 (-4, 54) | 33 (12, 55) | 20 (4, 45) | 0.54 | 2 | 20 | 0.59 | 0.02 |  |  |  |  |
|  |  |  |  |  |  |  |  |  |  |  |  |  |
| **Hunger and satiety** |  |  |  |  |  |  |  |  |  |  |  |  |
|  |  |  |  |  |  |  |  |  |  |  |  |  |
| **Hunger** |  |  |  |  |  |  |  |  |  |  |  |  |
| *Fasting* | 41 (12, 81) | 58 (23, 79) | 71 (18, 83) | 0.09 | 2 | 20 | 0.92 | 0.00 |  |  |  |  |
| *AUC 0-180* | 7,560 (2,715, 8,250) | 6,795 (1,710, 10,620) | 6,240 (2,295, 9,720) | 0.12 | 2 | 20 | 0.89 | 0.00 |  |  |  |  |
|  |  |  |  |  |  |  |  |  |  |  |  |  |
|  |  |  |  |  |  |  |  |  |  |  |  |  |
|  |  |  |  |  |  |  |  |  |  |  |  |  |
| *AUC 0-60* | 4,050 (1,395, 4,995) | 3,885 (600, 6,000) | 2,940 (1,155, 5,550) | 0.03 | 2 | 20 | 0.97 | 0.00 |  |  |  |  |
|  |  |  |  |  |  |  |  |  |  |  |  |  |
|  |  |  |  |  |  |  |  |  |  |  |  |  |
|  |  |  |  |  |  |  |  |  |  |  |  |  |
| *AUC 60-180* | 4,770 (2,250, 6,060) | 5,280 (1,140, 7,770) | 4,650 (1,830, 7,260) | 0.37 | 2 | 20 | 0.70 | 0.01 |  |  |  |  |
|  |  |  |  |  |  |  |  |  |  |  |  |  |
|  |  |  |  |  |  |  |  |  |  |  |  |  |
|  |  |  |  |  |  |  |  |  |  |  |  |  |
|  |  |  |  |  |  |  |  |  |  |  |  |  |
| **Satiety** |  |  |  |  |  |  |  |  |  |  |  |  |
| *Fasting^1^* | 31 (11, 51) | 34 (11, 75) | 21 (11, 73) | 0.05 | 2 | 20 | 0.95 | 0.00 |  |  |  |  |
| *AUC 0-180* | 8,700 (6,255, 10,035) | 9,030 (6,510, 14,460) | 9,285 (7,005, 12,690) | 1.23 | 2 | 20 | 0.31 | 0.03 |  |  |  |  |
| *AUC 0-60* | 6,090 (4,425, 8,055) | 6,990 (4,920, 9,855) | 7,515 (5,025, 9,555) | 1.51 | 2 | 20 | 0.25 | 0.04 |  |  |  |  |
| *AUC 60-180* | 5,610 (3,270, 7,080) | 5,790 (3,780, 9,720) | 5,160 (4,500, 7,320) | 0.96 | 2 | 20 | 0.40 | 0.03 |  |  |  |  |
|  |  |  |  |  |  |  |  |  |  |  |  |  |
| **Fullness** |  |  |  |  |  |  |  |  |  |  |  |  |
| *Fasting^1^* | 21 (5, 49) | 11 (1, 31) | 5 (1, 21) | 1.84 | 2 | 20 | 0.19 | 0.08 |  |  |  |  |
|  |  |  |  |  |  |  |  |  |  |  |  |  |
|  |  |  |  |  |  |  |  |  |  |  |  |  |
|  |  |  |  |  |  |  |  |  |  |  |  |  |
| *AUC 0-180* | 8,070 (4,275, 9,750) | 7,860 (4,155, 12,765) | 7,050 (3,375, 9,900) | 0.5 | 2 | 20 | 0.62 | 0.01 |  |  |  |  |
| *AUC 0-60* | 6,000 (2,865, 7,950) | 5,265 (3,525, 9,075) | 5,520 (3,030, 7,980) | 0.06 | 2 | 20 | 0.94 | 0.00 |  |  |  |  |
| *AUC 60-180* | 4,830 (3,000, 5,790) | 5,610 (2,010, 8,160) | 3,810 (2,550, 5,550) | 0.76 | 2 | 20 | 0.48 | 0.03 |  |  |  |  |
|  |  |  |  |  |  |  |  |  |  |  |  |  |
| **Prospective consumption** |  |  |  |  |  |  |  |  |  |  |  |  |
| *Fasting* | 65 (31, 81) | 71 (41, 93) | 85 (65, 94) | 1.84 | 2 | 20 | 0.18 | 0.07 |  |  |  |  |
| *AUC 0-180* | 8,880 (3,780, 11,145) | 8,640 (2,310, 11,955) | 6,975 (6,255, 12,750) | 0.38 | 2 | 20 | 0.69 | 0.01 |  |  |  |  |
|  |  |  |  |  |  |  |  |  |  |  |  |  |
|  |  |  |  |  |  |  |  |  |  |  |  |  |
|  |  |  |  |  |  |  |  |  |  |  |  |  |
| *AUC 0-60* | 5,550 (2,340, 6,900) | 4,965 (1,320, 6,855) | 3,915 (3,180, 7,350) | 0.09 | 2 | 20 | 0.92 | 0.00 |  |  |  |  |
| *AUC 60-180* | 6,450 (2,610, 7,650) | 6,240 (1,350, 8,790) | 5,130 (4,380, 8,730) | 0.34 | 2 | 20 | 0.71 | 0.01 |  |  |  |  |
|  |  |  |  |  |  |  |  |  |  |  |  |  |
|  |  |  |  |  |  |  |  |  |  |  |  |  |
|  |  |  |  |  |  |  |  |  |  |  |  |  |
|  |  |  |  |  |  |  |  |  |  |  |  |  |
| **Desire to eat** |  |  |  |  |  |  |  |  |  |  |  |  |
| *Fasting* | 54 (49, 111) | 73 (61, 98) | 84 (51, 111) | 0.46 | 2 | 20 | 0.64 | 0.01 |  |  |  |  |
| *AUC 0-180* | 8,550 (3,210, 11,070) | 7,245 (3,240, 10,185) | 6,225 (4,080, 10,755) | 0.01 | 2 | 20 | 0.99 | 0.00 |  |  |  |  |
|  |  |  |  |  |  |  |  |  |  |  |  |  |
|  |  |  |  |  |  |  |  |  |  |  |  |  |
|  |  |  |  |  |  |  |  |  |  |  |  |  |
| *AUC 0-60* | 4,050 (1,800, 6,225) | 3,780 (1,500, 6,255) | 2,745 (1,800, 6,045) | 0.04 | 2 | 20 | 0.96 | 0.00 |  |  |  |  |
| *AUC 60-180* | 6,660 (2,580, 7,500) | 5,490 (1,740, 7,620) | 5,010 (2,970, 8,580) | 0.01 | 2 | 20 | 0.99 | 0.00 |  |  |  |  |

| **P-types** | | | | | | | | | | |  |  |
| --- | --- | --- | --- | --- | --- | --- | --- | --- | --- | --- | --- | --- |
|  |  |  |  |  |  |  |  |  | *Pairwise post hoc test* | | | |
|  | ****AX**** | ****INU**** | ****PLA**** | **Statistic** | **df1** | **df** | **p_value** | **EffectSize_ANOVA** | **Comparison** | **Posthoc_pval** | **Cohens_d** |  |
|  | **N = 11** | **N = 11** | **N = 11** |  |  |  |  |  |  |  |  |  |
| **SCFAs and H2** | |  |  |  |  |  |  |  |  |  |  |  |
|  |  |  |  |  |  |  |  |  |  |  |  |  |
| **Acetate** |  |  |  |  |  |  |  |  |  |  |  |  |
| *Fasting* | 36 (2, 57) | 22 (4, 34) | 34 (6, 39) | 1.64 | 2 | 20 | 0.22 | 0.05 |  |  |  |  |
|  |  |  |  |  |  |  |  |  |  |  |  |  |
|  |  |  |  |  |  |  |  |  |  |  |  |  |
|  |  |  |  |  |  |  |  |  |  |  |  |  |
|  |  |  |  |  |  |  |  |  |  |  |  |  |
| *AUC 0-180* | 1,061 (73, 3,847) | 649 (116, 2,335) | 1,405 (969, 3,360) | 2.12 | 2 | 20 | 0.15 | 0.04 |  |  |  |  |
|  |  |  |  |  |  |  |  |  |  |  |  |  |
|  |  |  |  |  |  |  |  |  |  |  |  |  |
|  |  |  |  |  |  |  |  |  |  |  |  |  |
|  |  |  |  |  |  |  |  |  |  |  |  |  |
| *AUC 0-60* | 782 (73, 2,771) | 649 (112, 1,536) | 991 (849, 2,431) | 2.21 | 2 | 20 | 0.14 | 0.05 |  |  |  |  |
|  |  |  |  |  |  |  |  |  |  |  |  |  |
|  |  |  |  |  |  |  |  |  |  |  |  |  |
|  |  |  |  |  |  |  |  |  |  |  |  |  |
|  |  |  |  |  |  |  |  |  |  |  |  |  |
| *AUC 60-180* | 387 (0, 1,807) | 241 (0, 996) | 858 (178, 1,732) | 2.93 | 2 | 20 | 0.08 | 0.06 |  |  |  |  |
|  |  |  |  |  |  |  |  |  | AX vs INU | 0.19 | 0.42 |  |
|  |  |  |  |  |  |  |  |  | AX vs PLA | 0.48 | 0.22 |  |
|  |  |  |  |  |  |  |  |  | **INU vs PLA** | **0.02** | **0.86** |  |
|  |  |  |  |  |  |  |  |  |  |  |  |  |
|  |  |  |  |  |  |  |  |  |  |  |  |  |
| **Propionate** |  |  |  |  |  |  |  |  |  |  |  |  |
| *Fasting^1^* | 1.85 (1.55, 2.56) | 1.57 (1.14, 2.92) | 1.91 (0.60, 2.82) | 4.31 | 2 | 20 | 0.03 | 0.09 |  |  |  |  |
|  |  |  |  |  |  |  |  |  | AX vs INU | 0.05 | 0.67 |  |
|  |  |  |  |  |  |  |  |  | **AX vs PLA** | **0.04** | **0.71** |  |
|  |  |  |  |  |  |  |  |  | INU vs PLA | 0.14 | 0.48 |  |
|  |  |  |  |  |  |  |  |  |  |  |  |  |
| *AUC 0-180* | 347 (208, 425) | 296 (171, 367) | 295 (205, 425) | 1.2 | 2 | 20 | 0.32 | 0.01 |  |  |  |  |
|  |  |  |  |  |  |  |  |  |  |  |  |  |
|  |  |  |  |  |  |  |  |  |  |  |  |  |
|  |  |  |  |  |  |  |  |  |  |  |  |  |
|  |  |  |  |  |  |  |  |  |  |  |  |  |
| *AUC 0-60* | 241 (155, 307) | 194 (133, 273) | 209 (134, 296) | 1.57 | 2 | 20 | 0.23 | 0.01 |  |  |  |  |
|  |  |  |  |  |  |  |  |  |  |  |  |  |
|  |  |  |  |  |  |  |  |  |  |  |  |  |
|  |  |  |  |  |  |  |  |  |  |  |  |  |
|  |  |  |  |  |  |  |  |  |  |  |  |  |
| *AUC 60-180* | 217 (132, 258) | 164 (100, 210) | 160 (140, 263) | 0.83 | 2 | 20 | 0.45 | 0.01 |  |  |  |  |
|  |  |  |  |  |  |  |  |  |  |  |  |  |
| **Butyrate** |  |  |  |  |  |  |  |  |  |  |  |  |
| *Fasting* | 0.42 (0.19, 0.47) | 0.57 (0.17, 0.70) | 0.35 (0.22, 0.56) | 0.68 | 2 | 20 | 0.52 | 0.02 |  |  |  |  |
| *AUC 0-180^1^* | 85 (48, 101) | 78 (50, 109) | 74 (50, 112) | 0.51 | 2 | 20 | 0.61 | 0.01 |  |  |  |  |
| *AUC 0-60^1^* | 67 (37, 75) | 57 (39, 81) | 56 (35, 90) | 0.47 | 2 | 20 | 0.63 | 0.01 |  |  |  |  |
| *AUC 60-180^1^* | 47 (26, 61) | 43 (28, 62) | 45 (24, 62) | 0.45 | 2 | 20 | 0.64 | 0.01 |  |  |  |  |
|  |  |  |  |  |  |  |  |  |  |  |  |  |
| **Isobutyrate** |  |  |  |  |  |  |  |  |  |  |  |  |
| *Fasting* | 1.27 (0.92, 1.72) | 0.93 (0.62, 1.70) | 0.93 (0.51, 2.02) | 1.7 | 2 | 20 | 0.21 | 0.03 |  |  |  |  |
|  |  |  |  |  |  |  |  |  |  |  |  |  |
|  |  |  |  |  |  |  |  |  |  |  |  |  |
|  |  |  |  |  |  |  |  |  |  |  |  |  |
|  |  |  |  |  |  |  |  |  |  |  |  |  |
| *AUC 0-180* | 188 (173, 222) | 197 (118, 223) | 186 (137, 234) | 0.94 | 2 | 20 | 0.41 | 0.01 |  |  |  |  |
| *AUC 0-60* | 133 (112, 174) | 130 (93, 159) | 127 (94, 160) | 0.65 | 2 | 20 | 0.53 | 0.01 |  |  |  |  |
| *AUC 60-180* | 118 (108, 139) | 113 (63, 138) | 111 (92, 148) | 1.16 | 2 | 20 | 0.34 | 0.03 |  |  |  |  |
|  |  |  |  |  |  |  |  |  |  |  |  |  |
| **2-Methyl-butyrate** | |  |  |  |  |  |  |  |  |  |  |  |
| *Fasting^1^* | 1.83 (1.53, 2.92) | 2.46 (1.03, 2.61) | 1.56 (1.09, 2.92) | 1.62 | 2 | 20 | 0.22 | 0.04 |  |  |  |  |
|  |  |  |  |  |  |  |  |  |  |  |  |  |
|  |  |  |  |  |  |  |  |  |  |  |  |  |
|  |  |  |  |  |  |  |  |  |  |  |  |  |
|  |  |  |  |  |  |  |  |  |  |  |  |  |
| *AUC 0-180* | 347 (298, 483) | 362 (236, 437) | 349 (267, 406) | 0.05 | 2 | 20 | 0.95 | 0 |  |  |  |  |
| *AUC 0-60* | 233 (199, 300) | 254 (188, 333) | 264 (171, 281) | 0.03 | 2 | 20 | 0.97 | 0 |  |  |  |  |
| *AUC 60-180* | 224 (197, 310) | 223 (126, 284) | 215 (179, 274) | 0.1 | 2 | 20 | 0.91 | 0 |  |  |  |  |
|  |  |  |  |  |  |  |  |  |  |  |  |  |
| **Isovalerate** |  |  |  |  |  |  |  |  |  |  |  |  |
| *Fasting^1^* | 1.21 (0.87, 1.74) | 1.20 (0.77, 1.70) | 0.90 (0.55, 1.74) | 1.95 | 2 | 20 | 0.17 | 0.04 |  |  |  |  |
|  |  |  |  |  |  |  |  |  |  |  |  |  |
|  |  |  |  |  |  |  |  |  |  |  |  |  |
|  |  |  |  |  |  |  |  |  |  |  |  |  |
|  |  |  |  |  |  |  |  |  |  |  |  |  |
| *AUC 0-180^1^* | 219 (185, 296) | 198 (130, 276) | 212 (162, 270) | 0.22 | 2 | 20 | 0.8 | 0.01 |  |  |  |  |
| *AUC 0-60^1^* | 148 (133, 181) | 146 (93, 201) | 152 (109, 182) | 0.14 | 2 | 20 | 0.87 | 0 |  |  |  |  |
| *AUC 60-180^1^* | 147 (118, 192) | 123 (74, 207) | 137 (107, 180) | 0.2 | 2 | 20 | 0.82 | 0.01 |  |  |  |  |
|  |  |  |  |  |  |  |  |  |  |  |  |  |
| **Valerate** |  |  |  |  |  |  |  |  |  |  |  |  |
| *Fasting^1^* | 0.18 (0.09, 0.22) | 0.21 (0.03, 0.23) | 0.15 (0.08, 0.22) | 0.39 | 2 | 20 | 0.68 | 0.01 |  |  |  |  |
| *AUC 0-180* | 32 (19, 40) | 35 (15, 39) | 33 (15, 38) | 0.13 | 2 | 20 | 0.88 | 0 |  |  |  |  |
| *AUC 0-60* | 22 (11, 27) | 24 (10, 27) | 22 (10, 25) | 0 | 2 | 20 | 1 | 0 |  |  |  |  |
| *AUC 60-180* | 21 (13, 27) | 22 (10, 24) | 19 (11, 26) | 0.49 | 2 | 20 | 0.62 | 0.01 |  |  |  |  |
|  |  |  |  |  |  |  |  |  |  |  |  |  |
| **Caproic acid** |  |  |  |  |  |  |  |  |  |  |  |  |
| *Fasting^1^* | 0.88 (0.75, 1.28) | 0.81 (0.54, 1.03) | 0.96 (0.40, 1.47) | 2.17 | 2 | 20 | 0.14 | 0.06 |  |  |  |  |
| *AUC 0-180^1^* | 141 (120, 198) | 145 (90, 190) | 164 (113, 197) | 1.06 | 2 | 20 | 0.36 | 0.01 |  |  |  |  |
| *AUC 0-60^1^* | 94 (75, 127) | 107 (64, 117) | 113 (71, 134) | 0.28 | 2 | 20 | 0.76 | 0 |  |  |  |  |
| *AUC 60-180^1^* | 94 (83, 140) | 81 (55, 148) | 100 (79, 136) | 0.34 | 2 | 20 | 0.72 | 0 |  |  |  |  |
|  |  |  |  |  |  |  |  |  |  |  |  |  |
| **H2** |  |  |  |  |  |  |  |  |  |  |  |  |
| *Fasting^1^* | 7 (2, 14) | 12 (8, 22) | 4 (2, 28) | 1.39 | 2 | 20 | 0.27 | 0.08 |  |  |  |  |
| *AUC 0-180^1^* | 990 (419, 1,965) | 1,703 (720, 1,995) | 615 (300, 2,093) | 1.65 | 2 | 20 | 0.22 | 0.08 |  |  |  |  |
| *AUC 0-60^1^* | 473 (247, 1,013) | 938 (390, 1,283) | 240 (165, 1,560) | 1.62 | 2 | 20 | 0.22 | 0.08 |  |  |  |  |
| *AUC 60-180^1^* | 608 (215, 1,043) | 833 (420, 1,260) | 293 (180, 810) | 1.93 | 2 | 20 | 0.17 | 0.09 |  |  |  |  |
|  |  |  |  |  |  |  |  |  |  |  |  |  |
| **Metabolites** |  |  |  |  |  |  |  |  |  |  |  |  |
| **Glucose** |  |  |  |  |  |  |  |  |  |  |  |  |
| *Fasting* | 4.05 (3.89, 4.38) | 4.05 (3.89, 4.44) | 4.16 (3.72, 4.33) | 0.11 | 2 | 20 | 0.89 | 0 |  |  |  |  |
|  |  |  |  |  |  |  |  |  |  |  |  |  |
|  |  |  |  |  |  |  |  |  |  |  |  |  |
|  |  |  |  |  |  |  |  |  |  |  |  |  |
| *AUC 0-180^1^* | 605 (522, 664) | 608 (517, 731) | 599 (525, 711) | 0.09 | 2 | 20 | 0.92 | 0 |  |  |  |  |
| *AUC 0-60^1^* | 402 (372, 448) | 398 (345, 489) | 390 (347, 491) | 0.29 | 2 | 20 | 0.75 | 0.01 |  |  |  |  |
| *AUC 60-180^1^* | 368 (338, 428) | 388 (320, 446) | 370 (315, 426) | 0.03 | 2 | 20 | 0.97 | 0 |  |  |  |  |
|  |  |  |  |  |  |  |  |  |  |  |  |  |
| **Insulin** |  |  |  |  |  |  |  |  |  |  |  |  |
| *Fasting^1^* | 6.20 (4.40, 8.10) | 5.00 (3.60, 6.50) | 6.30 (4.60, 10.10) | 1.42 | 2 | 20 | 0.27 | 0.07 |  |  |  |  |
| *AUC 0-180^1^* | 4,280 (2,814, 5,381) | 4,839 (3,215, 5,778) | 4,293 (3,218, 4,811) | 0.14 | 2 | 20 | 0.87 | 0.01 |  |  |  |  |
| *AUC 0-60^1^* | 3,890 (2,283, 4,796) | 4,049 (2,570, 4,923) | 3,519 (2,690, 4,394) | 0.17 | 2 | 20 | 0.85 | 0.01 |  |  |  |  |
| *AUC 60-180^1^* | 1,680 (1,350, 2,214) | 2,238 (1,455, 2,856) | 2,082 (1,443, 2,835) | 0.23 | 2 | 20 | 0.8 | 0.01 |  |  |  |  |
|  |  |  |  |  |  |  |  |  |  |  |  |  |
| **Cholesterol** |  |  |  |  |  |  |  |  |  |  |  |  |
| *Fasting^1^* | 4.71 (4.42, 5.98) | 5.02 (4.29, 5.64) | 4.55 (4.29, 5.85) | 0.31 | 2 | 20 | 0.74 | 0.01 |  |  |  |  |
|  |  |  |  |  |  |  |  |  |  |  |  |  |
|  |  |  |  |  |  |  |  |  |  |  |  |  |
|  |  |  |  |  |  |  |  |  |  |  |  |  |
| *AUC 0-180^1^* | 875 (772, 972) | 851 (752, 1,006) | 823 (752, 1,041) | 0.24 | 2 | 20 | 0.79 | 0.01 |  |  |  |  |
|  |  |  |  |  |  |  |  |  |  |  |  |  |
|  |  |  |  |  |  |  |  |  |  |  |  |  |
|  |  |  |  |  |  |  |  |  |  |  |  |  |
| *AUC 0-60^1^* | 580 (520, 635) | 566 (509, 674) | 547 (505, 690) | 0.2 | 2 | 20 | 0.82 | 0 |  |  |  |  |
|  |  |  |  |  |  |  |  |  |  |  |  |  |
|  |  |  |  |  |  |  |  |  |  |  |  |  |
|  |  |  |  |  |  |  |  |  |  |  |  |  |
| *AUC 60-180^1^* | 587 (505, 654) | 574 (495, 669) | 538 (500, 709) | 0.27 | 2 | 20 | 0.77 | 0.01 |  |  |  |  |
|  |  |  |  |  |  |  |  |  |  |  |  |  |
| **PYY** |  |  |  |  |  |  |  |  |  |  |  |  |
| *Fasting* | 159 (128, 180) | 159 (121, 163) | 150 (122, 180) | 0.56 | 2 | 20 | 0.58 | 0 |  |  |  |  |
| *After 120 minutes* | 171 (141, 196) | 175 (146, 182) | 177 (142, 188) | 0.38 | 2 | 20 | 0.69 | 0.01 |  |  |  |  |
| *Change* | -2 (-8, 37) | 14 (1, 30) | 18 (-8, 42) | 0.24 | 2 | 20 | 0.79 | 0.01 |  |  |  |  |
|  |  |  |  |  |  |  |  |  |  |  |  |  |
| **Hunger and satiety** | |  |  |  |  |  |  |  |  |  |  |  |
|  |  |  |  |  |  |  |  |  |  |  |  |  |
| **Hunger** |  |  |  |  |  |  |  |  |  |  |  |  |
| *Fasting* | 38 (11, 66) | 51 (14, 78) | 19 (5, 81) | 2 | 2 | 20 | 0.16 | 0.04 |  |  |  |  |
| *AUC 0-180* | 1,890 (1,665, 6,525) | 3,915 (2,730, 9,105) | 4,575 (1,950, 7,980) | 5.91 | 2 | 20 | 0.01 | 0.06 |  |  |  |  |
|  |  |  |  |  |  |  |  |  | **AX vs INU** | **0.01** | **1.03** |  |
|  |  |  |  |  |  |  |  |  | **AX vs PLA** | **0.03** | **0.79** |  |
|  |  |  |  |  |  |  |  |  | INU vs PLA | 0.44 | 0.24 |  |
| *AUC 0-60* | 1,515 (675, 4,005) | 2,265 (1,230, 5,745) | 2,715 (1,200, 4,200) | 2.8 | 2 | 20 | 0.08 | 0.05 |  |  |  |  |
|  |  |  |  |  |  |  |  |  | **AX vs INU** | **0.03** | **0.75** |  |
|  |  |  |  |  |  |  |  |  | AX vs PLA | 0.07 | 0.6 |  |
|  |  |  |  |  |  |  |  |  | INU vs PLA | 0.8 | 0.08 |  |
| *AUC 60-180* | 1,770 (690, 4,680) | 2,880 (1,560, 6,120) | 2,760 (1,140, 6,180) | 7.93 | 2 | 20 | 0 | 0.06 |  |  |  |  |
|  |  |  |  |  |  |  |  |  | **AX vs INU** | **<0.01** | **1.16** |  |
|  |  |  |  |  |  |  |  |  | **AX vs PLA** | **0.01** | **0.91** |  |
|  |  |  |  |  |  |  |  |  | INU vs PLA | 0.34 | 0.3 |  |
|  |  |  |  |  |  |  |  |  |  |  |  |  |
| **Satiety** |  |  |  |  |  |  |  |  |  |  |  |  |
| *Fasting^1^* | 19 (13, 46) | 19 (6, 25) | 12 (2, 33) | 0.83 | 2 | 20 | 0.45 | 0.03 |  |  |  |  |
| *AUC 0-180* | 9,360 (5,835, 12,675) | 7,380 (5,580, 10,710) | 7,905 (5,640, 9,930) | 1.63 | 2 | 20 | 0.22 | 0.02 |  |  |  |  |
| *AUC 0-60* | 6,630 (4,185, 8,820) | 5,250 (3,405, 6,750) | 5,535 (4,200, 7,770) | 0.93 | 2 | 20 | 0.41 | 0.02 |  |  |  |  |
| *AUC 60-180* | 5,850 (3,660, 8,400) | 5,730 (3,750, 7,260) | 5,400 (3,180, 7,500) | 0.58 | 2 | 20 | 0.57 | 0.01 |  |  |  |  |
|  |  |  |  |  |  |  |  |  |  |  |  |  |
| **Fullness** |  |  |  |  |  |  |  |  |  |  |  |  |
| *Fasting^1^* | 18 (5, 54) | 7 (5, 18) | 7 (1, 31) | 4.15 | 2 | 20 | 0.03 | 0.13 |  |  |  |  |
|  |  |  |  |  |  |  |  |  | **AX vs INU** | **0.05** | **0.67** |  |
|  |  |  |  |  |  |  |  |  | **AX vs PLA** | **0.03** | **0.76** |  |
|  |  |  |  |  |  |  |  |  | INU vs PLA | 0.35 | 0.3 |  |
| *AUC 0-180* | 8,820 (3,450, 12,375) | 5,700 (2,445, 10,170) | 7,005 (3,330, 9,075) | 2.14 | 2 | 20 | 0.14 | 0.03 |  |  |  |  |
| *AUC 0-60* | 6,420 (2,310, 7,755) | 4,005 (2,115, 6,030) | 4,305 (2,670, 8,325) | 1.45 | 2 | 20 | 0.26 | 0.04 |  |  |  |  |
| *AUC 60-180* | 5,190 (2,430, 8,910) | 3,780 (1,410, 6,720) | 3,750 (1,620, 5,700) | 1.58 | 2 | 20 | 0.23 | 0.03 |  |  |  |  |
|  |  |  |  |  |  |  |  |  |  |  |  |  |
| **Prospective consumption** | |  |  |  |  |  |  |  |  |  |  |  |
| *Fasting* | 47 (19, 78) | 73 (39, 91) | 61 (28, 91) | 1.48 | 2 | 20 | 0.25 | 0.02 |  |  |  |  |
| *AUC 0-180* | 4,455 (2,625, 11,625) | 7,860 (3,195, 12,255) | 6,750 (2,955, 11,625) | 3.1 | 2 | 20 | 0.07 | 0.02 |  |  |  |  |
|  |  |  |  |  |  |  |  |  | AX vs INU | 0.08 | 0.58 |  |
|  |  |  |  |  |  |  |  |  | AX vs PLA | 0.34 | 0.3 |  |
|  |  |  |  |  |  |  |  |  | **INU vs PLA** | **0.03** | **0.75** |  |
| *AUC 0-60* | 2,805 (1,515, 6,885) | 4,320 (1,725, 7,755) | 4,140 (1,755, 7,050) | 1.34 | 2 | 20 | 0.28 | 0.01 |  |  |  |  |
| *AUC 60-180* | 3,150 (1,920, 8,400) | 5,910 (2,010, 8,310) | 4,680 (2,220, 8,220) | 5.32 | 2 | 20 | 0.01 | 0.02 |  |  |  |  |
|  |  |  |  |  |  |  |  |  | **AX vs INU** | **0.04** | **0.7** |  |
|  |  |  |  |  |  |  |  |  | AX vs PLA | 0.33 | 0.31 |  |
|  |  |  |  |  |  |  |  |  | **INU vs PLA** | **0.01** | **1.04** |  |
|  |  |  |  |  |  |  |  |  |  |  |  |  |
| **Desire to eat** |  |  |  |  |  |  |  |  |  |  |  |  |
| *Fasting* | 51 (25, 78) | 52 (24, 84) | 48 (24, 88) | 0.03 | 2 | 20 | 0.97 | 0 |  |  |  |  |
| *AUC 0-180* | 3,630 (1,005, 10,320) | 4,800 (2,685, 11,445) | 4,140 (2,460, 9,795) | 3.51 | 2 | 20 | 0.05 | 0.02 |  |  |  |  |
|  |  |  |  |  |  |  |  |  | AX vs INU | 0.05 | 0.66 |  |
|  |  |  |  |  |  |  |  |  | AX vs PLA | 0.58 | 0.17 |  |
|  |  |  |  |  |  |  |  |  | **INU vs PLA** | **<0.001** | **1.43** |  |
| *AUC 0-60* | 1,650 (855, 5,670) | 2,385 (1,155, 6,840) | 2,940 (1,350, 5,685) | 0.7 | 2 | 20 | 0.51 | 0.01 |  |  |  |  |
| *AUC 60-180* | 2,460 (420, 7,500) | 3,930 (1,740, 8,040) | 2,310 (1,050, 6,930) | 8.38 | 2 | 20 | <0.01 | 0.02 |  |  |  |  |
|  |  |  |  |  |  |  |  |  | **AX vs INU** | **0.01** | **1** |  |
|  |  |  |  |  |  |  |  |  | AX vs PLA | 0.18 | 0.43 |  |
|  |  |  |  |  |  |  |  |  | **INU vs PLA** | **0.01** | **1.05** |  |

Data are presented as mean (SD). Differences between treatments were assessed using repeated-measures ANOVA. If p < 0.1, post hoc pairwise comparisons were conducted using paired t-tests with least significant difference (LSD) adjustment. Effect sizes for post hoc comparisons are reported as Cohen’s d. A p-value < 0.05 was considered statistically significant. Abbreviations: ANOVA, analysis of variance; AX, arabinoxylan; B-type, *Bacteroides*-type; CID, clinical investigation day; df1/df2, numerator and denominator degrees of freedom; INU, inulin; N, number of participants; PLA, placebo; Posthoc_pval, adjusted post hoc p-value; P-type, *Prevotella*-type.

**Supplementary Table 6. Intervention effects on ordinal variables assessed using the Friedman test.**

| **B-types** | | | | | | | | | | | | | | | | | | | | | | |
| --- | --- | --- | --- | --- | --- | --- | --- | --- | --- | --- | --- | --- | --- | --- | --- | --- | --- | --- | --- | --- | --- | --- |
|  | ****AX**  N = 11** | | ****INU**  N = 11** | | ****PLA**  N = 11** | | **Statistic** | | **df** | **p_value** | | **EffectSize** | | **Comparison** | | **Posthoc_pval** | | **Z_value** | | **effsize_r** | |  |
| **Bristol stool chart** |  | |  | |  | | 0.56 | | 2 | 0.76 | | 0.03 | |  | |  | |  | |  | |  |
| 1 | 2 (18%) | | 0 (0%) | | 2 (18%) | |  | |  |  | |  | |  | |  | |  | |  | |  |
| 2 | 2 (18%) | | 1 (9.1%) | | 1 (9.1%) | |  | |  |  | |  | |  | |  | |  | |  | |  |
| 3 | 2 (18%) | | 1 (9.1%) | | 1 (9.1%) | |  | |  |  | |  | |  | |  | |  | |  | |  |
| 4 | 5 (45%) | | 9 (82%) | | 6 (55%) | |  | |  |  | |  | |  | |  | |  | |  | |  |
| 5 | 0 (0%) | | 0 (0%) | | 1 (9.1%) | |  | |  |  | |  | |  | |  | |  | |  | |  |
| 6 | 0 (0%) | | 0 (0%) | | 0 (0%) | |  | |  |  | |  | |  | |  | |  | |  | |  |
| 7 | 0 (0%) | | 0 (0%) | | 0 (0%) | |  | |  |  | |  | |  | |  | |  | |  | |  |
|  |  | |  | |  | |  | |  |  | |  | |  | |  | |  | |  | |  |
| **Stool frequency** |  | |  | |  | | 2.8 | | 2 | 0.25 | | 0.13 | |  | |  | |  | |  | |  |
| Several times a day | 1 (9.1%) | | 4 (36%) | | 2 (18%) | |  | |  |  | |  | |  | |  | |  | |  | |  |
| Once a day | 9 (82%) | | 7 (64%) | | 9 (82%) | |  | |  |  | |  | |  | |  | |  | |  | |  |
| Every 2 days | 0 (0%) | | 0 (0%) | | 0 (0%) | |  | |  |  | |  | |  | |  | |  | |  | |  |
| 2-3 times per week | 1 (9.1%) | | 0 (0%) | | 0 (0%) | |  | |  |  | |  | |  | |  | |  | |  | |  |
| Once a week | 0 (0%) | | 0 (0%) | | 0 (0%) | |  | |  |  | |  | |  | |  | |  | |  | |  |
| Less than one a week | 0 (0%) | | 0 (0%) | | 0 (0%) | |  | |  |  | |  | |  | |  | |  | |  | |  |
| **P-type** | | | | | | | | | | | | | | | | | | | | |  |  |
|  | ****AX**  N = 11** | ****INU**  N = 11** | | ****PLA**  N = 11** | | **Statistic** | | **df** | **p_value** | | **EffectSize** | | **Comparison** | | **Posthoc_pval** | | **Z_value** | | **effsize_r** | |  |  |
| **Bristol stool chart** | |  | |  | | 3.63 | | 2 | 0.16 | | 0.16 | |  | |  | |  | |  | |  |  |
| 1 | 1 (9.1%) | 2 (18%) | | 1 (9.1%) | |  | |  |  | |  | |  | |  | |  | |  | |  |  |
| 2 | 0 (0%) | 0 (0%) | | 1 (9.1%) | |  | |  |  | |  | |  | |  | |  | |  | |  |  |
| 3 | 0 (0%) | 0 (0%) | | 2 (18%) | |  | |  |  | |  | |  | |  | |  | |  | |  |  |
| 4 | 8 (73%) | 7 (64%) | | 5 (45%) | |  | |  |  | |  | |  | |  | |  | |  | |  |  |
| 5 | 0 (0%) | 1 (9.1%) | | 1 (9.1%) | |  | |  |  | |  | |  | |  | |  | |  | |  |  |
| 6 | 2 (18%) | 1 (9.1%) | | 1 (9.1%) | |  | |  |  | |  | |  | |  | |  | |  | |  |  |
| 7 | 0 (0%) | 0 (0%) | | 0 (0%) | |  | |  |  | |  | |  | |  | |  | |  | |  |  |
|  |  |  | |  | |  | |  |  | |  | |  | |  | |  | |  | |  |  |
| **Stool frequency** | |  | |  | | 6.00 | | 2 | 0.05 | | 0.27 | |  | |  | |  | |  | |  |  |
| Several times a day | 3 (27%) | 5 (45%) | | 4 (36%) | |  | |  |  | | 0.35 | | INU vs AX | | 0.24 | | 1.16 | | 0.35 | |  |  |
| Once a day | 8 (73%) | 5 (45%) | | 4 (36%) | |  | |  |  | | 0.16 | | PLA vs AX | | 0.60 | | 0.53 | | 0.16 | |  |  |
| Every 2 days | 0 (0%) | 1 (9.1%) | | 3 (27%) | |  | |  |  | | 0.16 | | PLA vs INU | | 0.60 | | 0.53 | | 0.16 | |  |  |
| 2-3 times per week | 0 (0%) | 0 (0%) | | 0 (0%) | |  | |  |  | |  | |  | |  | |  | |  | |  |  |
| Once a week | 0 (0%) | 0 (0%) | | 0 (0%) | |  | |  |  | |  | |  | |  | |  | |  | |  |  |
| Less than one a week | 0 (0%) | 0 (0%) | | 0 (0%) | |  | |  |  | |  | |  | |  | |  | |  | |  |  |

Data are presented as n (%). Differences between treatments were assessed using the Friedman test, with Kendall’s W reported as the effect size. For p < 0.1, post hoc pairwise tests were conducted using the Eisinger method with least significant difference (LSD) adjustment. A p-value < 0.05 was considered statistically significant. Effect sizes are reported as r. Abbreviations: AX, arabinoxylan; B-type, *Bacteroides*-type; df, degrees of freedom; EffectSize, effect size; effsize_r, effect size r; INU, inulin; N, number of participants; PLA, placebo; Posthoc_pval, adjusted post hoc p-value; P-type, *Prevotella*-type; Z_value, standardized test statistic.

**Supplementary Table 7: Impact of AX, INU and PLA on microbiota alpha diversity in B- and P-Types .**

| **B-types** | | | | | | | | | | | | |
| --- | --- | --- | --- | --- | --- | --- | --- | --- | --- | --- | --- | --- |
|  |  |  |  | *Pairwise post hoc test* | | | | | | | |  |
|  | ****AX**  N = 11** | ****INU**  N = 11** | ****PLA**  N = 11** | **Statistic** | **df1** | **df2** | **p_value** | **EffectSize_ANOVA** | **Comparison** | **Posthoc_pval** | Cohens_d |  |
| **Shannon diversity** | 4.10 (3.62, 4.17) | 3.84 (3.68, 4.17) | 4.12 (4.02, 4.29) | 4.94 | 2 | 20 | 0.02 | 0.10 |  |  |  |  |
|  |  |  |  |  |  |  |  |  | PLA vs AX | 0.05 | 0.66 |  |
|  |  |  |  |  |  |  |  |  | **PLA vs INU** | **0.02** | **0.88** |  |
|  |  |  |  |  |  |  |  |  | AX vs INU | 0.27 | 0.35 |  |
| **Chao richness^1^** | 139.08 (111.55, 198.02) | 117.43 (97.49, 175.14) | 149.25 (119.04, 238.78) | 3.18 | 2 | 20 | 0.06 | 0.02 |  |  |  |  |
|  |  |  |  |  |  |  |  |  | PLA vs AX | 0.18 | 0.43 |  |
|  |  |  |  |  |  |  |  |  | **PLA vs INU** | **0.04** | **0.69** |  |
|  |  |  |  |  |  |  |  |  | AX vs INU | 0.26 | 0.36 |  |

| **P-types** | | | | | | | | | | | |
| --- | --- | --- | --- | --- | --- | --- | --- | --- | --- | --- | --- |
|  |  |  |  | *Pairwise post hoc test* | | | | | | | |
|  | ****AX**  N = 11** | ****INU**  N = 11** | ****PLA**  N = 11** | **Statistic** | **df1** | **df2** | **p_value** | **EffectSize_ANOVA** | **Comparison** | **Posthoc_pval** | Cohens_d |
| **Shannon diversity** | 4.32 (3.91,4.45) | 4.11 (4.04, 4.43) | 4.42 (4.19,4.61) | 4.17 | 2 | 20 | 0.03 | 0.09 |  |  |  |
|  |  |  |  |  |  |  |  |  | PLA vs AX | 0.05 | 0.66 |
|  |  |  |  |  |  |  |  |  | **PLA vs INU** | **0.03** | **0.79** |
|  |  |  |  |  |  |  |  |  | AX vs INU | 0.56 | 0.18 |
| **Chao richness^1^** | 173.62 (160.24, 264.61) | 150.00 (135.52, 300.89) | 183.67 (159,00, 247.61) | 0.5 | 2 | 20 | 0.62 | 0.00 |  |  |  |

| Data are presented as median (Q1, Q3). Differences between treatments were assessed using repeated-measures ANOVA. If p < 0.1, post hoc pairwise comparisons were conducted using paired t-tests and LSD adjustment. Effect sizes for post hoc comparisons are reported as Cohen’s d. A p-value < 0.05 was considered statistically significant. ¹log-transformed prior to statistical analysis to achieve normal distribution. Abbreviations: ANOVA, analysis of variance; AX, arabinoxylan; B-type, *Bacteroides*-type; df1/df2, numerator and denominator degrees of freedom; INU, inulin; N, number of participants; PLA, placebo; Posthoc_pval, adjusted post hoc p-value; P-type, *Prevotella*-type. |  |
| --- | --- |
|  |  |

**Supplementary Table 8: Impact of AX, INU microbiota beta diversity in B- and P-Types compared to PLA.**

| **B-types** | | | | | | | |
| --- | --- | --- | --- | --- | --- | --- | --- |
|  |  |  |  | *Pairwise post hoc test* | |  | |
|  | ****AX**  N = 11** | ****INU**  N = 11** | **Estimate** | **Std.Error** | **Statistic** | | **P.Value** |
| **Bray curtis distance** | 0.38 (0.32, 0.46) | 0.37 (0.31, 0.41) | -0.01 | 0.04 | -0.29 | | 0.78 |
|  |  |  |  |  |  | |  |
| **P-types** | | | | | | | |
|  |  |  |  | *Pairwise post hoc test* | |  | |
|  | ****AX**  N = 11** | ****INU**  N = 11** | **Estimate** | **Std.Error** | **Statistic** | | **P.Value** |
| **Bray curtis distance** | 0.42 (0.37,0.46) | 0.43 (0.34, 0.54) | 0.02 | 0.03 | 0.78 | | 0.45 |

Data are presented as median (Q1, Q3). Beta diversity analysis was conducted using a linear mixed-effects model, with Bray–Curtis distance as the dependent variable. The analysis was performed using the generate_beta_trend_long() function. Treatment was included as the independent variable, while subject ID was modeled as a random effect. A p-value < 0.05 was considered statistically significant. Abbreviations: AX, arabinoxylan; B-type, *Bacteroides*-type; Estimate, model coefficient estimate; INU, inulin; N, number of participants; P-type, *Prevotella*-type; PLA, placebo; Statistic, test statistic; Std.Error, standard error.

**Supplementary Table 9. Differential abundant genera after AX, INU, PLA intervention.**

| **B-types** | | | | | | | | |  |
| --- | --- | --- | --- | --- | --- | --- | --- | --- | --- |
| **feature** | **metadata** | **value** | **coef** | **stderr** | **N** | **N.not.0** | **pval** | **qval** | |
| **Anaerostipes** | treatment | INU | 0.09 | 0.02 | 33.00 | 33.00 | 0.00 | 0.01 | |
| **Ruminococcus** | treatment | INU | -0.07 | 0.02 | 33.00 | 33.00 | 0.00 | 0.01 | |
| **Bifidobacterium** | treatment | INU | 0.15 | 0.05 | 33.00 | 31.00 | 0.01 | 0.17 | |
| **Fusicatenibacter** | treatment | AX | 0.05 | 0.02 | 33.00 | 33.00 | 0.01 | 0.18 | |
| **Faecalibacillus** | treatment | AX | -0.02 | 0.01 | 33.00 | 29.00 | 0.01 | 0.18 | |
| **Phocaeicola** | treatment | INU | -0.06 | 0.02 | 33.00 | 33.00 | 0.01 | 0.18 | |
| **P-types** | | | | | | | | |  |
| **feature** | **metadata** | **value** | **coef** | **stderr** | **N** | **N.not.0** | **pval** | **qval** | |
| **Anaerostipes** | treatment | INU | 0.13 | 0.02 | 33.00 | 33.00 | 0.00 | 0.00 | |
| **Bifidobacterium** | treatment | INU | 0.09 | 0.02 | 33.00 | 30.00 | 0.00 | 0.03 | |
| **Phocaeicola** | treatment | AX | -0.07 | 0.02 | 33.00 | 33.00 | 0.00 | 0.03 | |
| **Phocaeicola** | treatment | INU | -0.07 | 0.02 | 33.00 | 33.00 | 0.00 | 0.03 | |
| **Collinsella** | treatment | INU | 0.03 | 0.01 | 33.00 | 33.00 | 0.00 | 0.07 | |
| **Alistipes** | treatment | AX | -0.05 | 0.02 | 33.00 | 30.00 | 0.01 | 0.16 | |
| **Clostridiales.Order** | treatment | AX | -0.07 | 0.03 | 33.00 | 32.00 | 0.02 | 0.17 | |
| **Coprococcus** | treatment | INU | -0.03 | 0.01 | 33.00 | 33.00 | 0.02 | 0.17 | |
| **Lachnospira** | treatment | INU | -0.05 | 0.02 | 33.00 | 31.00 | 0.02 | 0.17 | |
| **Paraprevotella** | treatment | AX | 0.03 | 0.01 | 33.00 | 17.00 | 0.02 | 0.17 | |
| **Romboutsia** | treatment | AX | -0.04 | 0.01 | 33.00 | 28.00 | 0.02 | 0.17 | |

Differential abundant taxa were assessed using linear mixed models in MaAsLin2. Genera were filtered for ≥10% prevalence and ≥1% abundance. Statistical significance was determined using the Benjamini–Hochberg method for multiple comparison adjustment, with a target rate of 0.25 for q-values. Abbreviations: AX, arabinoxylan; B-type, *Bacteroides*-type; coef, coefficient; INU, inulin; N, number of participants; N.not.0, number of non-zero observations; PLA, placebo; P-type, *Prevotella*-type; qval, adjusted p-value; stderr, standard error.

**Supplementary Table 11: Impact of AX, INU, and PLA on microbial network metrics in B- and P-Types.**

| **B-types** | | | | | | | | | | | | | | | | | | | | | |
| --- | --- | --- | --- | --- | --- | --- | --- | --- | --- | --- | --- | --- | --- | --- | --- | --- | --- | --- | --- | --- | --- |
|  | |  |  | |  | | *Pairwise post hoc test* | | | | | | | | | | | | | | |
|  | | ****PLA**  N = 11** | ****AX**  N = 11** | | ****INU**  N = 11** | | **Statistic** | | **df1** | | **df2** | | **p_value** | | **EffectSize_ANOVA** | | **Comparison** | | **Posthoc_pval** | | Cohens_d |
| **Degree** | | 0.75 (0.66, 1.01) | 0.73 (0.64, 0.89) | | 0.71 (0.64, 0.78) | | 1.67 | | 2 | | 20 | | 0.21 | | 0.14 | |  | |  | |  |
| **Closeness** | | 0.48 (0.37, 0.51) | 0.41 (0.34, 0.48) | | 0.52 (0.37, 0.67) | | 2.43 | | 2 | | 20 | | 0.11 | | 0.20 | |  | |  | |  |
| **Betweenness** | | **0.53 (0.28, 0.80)** | **2.16 (0.35, 2.73)** | | **0.61 (0.31, 1.76)** | | **6.11** | | **2** | | **20** | | **0.01** | | **0.38** | |  | |  | |  |
|  | |  |  | |  | |  | |  | |  | |  | |  | | **PLA vs AX** | | **0.02** | | **0.84** |
|  | |  |  | |  | |  | |  | |  | |  | |  | | PLA vs INU | | 0.10 | | 0.54 |
|  | |  |  | |  | |  | |  | |  | |  | |  | | AX vs INU | | 0.06 | | 0.65 |
| **Eigen** | | 3.26 (2.99, 4.32) | 3.57 (2.98, 4.06) | | 3.54 (3.27, 4.80) | | 1 | | 2 | | 20 | | 0.39 | | 0.09 | |  | |  | |  |
| **P-types** | | | | | | | | | | | | | | | | | | | | | |
|  |  | | |  | |  | | *Pairwise post hoc test* | | | | | | | | | | | | | |
|  | ****PLA**  N = 11** | | | ****AX**  N = 11** | | ****INU**  N = 11** | | **Statistic** | | **df1** | | **df2** | | **p_value** | | **EffectSize_ANOVA** | | **Comparison** | | **Posthoc_pval** | Cohens_d |
| **Degree** | 0.78 (0.68, 0.89) | | | 0.80 (0.66, 0.96) | | 0.78 (0.64, 0.87) | | 0.08 | | 2 | | 20 | | 0.92 | | 0.01 | |  | |  |  |
| **Closeness** | **0.47 (0.38, 0.55)** | | | **0.34 (0.32, 0.39)** | | **0.49 (0.40, 0.51)** | | **8.8** | | **2** | | **30** | | **<0.01** | | **0.37** | |  | |  |  |
|  |  | | |  | |  | |  | |  | |  | |  | |  | | **PLA vs AX** | | **0.01** | **0.93** |
|  |  | | |  | |  | |  | |  | |  | |  | |  | | PLA vs INU | | 0.67 | 0.13 |
|  |  | | |  | |  | |  | |  | |  | |  | |  | | **AX vs INU** | | **<0.01** | **1.16** |
|  |  | | |  | |  | |  | |  | |  | |  | |  | |  | |  |  |
| **Betweenness** | 0.61 (0.44, 2.36) | | | 1.87 (1.65, 4.05) | | 1.29 (1.01, 1.98) | | 3.7 | | 2 | | 30 | | 0.04 | | 0.20 | |  | |  |  |
|  |  | | |  | |  | |  | |  | |  | |  | |  | | PLA vs AX | | 0.10 | 0.56 |
|  |  | | |  | |  | |  | |  | |  | |  | |  | | PLA vs INU | | 0.76 | 0.10 |
|  |  | | |  | |  | |  | |  | |  | |  | |  | | AX vs INU | | 0.06 | 0.65 |
|  |  | | |  | |  | |  | |  | |  | |  | |  | |  | |  |  |
| **Eigen** | 3.75 (2.63, 4.54) | | | 4.13 (3.49, 4.90) | | 4.24 (3.84, 4.95) | | 3.5 | | 2 | | 20 | | 0.05 | | 0.26 | |  | |  |  |
|  |  | | |  | |  | |  | |  | |  | |  | |  | | PLA vs AX | | 0.06 | 0.65 |
|  |  | | |  | |  | |  | |  | |  | |  | |  | | PLA vs INU | | 0.05 | 0.67 |
|  |  | | |  | |  | |  | |  | |  | |  | |  | | AX vs INU | | 0.93 | 0.03 |

Data are presented as median (Q1, Q3). Differences between treatments were assessed using repeated-measures ANOVA. If p < 0.1, post hoc pairwise comparisons were conducted using paired t-tests and LSD adjustment. Effect sizes for post hoc comparisons are reported as Cohen’s d. A p-value < 0.05 was considered statistically significant. Abbreviations: ANOVA, analysis of variance; AX, arabinoxylan; B-type, *Bacteroides*-type; Cohen’s d, standardized mean difference; df1/df2, numerator and denominator degrees of freedom; Eigen, eigenvector centrality; INU, inulin; N, number of participants; PLA, placebo; Posthoc_pval, adjusted post hoc p-value; P-type, *Prevotella*-type.

**Supplementary Table 11: Adjacency matrices of genus-level microbial co-occurrence networks in B-type individuals across PLA, AX, and INU intervention.**

| **Network** | **From** | **To** | **Weight** |
| --- | --- | --- | --- |
| PLA – B-type | Butyricimonas | Clostridium_XVIII | 0.10 |
| PLA – B-type | Catabacter | Lactococcus | 0.10 |
| PLA – B-type | Oscillibacter | Parabacteroides | 0.09 |
| PLA – B-type | Butyricicoccus | Catabacter | 0.07 |
| PLA – B-type | Anaerobutyricum | Bacteroides | 0.06 |
| PLA – B-type | Olsenella | Veillonella | 0.06 |
| PLA – B-type | Bacteroides | Phocaeicola | 0.05 |
| PLA – B-type | Clostridium_IV | Escherichia/Shigella | -0.05 |
| PLA – B-type | Faecalibacterium | Fusicatenibacter | 0.04 |
| PLA – B-type | Anaerobutyricum | Fusicatenibacter | 0.04 |
| PLA – B-type | Bacteroides | Blautia | 0.04 |
| PLA – B-type | Anaerostipes | Fusicatenibacter | 0.03 |
| PLA – B-type | Anaerobutyricum | Faecalibacterium | 0.03 |
| PLA – B-type | Barnesiella | Lachnospira | -0.03 |
| PLA – B-type | Anaeromassilibacillus | Lactococcus | 0.02 |
| AX – B-type | Agathobaculum | Blautia | 0.08 |
| AX – B-type | Bifidobacterium | Phocaeicola | 0.08 |
| AX – B-type | Lactococcus | Prevotella | 0.08 |
| AX – B-type | Haemophilus | Schaalia | 0.07 |
| AX – B-type | Lachnospira | Schaalia | 0.07 |
| AX – B-type | Anaeromassilibacillus | Mediterraneibacter | -0.06 |
| AX – B-type | Catabacter | Mailhella | 0.04 |
| AX – B-type | Haemophilus | Veillonella | 0.03 |
| AX – B-type | Bifidobacterium | Roseburia | 0.03 |
| AX – B-type | Faecalibacterium | Fusicatenibacter | 0.02 |
| INU – B-type | Haemophilus | Schaalia | 0.11 |
| INU – B-type | Aminipila | Butyricimonas | 0.10 |
| INU – B-type | Coprococcus | Gemmiger | 0.10 |
| INU – B-type | Kineothrix | Mediterraneibacter | 0.09 |
| INU – B-type | Anaeromassilibacillus | Ruthenibacterium | 0.08 |
| INU – B-type | Bacteroides | Fusicatenibacter | 0.08 |
| INU – B-type | Anaerobutyricum | Blautia | 0.05 |
| INU – B-type | Bacteroides | Clostridium_IV | -0.04 |
| INU – B-type | Akkermansia | Harryflintia | -0.04 |
| INU – B-type | Fusicatenibacter | Phocaeicola | 0.04 |
| INU – B-type | Akkermansia | Victivallis | 0.03 |
| INU – B-type | Clostridium_IV | Faecalibacterium | -0.03 |
| INU – B-type | Odoribacter | Oscillibacter | 0.02 |

Abbreviations: AX, arabinoxylan; B-type, *Bacteroides*-type; INU, inulin; PLA, placebo.

**Supplementary Table 12: Adjacency matrices of genus-level microbial co-occurrence networks in P-type individuals across PLA, AX, and INU intervention.**

| **Network** | **From** | **To** | **Weight** |
| --- | --- | --- | --- |
| PLA – P-type | Faecalibacterium | Lachnospira | 0.11 |
| PLA – P-type | Anaerobutyricum | Dorea | 0.10 |
| PLA – P-type | Dialister | Phascolarctobacterium | -0.08 |
| PLA – P-type | Anaerostipes | Dorea | 0.07 |
| PLA – P-type | Anaerostipes | Blautia | 0.06 |
| PLA – P-type | Blautia | Faecalibacillus | 0.05 |
| PLA – P-type | Blautia | Dorea | 0.05 |
| PLA – P-type | Blautia | Lachnospira | 0.05 |
| PLA – P-type | Coprococcus | Faecalibacterium | 0.03 |
| PLA – P-type | Anaerobutyricum | Anaerostipes | 0.03 |
| PLA – P-type | Faecalibacillus | Fusicatenibacter | 0.03 |
| PLA – P-type | Anaerobutyricum | Blautia | 0.02 |
| PLA – P-type | Faecalibacterium | Turicibacter | -0.02 |
| AX – P-type | Dialister | Phascolarctobacterium | -0.08 |
| AX – P-type | Blautia | Dorea | 0.06 |
| AX – P-type | Enterocloster | Faecalibacterium | 0.06 |
| AX – P-type | Anaerostipes | Neglecta | 0.04 |
| AX – P-type | Agathobaculum | Dorea | 0.03 |
| AX – P-type | Faecalibacterium | Prevotella | 0.03 |
| AX – P-type | Anaerobutyricum | Faecalibacterium | 0.02 |
| AX – P-type | Anaerostipes | Faecalibacillus | 0.02 |
| INU – P-type | Anaeromassilibacillus | Holdemania | 0.11 |
| INU – P-type | Agathobaculum | Anaerobutyricum | 0.09 |
| INU – P-type | Faecalibacillus | Faecalibacterium | 0.09 |
| INU – P-type | Faecalibacillus | Lachnospira | 0.07 |
| INU – P-type | Faecalibacterium | Lachnospira | 0.07 |
| INU – P-type | Anaerobutyricum | Faecalibacillus | 0.05 |
| INU – P-type | Faecalibacillus | Mediterraneibacter | 0.05 |
| INU – P-type | Dialister | Phascolarctobacterium | -0.04 |
| INU – P-type | Lawsonibacter | Vampirovibrio | 0.04 |
| INU – P-type | Mailhella | Ruthenibacterium | 0.04 |
| INU – P-type | Phocaeicola | Slackia | -0.04 |
| INU – P-type | Adlercreutzia | Monoglobus | 0.03 |
| INU – P-type | Blautia | Coprococcus | 0.03 |
| INU – P-type | Faecalibacterium | Mediterraneibacter | 0.03 |
| INU – P-type | Caproicibacter | Harryflintia | 0.03 |
| INU – P-type | Dysosmobacter | Neglecta | 0.03 |

Abbreviations: AX, arabinoxylan; INU, inulin; PLA, placebo; P-type, *Prevotella*-type.

**Supplementary Table 13. Associations between microbial genera with SCFA concentrations.**

| **B-types** | | | | | | | |
| --- | --- | --- | --- | --- | --- | --- | --- |
| **feature** | **value** | **coef** | **stderr** | **N** | **N.not.0** | **pval** | **qval** |
| **Fusicatenibacter** | acetate_auc_0_180 | 0.04 | 0.01 | 33.0 | 33.0 | <0.01 | <0.01 |
|  |  |  |  |  |  |  |  |
| **Clostridium_IV** | propionate_auc_0_60 | 0.03 | 0.01 | 33.00 | 23.00 | <0.01 | 0.02 |
| **Oscillibacter** | propionate_auc_0_60 | 0.02 | 0.01 | 33.00 | 27.00 | 0.01 | 0.13 |
| **Fusicatenibacter** | propionate_auc_0_60 | 0.03 | 0.01 | 33.00 | 33.00 | 0.01 | 0.16 |
| **Dialister** | propionate_auc_0_60 | 0.02 | 0.01 | 33.00 | 24.00 | 0.02 | 0.16 |
|  |  |  |  |  |  |  |  |
| **Parabacteroides** | isobutyrate_fasting | 0.04 | 0.01 | 33.0 | 29.0 | 0.00 | 0.01 |
| **Bacteroides** | isobutyrate_fasting | 0.10 | 0.03 | 33.0 | 33.0 | 0.00 | 0.02 |
| **Phocaeicola** | isobutyrate_fasting | 0.08 | 0.03 | 33.0 | 33.0 | 0.01 | 0.16 |
| **Fusicatenibacter** | isobutyrate_fasting | 0.05 | 0.02 | 33.0 | 33.0 | 0.02 | 0.17 |
| **Agathobacter** | isobutyrate_fasting | -0.07 | 0.03 | 33.0 | 26.0 | 0.04 | 0.19 |
| **Anaerostipes** | isobutyrate_fasting | -0.07 | 0.03 | 33.0 | 33.0 | 0.03 | 0.19 |
| **Bifidobacterium** | isobutyrate_fasting | -0.13 | 0.06 | 33.0 | 31.0 | 0.04 | 0.19 |
| **Clostridium_IV** | isobutyrate_fasting | 0.03 | 0.01 | 33.0 | 23.0 | 0.05 | 0.19 |
| **Faecalibacillus** | isobutyrate_fasting | -0.03 | 0.01 | 33.0 | 29.0 | 0.04 | 0.19 |
| **Prevotella** | isobutyrate_fasting | -0.02 | 0.01 | 33.0 | 10.0 | 0.04 | 0.19 |
| **Coprococcus** | isobutyrate_fasting | 0.03 | 0.01 | 33.0 | 32.0 | 0.06 | 0.22 |
|  |  |  |  |  |  |  |  |
| **Bacteroides** | X2_mebutyrate_fasting | 0.04 | 0.01 | 33.0 | 33.0 | 0.00 | 0.02 |
| **Parabacteroides** | X2_mebutyrate_fasting | 0.01 | 0.00 | 33.0 | 29.0 | 0.00 | 0.08 |
|  |  |  |  |  |  |  |  |
| **Bacteroides** | isovalerate_fasting | 0.04 | 0.02 | 33.0 | 33.0 | 0.01 | 0.23 |
| **Parabacteroides** | isovalerate_fasting | 0.02 | 0.01 | 33.0 | 29.0 | 0.01 | 0.23 |
| **P-types** | | | | | | | |
| **Paraprevotella** | propionate_fasting | 0.04 | 0.01 | 33.00 | 17.00 | <0.01 | 0.02 |

Differential abundant taxa were assessed using linear mixed models in MaAsLin2. Genera were filtered for ≥10% prevalence and ≥1% abundance. Statistical significance was determined using the Benjamini–Hochberg method for multiple comparison adjustment, with a target rate of 0.25 for q-values. Abbreviations: AX, arabinoxylan; B-type, *Bacteroides*-type; coef, coefficient; INU, inulin; N, number of participants; N.not.0, number of non-zero observations; PLA, placebo; P-type, *Prevotella*-type; qval, adjusted p-value; SCFA, short-chain fatty acids; stderr, standard error.


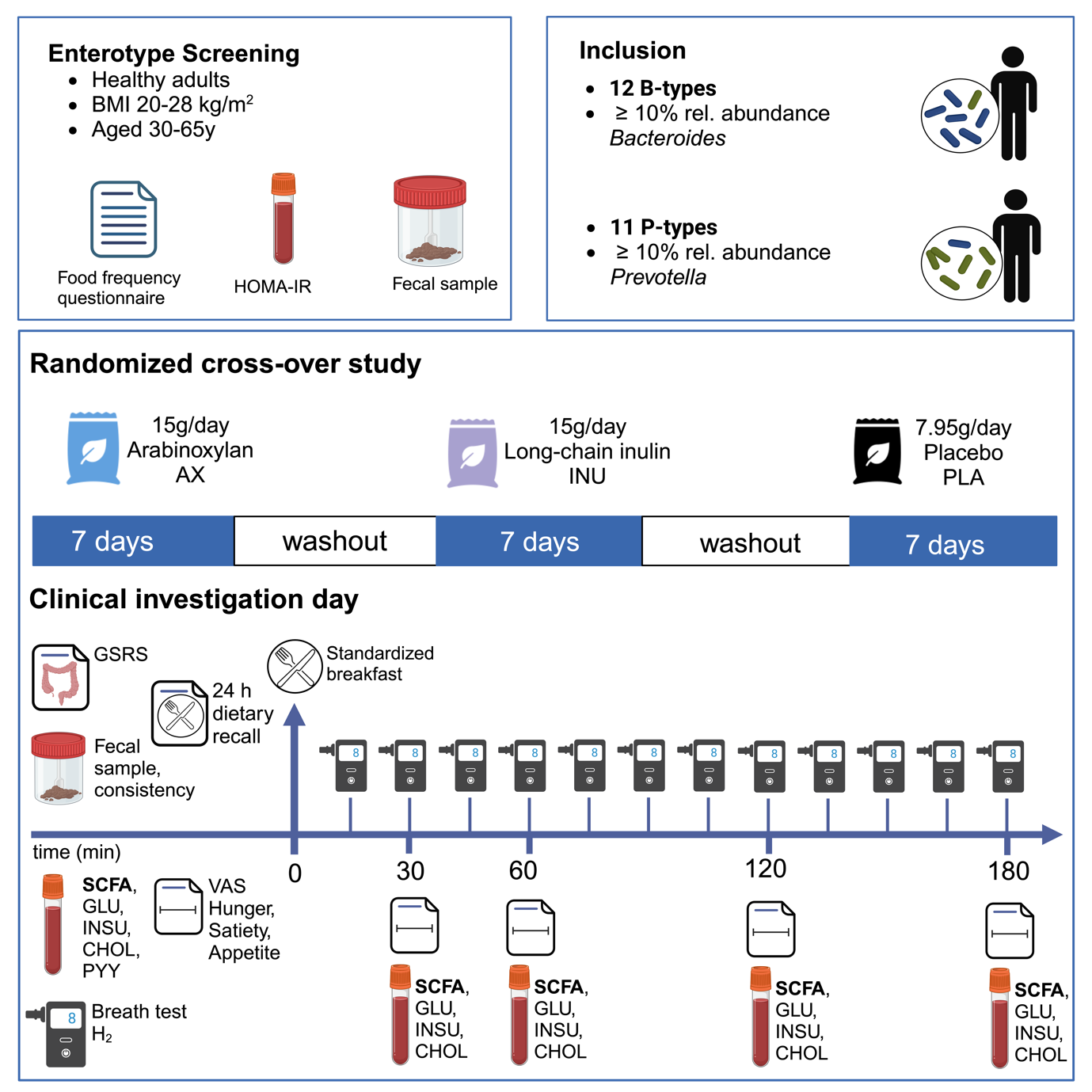


**Supplementary Figure 1.** Study design, inclusion criteria, and clinical investigation day protocol**.** Participants were stratified into enterotypes based on fecal microbiota composition (≥10% relative abundance of *Bacteroides* or *Prevotella*). The study followed a randomized cross-over design with three supplementation periods of 7 days each (AX, INU, PLA), separated by 14-day washout phases. AX, arabinoxylan; B-type, *Bacteroides*-dominant individuals; CHOL, cholesterol; GLU, glucose; GSRS, Gastrointestinal Symptoms Rating Scale; HOMA-IR, Homeostasis Model Assessment-Insulin Resistance; INU, inulin; INSU, insulin; PLA, placebo; P-type, *Prevotella*-dominant individuals; PYY, protein YY; SCFA, short-chain fatty acids; VAS, visual analog scales.

**
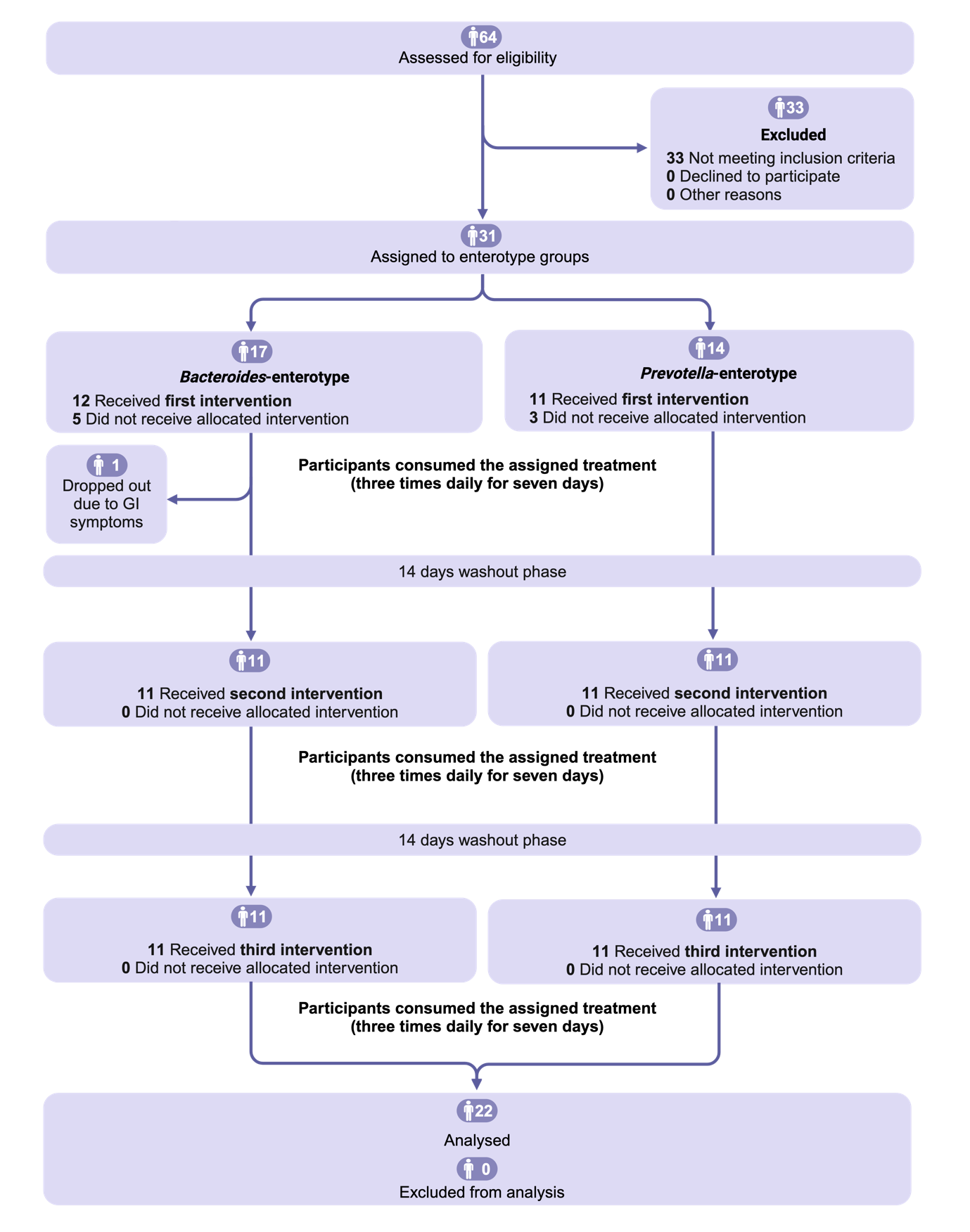
**

**Supplementary Figure 2**. CONSORT flow diagram illustrating participant enrollment, allocation, intervention, and analysis. GI, gastrointestinal.


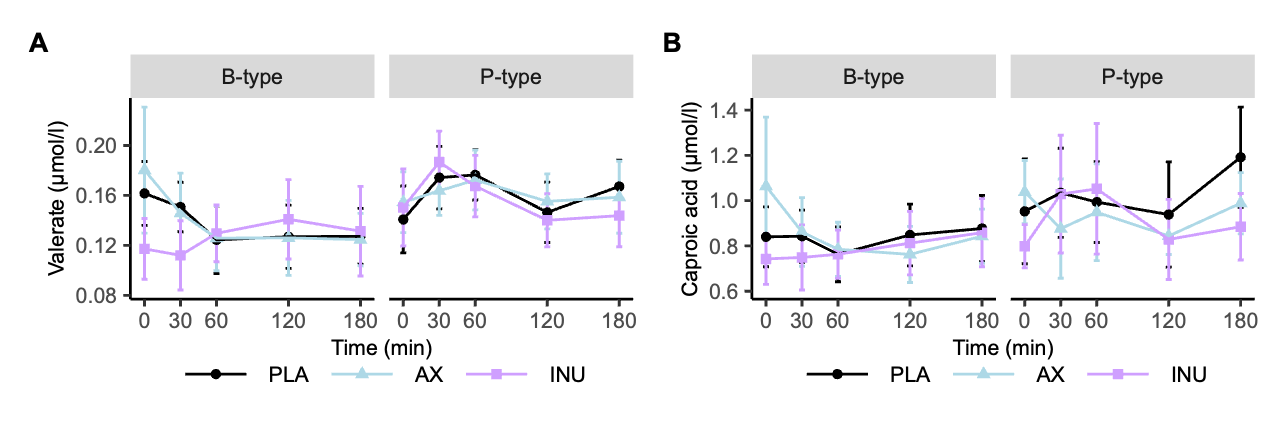


**Supplementary Figure 3**. Plasma short-chain fatty acids before and after standardized breakfast following AX, INU, and PLA supplementation in B- and P-types (n = 11 per group). (A) Plasma valerate, (B) plasma caproic acid. Data are presented as means ± S.E.M. Fasting and postprandial differences, based on the area under the curve (AUC), were analyzed using repeated-measures ANOVA. Post hoc pairwise t-tests were performed with LSD adjustment for p-values. B-type, Bacteroides-dominant individuals; INU, inulin; PLA, placebo; P-type, Prevotella-dominant individuals.


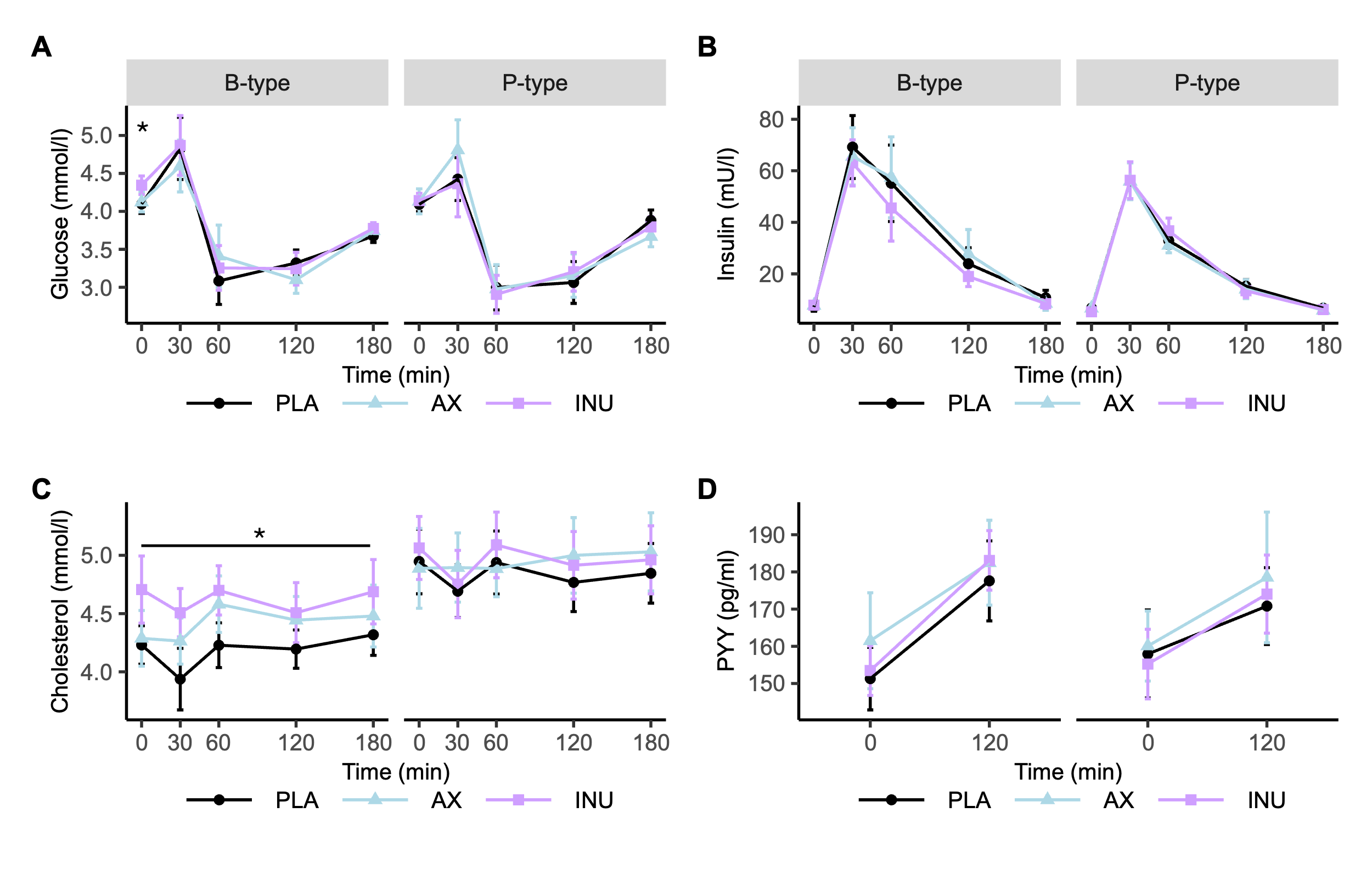


**Supplementary Figure 4.** Plasma metabolic markers before and after standardized breakfast following AX, INU, and PLA supplementation B- and P-types (n = 11 per group). (A) Plasma glucose, (B) plasma insulin, (C) plasma cholesterol, (D) plasma peptide YY (PYY). Data are presented as means ± S.E.M. Fasting and postprandial differences, based on the area under the curve (AUC), were analyzed using repeated-measures ANOVA. Post hoc pairwise t-tests were performed with LSD adjustment for p-values. * p < 0.05 INU vs. PLA, $ p < 0.05 AX vs. PLA, †p < 0.05 AX vs. INU. AX, arabinoxylan; B-type, Bacteroides-dominant individuals; INU, inulin; PLA, placebo; P-type, Prevotella-dominant individuals.
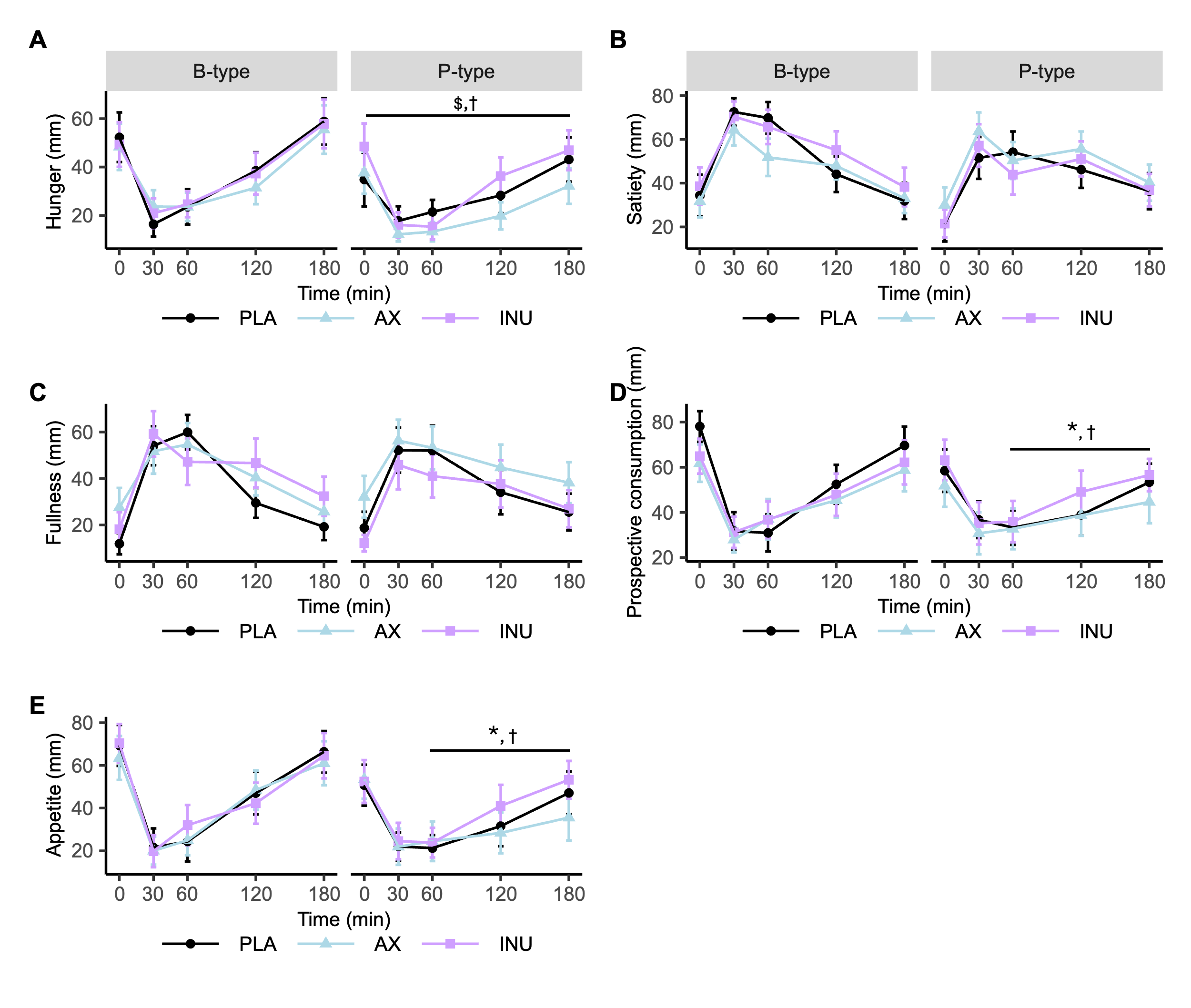


**Supplementary Figure 5.** **Subjective hunger and satiety ratings before and after standardized breakfast following AX, INU, and PLA supplementation** B- and P-types (n = 11 per group)**.** (A) Hunger (How hungry do you feel? Not hungry at all – As hungry as I have ever felt), (B) Satiety (How satisfied do you feel? Completely empty – Cannot eat another bite), (C) Fullness (How full do you feel? Not full at all – Totally full), (D) Prospective consumption (How much do you think you could eat right now? Not at all – A large amount), (E) Appetite (How strong is your desire to eat? Very weak – Very strong). Ratings were assessed using visual analog scales (VAS, 0–100 mm). Data are presented as means ± S.E.M. Fasting and postprandial differences, based on the area under the curve (AUC), were analyzed using repeated-measures ANOVA. Post hoc pairwise t-tests were performed with LSD adjustment for p-values. *p < 0.05 INU vs. PLA, $p < 0.05 AX vs. PLA. AX, arabinoxylan; B-type, Bacteroides-dominant individuals; INU, inulin; PLA, placebo; P-type, Prevotella-dominant individuals.

**
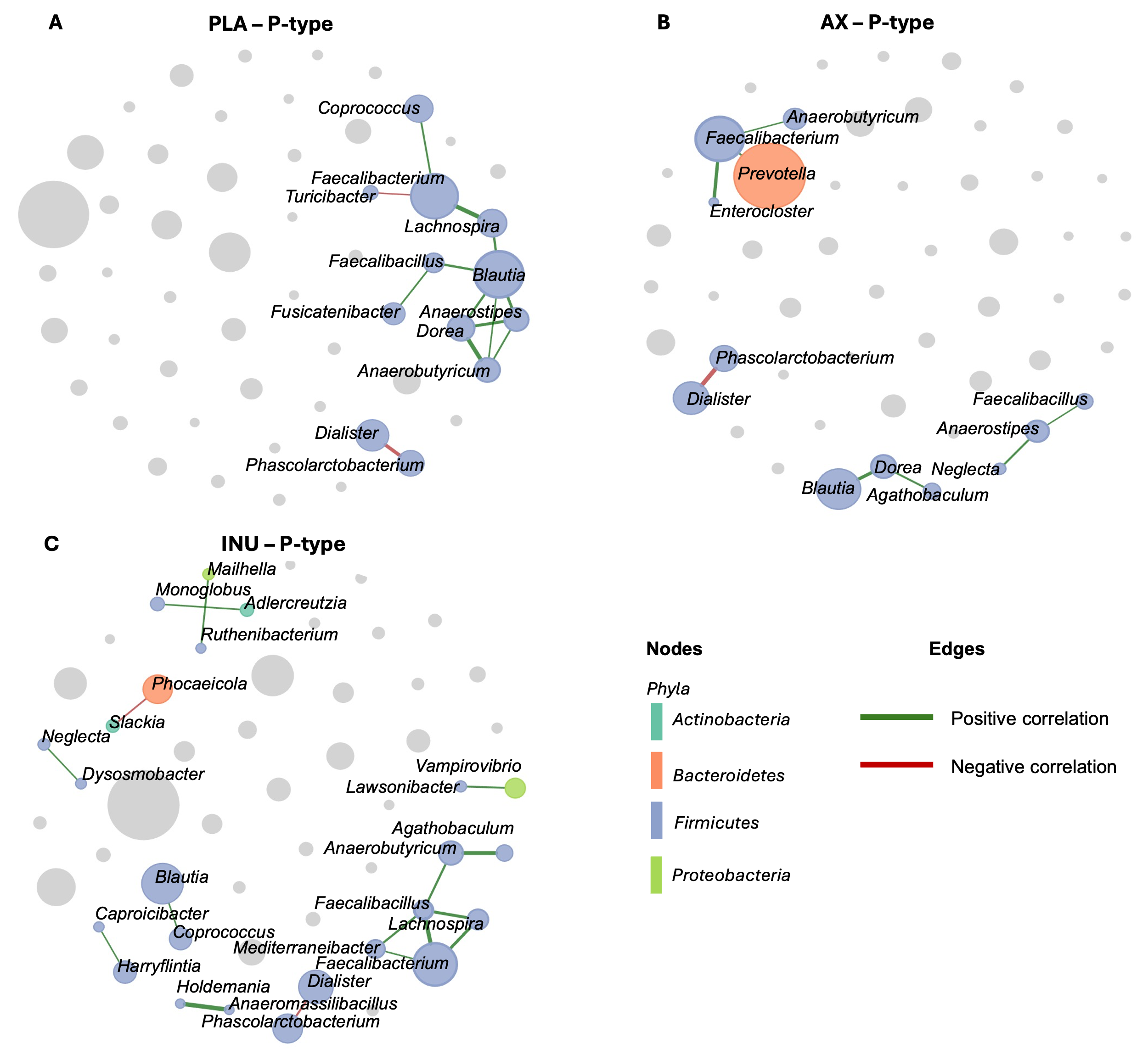
**

**Figure 6.** Microbial co-occurrence networks in P-types following PLA, AX, and INU (n = 11). Genus-level co-occurrence networks inferred via graphical LASSO after PLA (A), AX (B), and INU (C). Edge width indicates partial correlation coefficient (|ρ|), node size reflects square-root scaled mean relative abundance. Node colors indicate phylum-level affiliation. Only edges with |partial correlation| ≥ 0.02 are shown. AX, arabinoxylan; INU, inulin; PLA, placebo; P-type, Prevotella-dominant individuals.

**
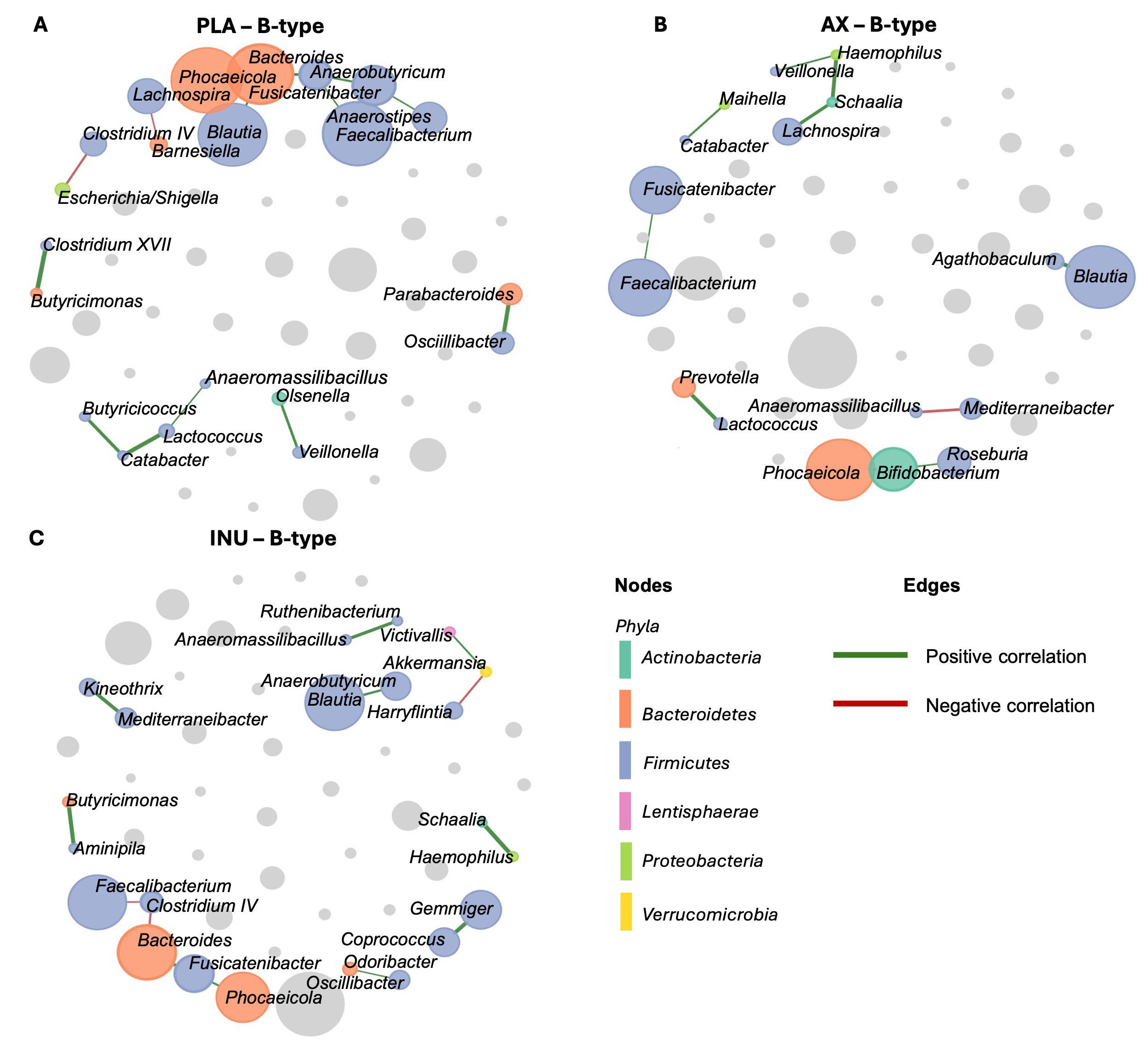
**

**Figure 7.** Microbial co-occurrence networks in B-types following PLA, AX, and INU (n = 11). Genus-level co-occurrence networks inferred via graphical LASSO after PLA (A), AX (B), and INU (C). Edge width indicates partial correlation coefficient (|ρ|), node size reflects square-root scaled mean relative abundance. Node colors indicate phylum-level affiliation. Only edges with |partial correlation| ≥ 0.02 are shown. AX, arabinoxylan; INU, inulin; PLA, placebo; P-type, Prevotella-dominant individuals.

**
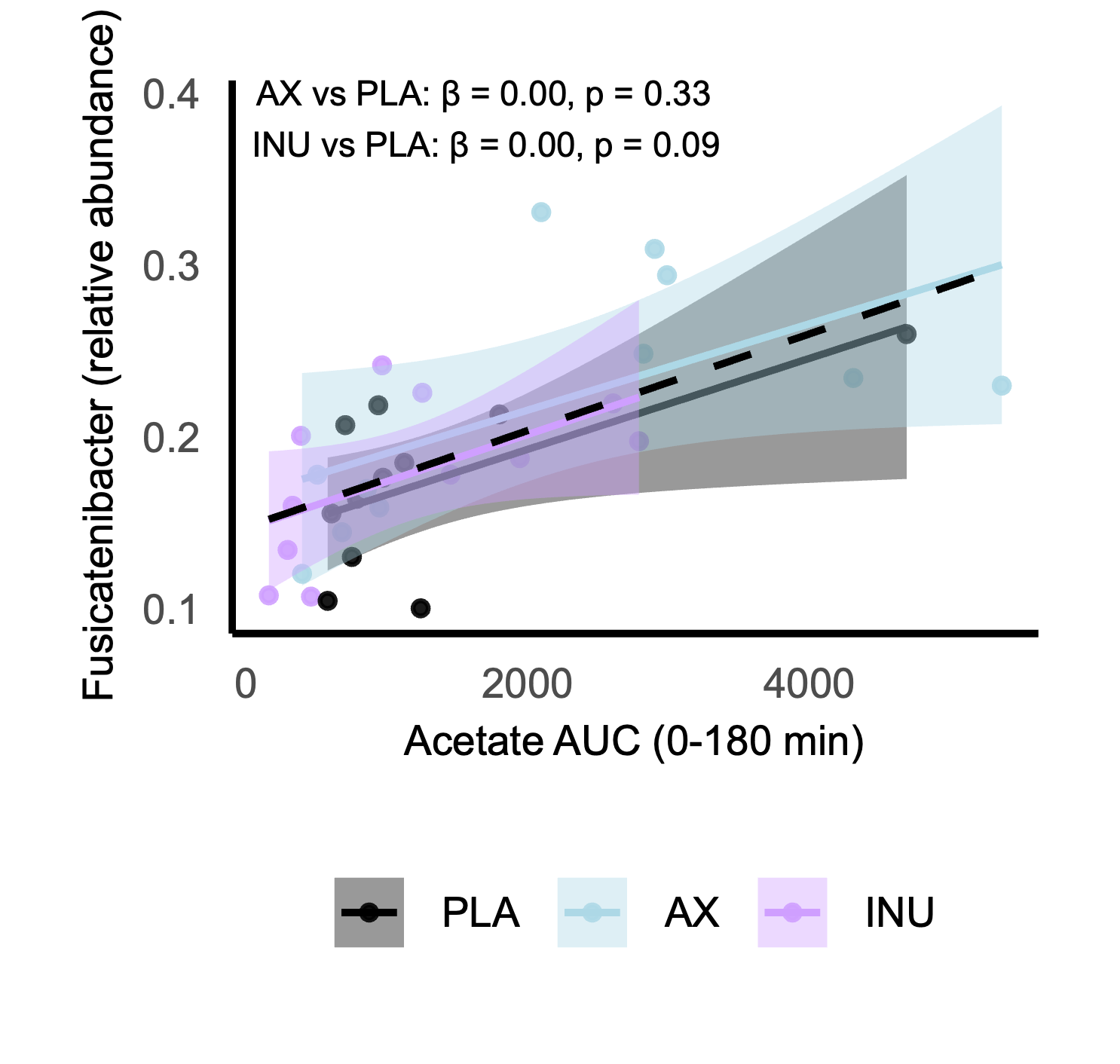
**

**Supplementary Figure 9.** Associations between microbial genera and postprandial acetate concentrations (AUC 0-180) across treatments within B-types (n = 11). Scatterplots show the relationship between the relative abundance of bacterial genera that showed significant associations in the Maaslin2 analysis and SCFA concentrations in different treatment groups (PLA, AX, INU). Linear regression lines with confidence intervals are displayed for each treatment group (solid lines) and for all samples combined (dashed line). The relative abundance data were transformed and normalized using Maaslin2 default settings (TSS normalization, AST transformation). Statistical associations were assessed using linear mixed models (LMM) with treatment as an interaction term and participant ID as a random effect. The β-coefficients and p-values for the interaction effects (AX vs. PLA and INU vs. PLA) are provided directly within the plots.


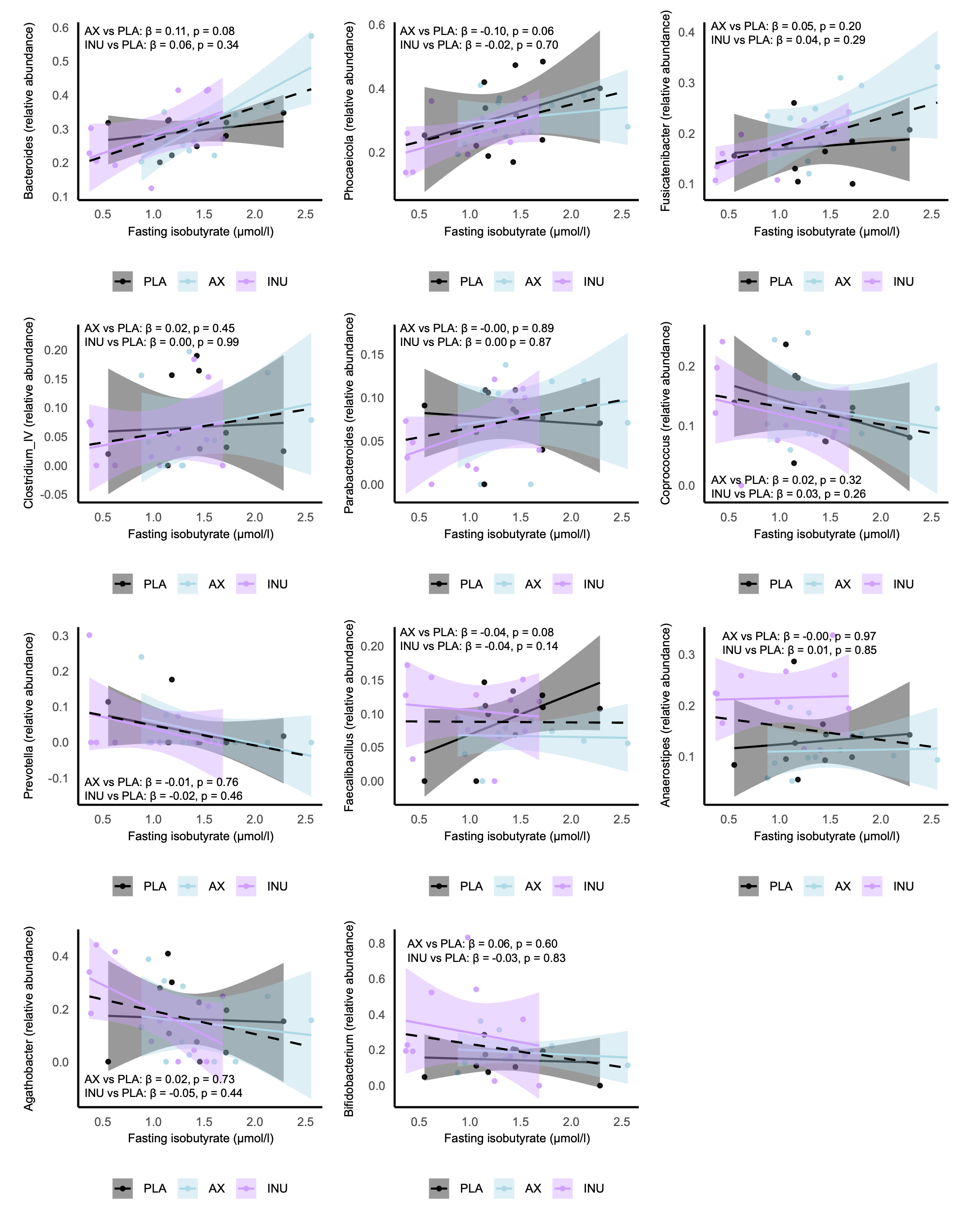


**Supplementary Figure 10.** Associations between microbial genera and fasting isobutyrate concentrations across treatments within B-types (n = 11). Scatterplots show the relationship between the relative abundance of bacterial genera that showed significant associations in the Maaslin2 analysis and SCFA concentrations in different treatment groups (PLA, AX, INU). Linear regression lines with confidence intervals are displayed for each treatment group (solid lines) and for all samples combined (dashed line). The relative abundance data were transformed and normalized using Maaslin2 default settings (TSS normalization, AST transformation). Statistical associations were assessed using linear mixed models (LMM) with treatment as an interaction term and participant ID as a random effect. The β-coefficients and p-values for the interaction effects (AX vs. PLA and INU vs. PLA) are provided directly within the plots.


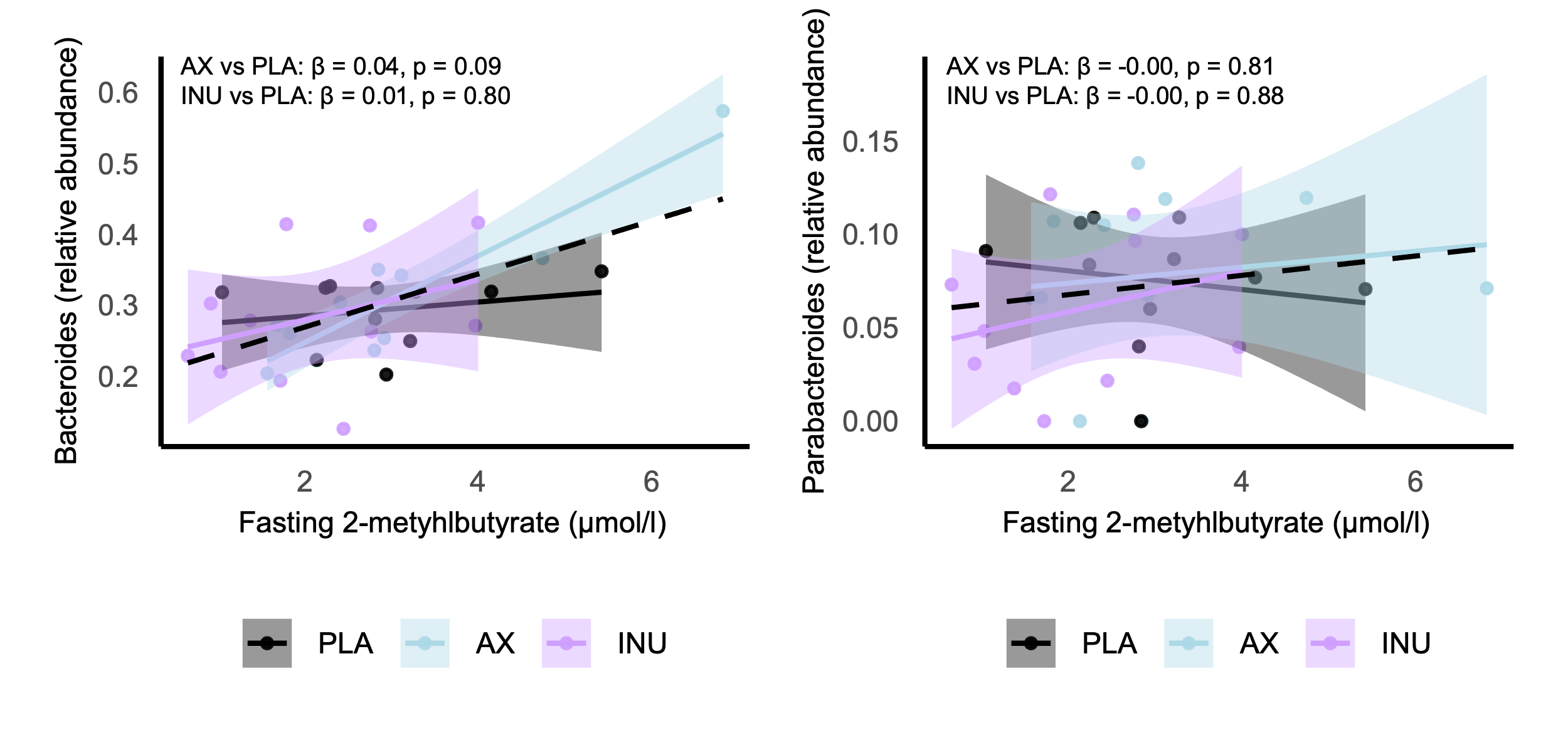


**Supplementary Figure 11.** Associations between microbial genera and fasting 2-methylbutyrate concentrations across treatments within B-types (n = 11). Scatterplots show the relationship between the relative abundance of bacterial genera that showed significant associations in the Maaslin2 analysis and SCFA concentrations in different treatment groups (PLA, AX, INU). Linear regression lines with confidence intervals are displayed for each treatment group (solid lines) and for all samples combined (dashed line). The relative abundance data were transformed and normalized using Maaslin2 default settings (TSS normalization, AST transformation). Statistical associations were assessed using linear mixed models (LMM) with treatment as an interaction term and participant ID as a random effect. The β-coefficients and p-values for the interaction effects (AX vs. PLA and INU vs. PLA) are provided directly within the plots.


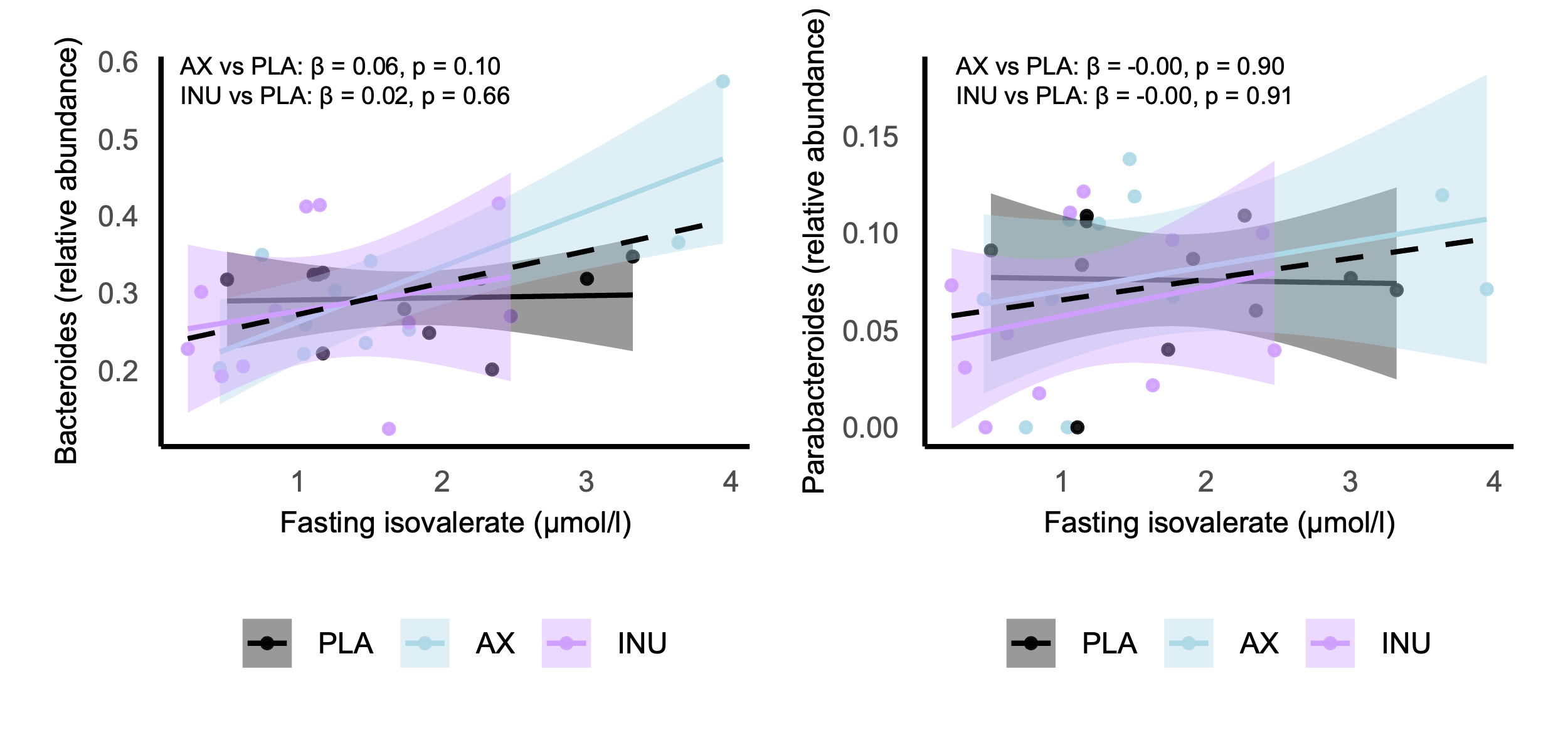


**Supplementary Figure 12.** Associations between microbial genera and fasting isovalerate concentrations across treatments within B-types (n = 11). Scatterplots show the relationship between the relative abundance of bacterial genera that showed significant associations in the Maaslin2 analysis and SCFA concentrations in different treatment groups (PLA, AX, INU). Linear regression lines with confidence intervals are displayed for each treatment group (solid lines) and for all samples combined (dashed line). The relative abundance data were transformed and normalized using Maaslin2 default settings (TSS normalization, AST transformation). Statistical associations were assessed using linear mixed models (LMM) with treatment as an interaction term and participant ID as a random effect. The β-coefficients and p-values for the interaction effects (AX vs. PLA and INU vs. PLA) are provided directly within the plots.


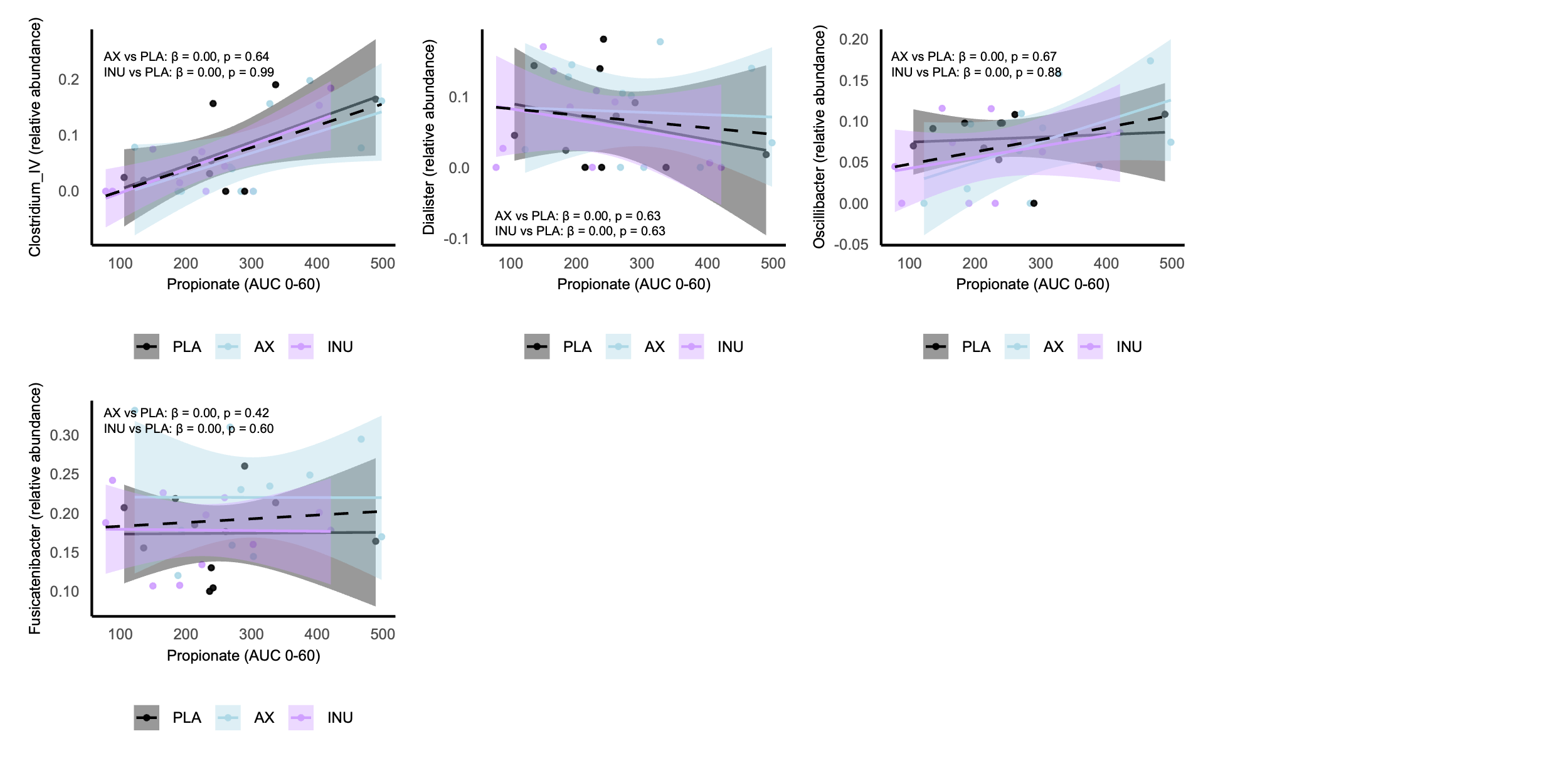


**Supplementary Figure 13.** Associations between microbial genera and postprandial propionate (AUC 0-60) concentrations across treatments within B-types (n = 11). Scatterplots show the relationship between the relative abundance of bacterial genera that showed significant associations in the Maaslin2 analysis and SCFA concentrations in different treatment groups (PLA, AX, INU). Linear regression lines with confidence intervals are displayed for each treatment group (solid lines) and for all samples combined (dashed line). The relative abundance data were transformed and normalized using Maaslin2 default settings (TSS normalization, AST transformation). Statistical associations were assessed using linear mixed models (LMM) with treatment as an interaction term and participant ID as a random effect. The β-coefficients and p-values for the interaction effects (AX vs. PLA and INU vs. PLA) are provided directly within the plots.


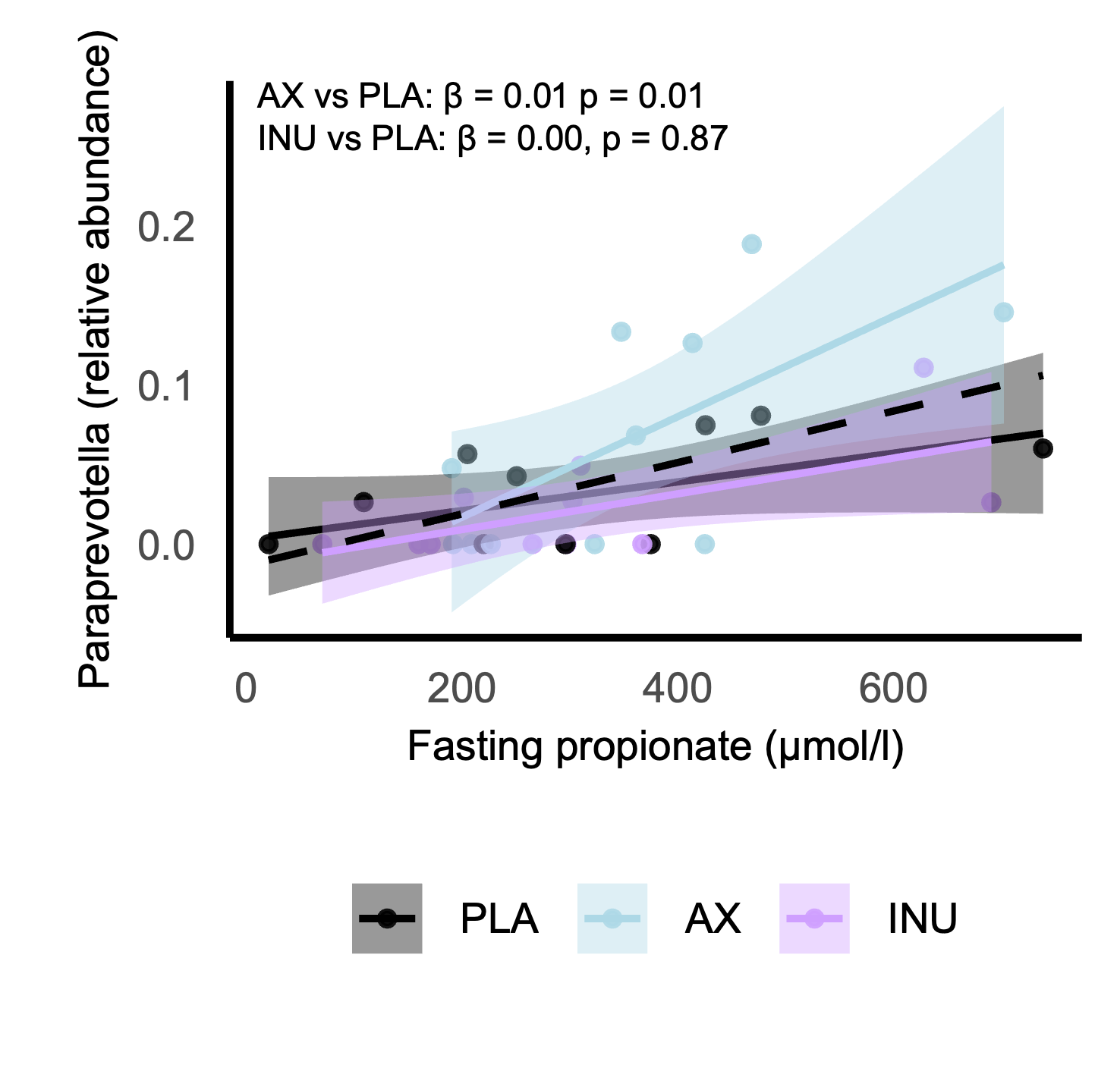


**Supplementary Figure 14.** Associations between microbial genera and fasting propionate concentrations across treatments within P-types (n = 11). Scatterplots show the relationship between the relative abundance of bacterial genera that showed significant associations in the Maaslin2 analysis and SCFA concentrations in different treatment groups (PLA, AX, INU). Linear regression lines with confidence intervals are displayed for each treatment group (solid lines) and for all samples combined (dashed line). The relative abundance data were transformed and normalized using Maaslin2 default settings (TSS normalization, AST transformation). Statistical associations were assessed using linear mixed models (LMM) with treatment as an interaction term and participant ID as a random effect. The β-coefficients and p-values for the interaction effects (AX vs. PLA and INU vs. PLA) are provided directly within the plots.
